# Simultaneous targeting of KRAS and CDK4 synergistically induces durable growth arrest in pancreatic cancer cells

**DOI:** 10.1038/s41419-025-08362-w

**Published:** 2025-12-23

**Authors:** Maj-Britt Paulsohn, Klara Henrike Frahnert, Denise Schlösser, Joana Oschwald, Waltraut Kopp, Xin Fang, Carolin Schneider, Constanza Tapia Contreras, Adi Danieli-Mackay, Fabian Ludewig, Martina Bleyer, Gabriela Salinas, Günter Schneider, Elisabeth Hessmann, Matthias Dobbelstein

**Affiliations:** 1 Department of Molecular Oncology, Göttingen Center of Molecular Biosciences (GZMB), University Medical Center Göttingen, Göttingen, Germany; 2 Clinical Research Unit 5002, KFO5002, University Medical Center Göttingen, Göttingen, Germany; 3 Department of Gastroenterology, Gastrointestinal Oncology and Endocrinology, University Medical Center Göttingen, Göttingen, Germany; 4 Department of General, Visceral and Pediatric Surgery, University Medical Center Göttingen, Göttingen, Germany; 5 Institute of Pathology, University Medical Center Göttingen, Göttingen, Germany; 6 NGS-Integrative Genomics (NIG), University Medical Center Göttingen, Göttingen, Germany; 7 Laboratory Animal Science Unit, Pathology, German Primate Center, Leibniz Institute of Primate Research, Göttingen, Germany; 8 CCC-N (Comprehensive Cancer Center Lower Saxony), Göttingen, Germany; 9 Max Planck Institute for Multidisciplinary Sciences, Göttingen, Germany

**Keywords:** Targeted therapies, Targeted therapies, Target validation

## Abstract

Mutant Ras oncoproteins, particularly KRAS, are among the most prevalent drivers of cancer. Small-molecule KRAS inhibitors have emerged as promising cancer therapeutics, yet resistance development remains a major hurdle. To overcome this challenge, we explored rational combination strategies aimed at enhancing therapeutic efficacy and durability. We show that the KRAS-G12C inhibitor Sotorasib synergizes with the CDK4/6 inhibitor Palbociclib to eliminate pancreatic ductal adenocarcinoma (PDAC) cells and organoids harboring KRAS-G12C mutations. This synergy was especially pronounced following drug washout, indicating a durable cellular response. Similar synergistic effects were observed in non-small-cell lung cancer (NSCLC) cells. Additionally, the KRAS-G12D inhibitor MRTX1133 cooperated with Palbociclib to suppress growth of KRAS-G12D-mutant PDAC cells. Mechanistically, the combinations induced sustained cell cycle arrest, marked by reduced RB phosphorylation, decreased E2F1 expression, and increased levels of CDKN1B/p27. Deletion of *CDKN1B* largely reversed the growth-inhibitory effect, highlighting its essential role in mediating the observed synergy. In an orthotopic, immunocompetent mouse model of PDAC, MRTX1133 significantly reduced tumor growth and extended survival; however, despite its ability to suppress RB phosphorylation, Palbociclib failed to enhance these effects. Single-cell RNA sequencing suggested that Palbociclib treatment induces tumor vascularization, perhaps contributing to the lack of drug synergy observed in vivo. In summary, our findings demonstrate the therapeutic potential of enhancing cell cycle restriction point activation in KRAS inhibitor-based therapies, while emphasizing the importance of placing combination therapies into a suitable context.

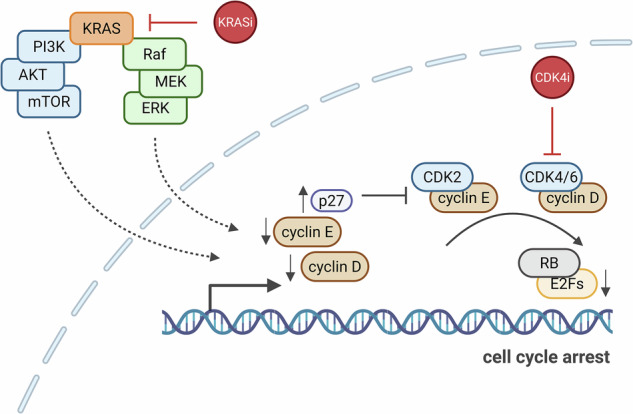

## Introduction

Mutant Ras proteins are among the most common drivers of human cancer [1], a fact recognized for over four decades [2]. Of the Ras protein family, KRAS is the most commonly mutated oncogene, particularly in pancreatic ductal adenocarcinoma (PDAC), non-small cell lung cancer (NSCLC), colorectal cancer and other malignancies [3]. Ras proteins function as GTP-binding proteins (G-proteins) that mediate receptor-driven signaling to intracellular kinases, notably through the RAF-MEK-ERK and PI3K-AKT-mTOR pathways. The majority of KRAS mutations occur at codon 12, with G12D, G12V, and G12C being the most prevalent [4]. These mutations impair GTP hydrolysis, thereby prolonging signaling activity and driving oncogenesis.

Despite the pivotal role of KRAS mutations in cancer progression, the development of effective inhibitors has been challenging [5]. Early attempts to inhibit KRAS through farnesylation interference were unsuccessful in clinical translation [6]. However, significant breakthroughs were achieved in targeting mutant KRAS [7, 8], which led to the introduction of Sotorasib, the first FDA-approved KRAS G12C inhibitor [9, 10]. Sotorasib covalently binds to the mutant cysteine residue in KRAS G12C, effectively inhibiting its activity [11]. Subsequent advancements led to the development of inhibitors targeting other KRAS mutations, such as G12D, offering new therapeutic opportunities for cancers like PDAC that are notoriously difficult to treat [12, 13].

Despite these advances, resistance to KRAS inhibitors has emerged as a significant hurdle [13]. This highlights the urgent need for strategies that achieve sustainable elimination of KRAS-mutant cancer cells through mechanisms such as inducing cell death or senescence, rather than transient cell cycle arrest. This challenge parallels earlier concerns with cyclin-dependent kinase 4 (CDK4) inhibitors, initially believed to induce only transient cell cycle arrest [14]. CDK4 phosphorylates and inactivates the RB family of tumor suppressor proteins, which suppress E2F-driven transcription and proliferation. While CDK4 inhibitors were initially met with skepticism, they have since become a cornerstone of cancer therapy, particularly in breast cancer [15, 16], though their precise mechanisms of action remain incompletely understood [17].

The concept of synthetic lethality in cancer therapy—combining targeted inhibitors to achieve permanent cancer cell elimination—was first proposed two decades ago [18]. This approach holds particular promise for KRAS inhibitors, as effective combination therapies may overcome resistance and enhance therapeutic outcomes [13, 19]. Based on this rationale, we hypothesized that KRAS and CDK4 inhibitors could form a synthetic lethal combination capable of eradicating cancer cells. Supporting this idea, preclinical studies have shown that combining CDK4 inhibitors with agents targeting downstream signaling pathways of KRAS effectively suppresses tumor growth in PDAC models [20–22]. With the recent availability of direct KRAS inhibitors, it becomes critical to investigate whether their combination with CDK4 inhibitors could produce synergistic therapeutic effects.

Here, we demonstrate that combined inhibition of KRAS and CDK4 synergistically and durably suppresses the proliferation and viability of PDAC and NSCLC cells across multiple experimental systems. Mechanistically, we show that CDKN1B/p27 plays a critical role in RB activation, sustaining prolonged cell cycle arrest. Furthermore, we show that the inhibitors induce remodeling of the tumor microenvironment (TME), which may critically influence their therapeutic efficacy.

## Results

### The KRAS mutant G12C-targeting drug Sotorasib synergizes with Palbociclib to suppress the growth of pancreatic cancer cells and organoids, with sustainability after drug removal

**Fig. 1:**
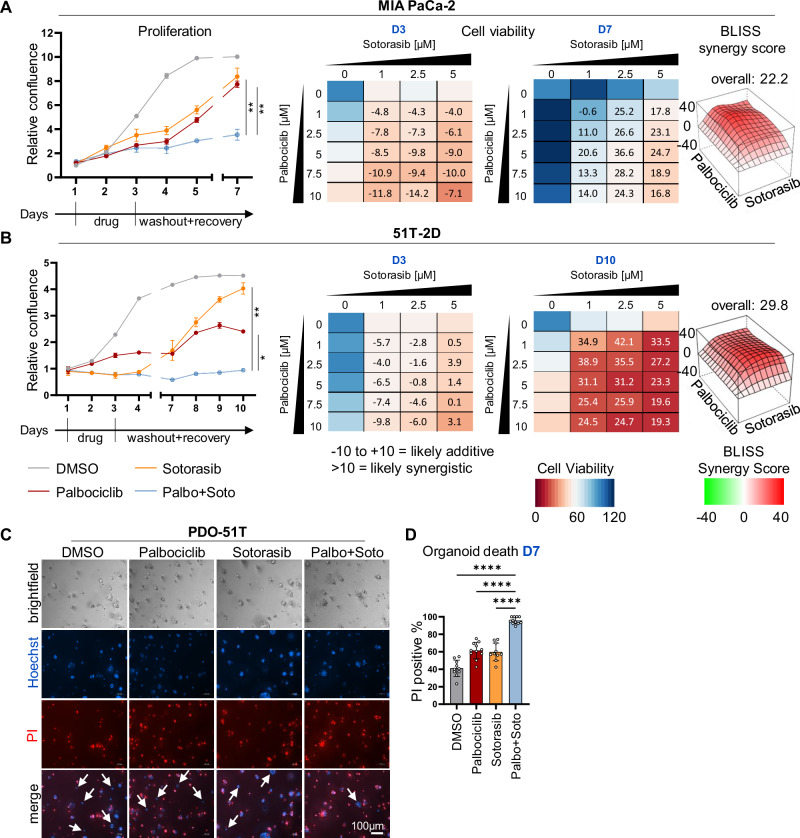
The KRAS mutant G12C-targeting drug Sotorasib synergizes with Palbociclib to suppress the growth of pancreatic cancer cells and organoids, with sustainability after drug removal. **A** (left) Proliferation of MIA PaCa-2 cells, determined by automated microscopy (Celigo®) upon treatment with DMSO, 5 µM Palbociclib, 2.5 µM Sotorasib, or the combination. Cells were treated for 48 h, followed by 4 days of recovery in the absence of drugs. The graphs indicate the means of three technical replicates ±SD. (right) Heat maps reflect the viability of MIA PaCa-2 cells right after treatment (D3) and after recovery (D7), normalized to the DMSO controls. Bliss synergy score values were determined based on cell viability. BLISS values > 10 likely reflect synergy, whereas values between −10 and 10 correspond to an additive mechanism. The BLISS synergy map of D7 is depicted. **B** (left) Proliferation of 51T-2D cells upon treatment as in (**A**) (right) Heat maps show the viability of 51T-2D cells right after treatment (D3) and after recovery (D10), normalized to the DMSO control. BLISS synergy map of D10. **C** PDO-51T organoids (KRAS G12C) treated with 5 µM Palbociclib and 2.5 µM Sotorasib for 48 h, followed by 4 days of recovery without drugs. Representative brightfield images of day 7 (D7) as well as Hoechst 33342 and PI staining are presented. Scale bar 100 µm. **D** Hoechst 33342 and PI staining was used to quantify the viability of organoids at day 7 (D7). Statistical analyses: **A**, **B** unpaired t-test (**A**, **B** of AUC); D, one-way ANOVA followed by Tukey’s multiple comparison; ns not significant, *p ≤ 0.05, **p ≤ 0.01, ***p ≤ 0.001, ****p ≤ 0.0001.

Previous studies pointed to CDK4 as a promising combination partner for KRAS inhibitors to eliminate PDAC cells. This became evident when we re-analyzed a published CRISPR interference (CRISPRi) screen of MIA PaCa-2 cells treated with the KRAS G12C inhibitor ARS-1620 [23]. The analysis revealed that the deletion of cyclin D1 (CCND1) or its interaction partner CDK4 each conferred some of the strongest sensitization towards the drug (Supplementary Fig. 1A). Consistent with these findings, combining inhibitors of MEK/MAPK2 [20, 22] or ERK/MAPK3 [21, 24, 25] with CDK4 inhibition has been shown to effectively eliminate cancer cells of entities such as PDAC or multiple myeloma. Based on this evidence, we investigated whether the simultaneous inhibition of KRAS and CDK4 could synergistically suppress PDAC cell proliferation. Initially, we picked the most established KRAS inhibitor, Sotorasib (also known as AMG510), which specifically targets the G12C mutant of KRAS through covalent modification [7, 11, 26]. Indeed, treating MIA PaCa-2 cells with Sotorasib along with Palbociclib profoundly suppressed cell confluence as well as viability (Fig. 1A and Supplementary Fig. 1B). Notably, the synergy was even more pronounced 4 days after drug removal, indicating sustainable efficacy of the drug combination. This was confirmed by the BLISS synergy score. Similar observations were made using the cell line 51T-2D, which we had established from a patient-derived organoid (PDO) of PDAC [27]. Like MIA PaCa-2 cells, 51T-2D cells carry the KRAS G12C mutation, which is otherwise rare in PDAC, thus providing an additional opportunity to test the impact of Sotorasib and its cooperation with Palbociclib. Here again, we observed sustained growth arrest and drug synergy (Fig. 1B and Supplementary Fig. 1C). Like Palbociclib, the CDK4 inhibitor Abemaciclib diminished cell proliferation and viability in combination with Sotorasib in MIA PaCa-2 and 51T-2D cells (Supplementary Fig. 1D–G). In contrast, and as expected, Sotorasib did not modulate the impact of Palbociclib on KPC cells, which carry the KRAS G12D mutation (Supplementary Fig. 1H). In Sotorasib-susceptible cells, however, the drug combination diminished the proportion of cells undergoing DNA replication, as revealed by incorporation of a nucleoside analog (Supplementary Fig. 1I, J). Analysis of the DNA content by flow cytometry indicated sustained G1 arrest during and after treatment, in particular with Palbociclib or the drug combination (Supplementary Fig. 1K, L). The drugs, in particular when combined, also enhanced the fraction of dead cells, as assayed by annexin V staining, by uptake of propidium iodide, and by PARP1 cleavage (Supplementary Fig. 1M–O). We also combined Palbociclib with inhibitors that target signaling factors downstream of KRAS, including the MEK inhibitor Trametinib and the ERK inhibitor SCH984. Such inhibitors also displayed synergy with the CDK4 inhibitor, albeit to a lesser degree than KRAS inhibitors in the investigated experimental settings (Supplementary Fig. 1P, Q).

Since tumor cell organoids often reflect the behavior of actual tumors more closely than 2D-cultures, we also investigated the impact of Sotorasib and Palbociclib on the survival of PDO-51T. Indeed, the drugs cooperated to enhance the uptake of propidium iodide, indicating enhanced death of organoid cells (Fig. 1C, D). We conclude that the combination of Sotorasib and a CDK4 inhibitor strongly synergizes to compromise the proliferation and viability of PDAC cells and organoids.

### Palbociclib and KRAS inhibitors synergize to suppress PDAC and NSCLC cell proliferation

**Fig. 2:**
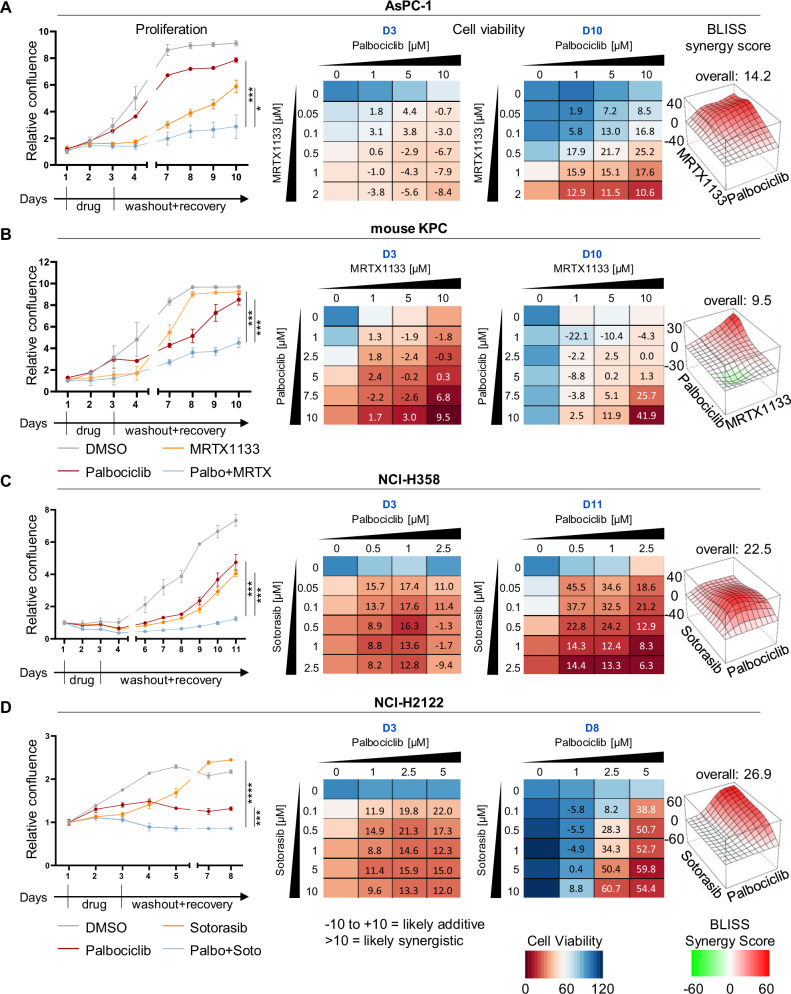
Palbociclib and KRAS inhibitors synergize to suppress PDAC and NSCLC cell proliferation. **A**–**D** All cells were treated and observed as in Fig. 1, with the following specifics: **A** AsPC-1 cells (human PDAC), 5 µM Palbociclib and 0.5 µM MRTX1133, measurements at D3 and D10. **B** KPC cells (murine PDAC), 5 µM Palbociclib, 5 µM MRTX1133, evaluation at D3 and D10. **C** NCl-H358 cells (human NSCLC), 0.5 µM Palbociclib, 50 nM Sotorasib, evaluation at D3 and D10. **D** NCl-H2122 (human NSCLC), 2.5 µM Palbociclib, 5 µM Sotorasib, evaluation at D3 and D8. Statistical analyses: **A**–**D** unpaired t-test (**A**–**D**: of AUC); ns not significant, *p ≤ 0.05, **p ≤ 0.01, ***p ≤ 0.001, ****p ≤ 0.0001.

Sotorasib exclusively targets the KRAS mutant G12C, which is rare in PDAC. To raise a perspective for broader treatment options, we evaluated a similar approach with additional pancreatic cancer cells that carry the more frequently encountered mutation G12D in KRAS. At present, inhibitors of KRAS G12D have not yet been approved for clinical use. However, the KRAS G12D inhibitor MRTX1133 [28, 29] is currently tested in phase I and II trials for advanced solid tumors (NCT05737706). We evaluated the effect of inhibiting KRAS G12D and CDK4, alone or in combination. MRTX1133 and Palbociclib diminished the proliferation and the viability of human AsPC-1 and murine KPC cells (Fig. 2A, B and Supplementary Fig. 2A, B). Synergy was revealed by the Bliss score, in particular, 7 days after drug removal. In addition, we analyzed cell lines established from patient-derived PDAC xenografts (GöCDX7, GöCDX52 and GöCDX53) that harbor KRAS G12D. GöCDX52 cells responded synergistically towards Palbociclib and MRTX1133, with significantly impaired cell proliferation and viability, again particularly seven days after drug removal (Supplementary Fig. 2E). GöCDX7 and GöCDX53 showed reduced growth paired with synergistic effects on viability, and this was confirmed by the BLISS score, too (Supplementary Fig. 2F, G). The pan-KRAS inhibitor RMC-7977 suppressed proliferation when combined with CDK4 inhibition to treat KRAS G12V mutant Capan-1 and Capan-2 cells, albeit to different degrees (Supplementary Fig. 2H, I). Furthermore, RMC-7977 and Palbociclib synergized to suppress the growth and viability of a KRAS wildtype leukemia cell line, MOLM-13 (Supplementary Fig. 2J). MOLM13 cells contain an amplification of *FLT3*, which encodes a tyrosine kinase upstream of KRAS. Accordingly, the FLT3 inhibitor AC220 displayed a similar synergy with Palbociclib on these cells (Supplementary Fig. 2K). Again, these results suggest that combining inhibitors of KRAS and CDK4 represents a synergistic and sustainable strategy to treat KRAS G12D/V mutant PDAC.

Besides PDAC, NSCLC is another entity with a high proportion of KRAS mutations. Here, the mutation G12C is most prominent, justifying Sotorasib monotherapy according to a recent study [10]. However, when treating the NSCLC-derived cell lines NCl-H358 and NCl-H2122 with Sotorasib, the cells were transiently stalled but resumed proliferation within a few days; in contrast, additional CDK4 inhibition largely prevented cell outgrowth (Fig. 2C, D and Supplementary Fig. 2C, D). Once again, we observed that the combination of KRAS and CDK4 inhibition strongly diminished cell proliferation and viability. Synergistic effects were observed already after 48 h of treatment, and this synergy became more robust after the additional recovery period. In summary, we provide evidence of sustainable and synergistic growth arrest by inhibiting CDK4 and mutant KRAS in PDAC and NSCLC cells.

In AsPC-1 cells, the drugs Palbociclib and MRTX1133 not only gave rise to profound cell cycle arrest (Supplementary Fig. 2L), but also to a substantial extent of cell death, as displayed by their sub-G1 DNA content (Supplementary Fig. 2M). Cleavage of PARP1 revealed apoptosis, albeit to a similar degree for MRTX1133 alone or in combination with Palbociclib (Supplementary Fig. 2N). Senescence-associated beta-galactosidase (SAB) staining was also enhanced upon the combined treatment, more than with either drug alone (Supplementary Fig. 2O, P), and this was true when treating NCl-H358 cells with Sotorasib and Palbociclib, too (Supplementary Fig. 2Q, R). Thus, the drug combinations prevent cell proliferation and viability by inducing long-term cycle arrest and/or apoptosis, to varying degrees depending on the particular cell context.

### Inhibitors of KRAS G12C and CDK4 cooperate to switch the transcriptome towards ceased proliferation

**Fig. 3:**
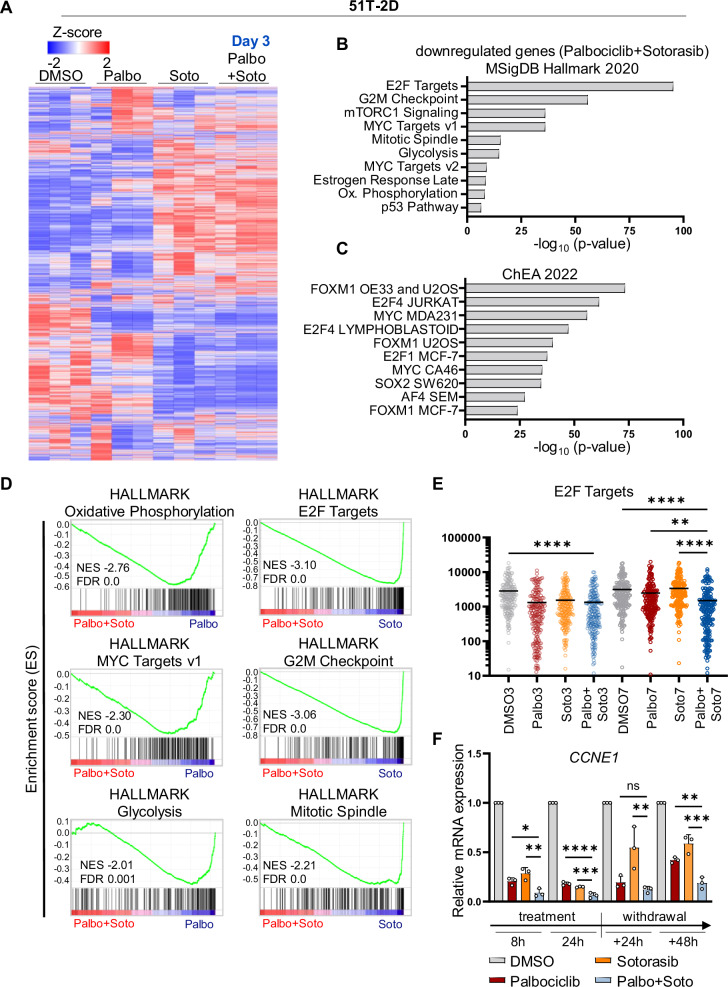
Inhibitors of KRAS G12C and CDK4 cooperate to switch the transcriptome towards ceased proliferation. **A** Heat map depicting differentially expressed (DE) genes according to the z-score after performing DeSeq2 analysis of four different samples (DMSO, 5 µM Palbociclib, 2.5 µM Sotorasib or combination treatment for 48 h (Day3), n = 3) in 51T-2D cells. Only genes with |log2fold| ≥ 0.6, adjusted p-value (padj.) <0.05, and baseMean ≥15 were included in the analysis. Supplementary Table 1 contains DE genes and normalized read counts. **B** Negatively regulated genes in response to Palbociclib + Sotorasib (48 h treatment) vs DMSO were correlated with the Molecular Signature Database (MSigDB) Hallmark 2020 and ChEA 2022 (**C**) dataset using the Enrichr platform to identify potentially impaired pathways. Top 10 (ChEA 2022: human only), p-value ranked (−log10). **D** Gene set enrichment analysis (GSEA) of combination treatment vs. Palbociclib or Sotorasib monotreatment, after 48 h of treatment, determining hallmarks (h.all.v2023.2). **E** Normalized counts of E2F targets upon 48 h treatment (3) or 48 h treatment + 4 days of drug withdrawal (7). **F** Expression of E2F target gene *CCNE1* in 51T-2D cells treated with 5 µM Palbociclib, 2.5 µM Sotorasib or the combination, for 8 and 24 h, and drug withdrawal for 24 or 48 h. Data were normalized to *36B4* mRNA and is shown as mean ± SD. Statistical analyses: **E**, **F** one-way ANOVA followed by Tukey’s multiple comparison; ns not significant, *p ≤ 0.05, **p ≤ 0.01, ***p ≤ 0.001, ****p ≤ 0.0001. Complete statistics in Supplementary Fig. 3C.

In search for mechanisms that underlie the observed synergy between inhibitors of KRAS G12C and CDK4, we analyzed mRNA levels in 51T-2D cells by deep sequencing, both immediately after treatment (Day3), and 4 days after washing off the drugs (Day7; PCA: Supplementary Fig. 3A, B and Supplementary Table 1). For day 3, the heat map of the z-scores depicts profound changes in gene expression between Palbociclib, Sotorasib and the combination treatment (Fig. 3A). Differentially expressed genes upon combination treatment were correlated with the Molecular Signature Database (MSigDB). This revealed negative regulation of genes corresponding to the signatures of E2F targets, the G2M checkpoint, mTORC1 signaling as well as MYC targets (Fig. 3B). Moreover, analysis of associated transcription factors pointed out E2F1, E2F4, MYC and FOXM1 (Fig. 3C). Gene set enrichment analyses (GSEA) identified similar pathways in the comparison between the single use of either Palbociclib or Sotorasib with the combination of both drugs (Fig. 3D). In addition, we found genes related to glycolysis and oxidative phosphorylation to be suppressed upon combination treatment. Normalized counts of E2F targets were significantly diminished, immediately after treatment and also after 4 days of washout (Fig. 3E and Supplementary Fig. 3C). Quantitative RT-PCR analyses confirmed that both inhibitors suppressed genes that need to be activated before the cell passes through the cell cycle checkpoints G1 → S or G2 → M, e.g., *CCNE1*/Cyclin E1 (Fig. 3F), *CCND1*/Cyclin D1, *BIRC5*/Survivin, *CCNB1*/Cyclin B1, and *PLK4*. Importantly, when combining both drugs, the suppression of these genes was largely maintained even 48 h or 4 days after drug removal. In contrast, the single drugs, when applied to the cells and then removed, only transiently diminished the expression of the same genes and allowed their re-appearance after washout. These observations were made in 51T-2D (Supplementary Fig. 3D, E), MIA PaCa-2 (Supplementary Fig. 3F) and NCl-H358 cells (Supplementary Fig. 3G). We conclude that the combined inhibition of KRAS and CDK4 sustainably suppresses the expression of genes that would be necessary to resume cell proliferation.

Similarly to the transcriptome analysis immediately after treatment, we performed RNA sequencing after 4 days of an additional washout phase and displayed the z-scores to compare expression levels (Supplementary Fig. 3H). Here, we found further evidence for a permanent cell cycle arrest in the maintained suppression of gene sets with E2F targets and the G2M checkpoint (Supplementary Fig. 3I, J). Cells that had been treated individually with Palbociclib or Sotorasib re-expressed E2F target genes; Sotorasib-treatment with washout resulted in even stronger expression of these genes than control treatment. In contrast, GSEA of cells receiving the combination treatment continuously repressed genes of the categories E2F target, G2M checkpoint or MYC target (Supplementary Fig. 3K). All these gene sets and transcription factors are indispensable for cell cycle progression, corroborating our observations of permanent cell cycle arrest induced by the drug combination.

### Transcriptome alterations in PDAC cells treated with Palbociclib and MRTX1133

**Fig. 4:**
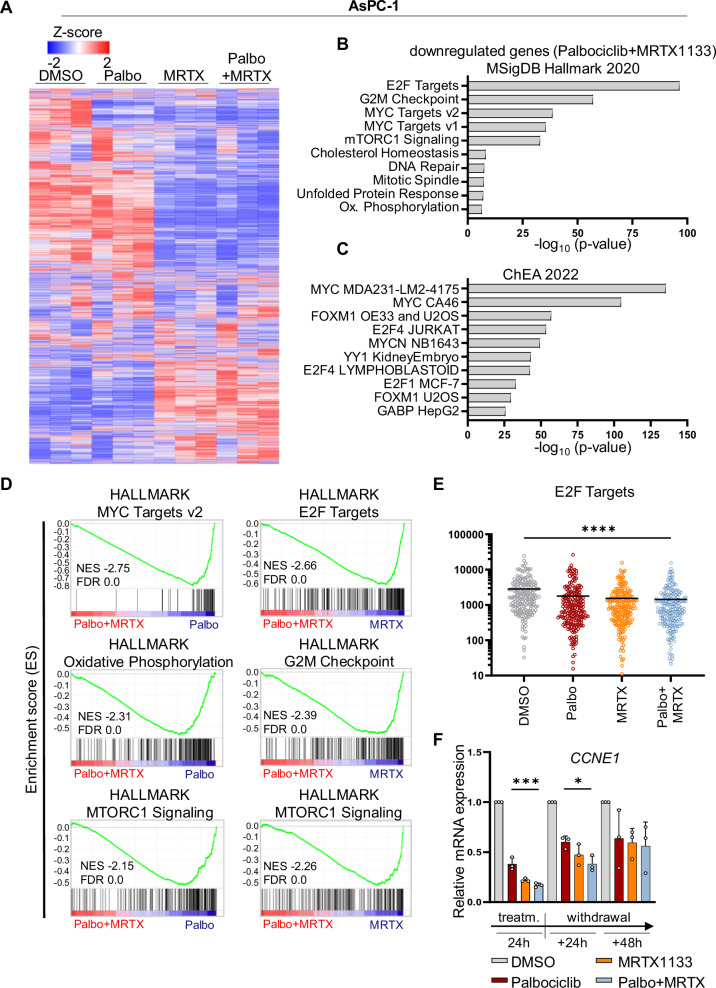
Transcriptome alterations in PDAC cells treated with Palbociclib and MRTX1133. **A** Heat map depicting DE genes according to the z-score after performing DeSeq2 analysis of four different samples (DMSO, 5 µM Palbociclib, 0.5 µM MRTX1133, or combination treatment, for 24 h (Day2), n = 3) in AsPC-1 cells. Only genes with |log2fold| ≥ 0.6, adjusted p-value (padj.) <0.05, and baseMean ≥15 were included in the analysis. Supplementary Table 2 contains DE genes and normalized read counts. **B** Downregulated genes in Palbociclib + MRTX1133 (24 h treatment) vs. DMSO were correlated with the Molecular Signature Database (MSigDB) Hallmark 2020 and ChEA 2022 (**C**) datasets, using the Enrichr platform to identify potentially impaired pathways. Top 10 (ChEA 2022: human only), p-value ranked (−log10). **D** Gene set enrichment analysis (GSEA) of combination treatment vs Palbociclib or MRTX1133 monotherapy after 24 h of treatment; hallmarks (h.all.v2023.2). **E** Normalised counts of E2F targets upon 24 h treatment. **F** Expression of the E2F target gene *CCNE1* in AsPC-1 cells treated with 5 µM Palbociclib, 0.5 µM MRTX1133, or the combination, for 24 h, and drug withdrawal for 24 or 48 h. mRNA levels were normalized to *36B4* mRNA, mean ± SD. Statistical analyses: **E**, **F** one-way ANOVA followed by Tukey’s multiple comparison; ns not significant, *p ≤ 0.05, **p ≤ 0.01, ***p ≤ 0.001, ****p ≤ 0.0001. Complete statistics in Supplementary Fig. 4B.

To further extend our mechanistic understanding of the cooperative inhibition of CDK4 and KRAS in PDAC cells, we analyzed the transcriptome of AsPC-1 cells treated for 24 h with Palbociclib and the KRAS G12D inhibitor MRTX1133 (PCA: Supplementary Fig. 4A and Supplementary Table 2). The heat map of the z-scores displays similarities between control and Palbociclib on the one hand and MRTX1133 and the combined treatment on the other hand (Fig. 4A). Genes suppressed by both drugs correlate with E2F targets, G2M checkpoint, MYC targets, and mTORC1 signaling, based on MSigDB (Fig. 4B). Similar to the transcriptome analysis obtained with Palbociclib and Sotorasib (Fig. 3), the combination of Palbociclib with MRTX1133 revealed transcription factors E2F1, E2F4, MYC, and FOXM1 to be associated with genes that were suppressed by the combination (Fig. 4C). In addition, GSEA found MYC targets, E2F targets, G2M checkpoint and mTORC1 signaling negatively enriched in cells treated with the combination (Fig. 4D). This is flanked by reduced normalized counts of E2F targets when combining Palbociclib and MRTX1133 (Fig. 4E and Supplementary Fig. 4B). Once again, we performed quantitative RT-PCR analyses to confirm that both drugs significantly impair the expression of genes required for the G1 → S or G2 → M transitions, e.g., *CCNE1*/Cyclin E1 (Fig. 4F). While single drugs only transiently repressed these genes, the combination diminished the expression of genes such as *BIRC5*/Survivin, *BRCA1* and *CCNB1*/Cyclin B1 even after 48 h of drug withdrawal (Supplementary Fig. 4C). Interestingly, genes of the CDKN2 family of CDK inhibitors were expressed more strongly immediately after treatment with Palbociclib and MRTX1133 (Supplementary Fig. 4D), while in Palbociclib and Sotorasib treated 51T-2D cells the CDKN1 family genes were enriched (Supplementary Fig. 3L), perhaps providing additional growth-inhibitory mechanisms. Curiously, in the presence and absence of Palbociclib, MRTX1133 induced genes associated with interferon responses (Supplementary Fig. 4E, F), suggesting a potential of KRAS inhibition to trigger an inflammatory response. In conclusion, we find Palbociclib and MRTX1133 to suppress genes required to overcome cell cycle checkpoints and to foster continuous proliferation.

### Upon inhibition of KRAS and CDK4, E2F1 largely disappears while CDKN1B/p27 levels increase and RB family proteins adopt a hypophosphorylated state

**Fig. 5:**
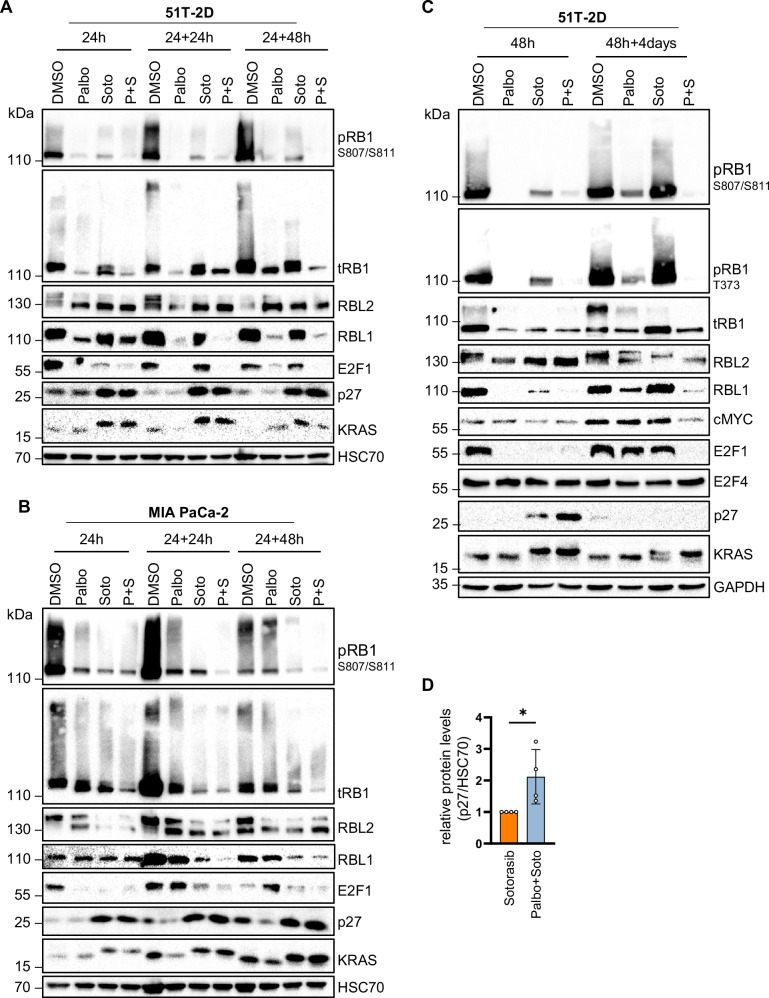
Upon inhibition of KRAS and CDK4, E2F1 largely disappears while CDKN1B/p27 levels increase and RB family proteins adopt a hypophosphorylated state. **A** Immunoblot analysis of whole-cell lysates of 51T-2D cells treated with DMSO, 5 µM Palbociclib, 2.5 µM Sotorasib, or both, for 24 h and wash-out for 24 or 48 h. HSC70 was detected as sample control. One representative immunoblot is shown; n = 2. Sotorasib covalently binds to KRAS, reflected by an electrophoretic mobility shift. **B** Immunoblot analysis as in (**A**), using MIA PaCa-2 cells treated with DMSO, 10 µM Palbociclib, 5 µM Sotorasib or the combination; n = 2. **C** Immunoblot analysis 51T-2D cells treated as in (**A**) for 48 h or 48 h + 4 days of drug withdrawal; n = 2. **D** Quantification of p27 protein levels in relation to loading control (HSC70) corresponding to (**C**); n = 4. Statistical analyses: **D**, unpaired t-test; ns not significant, *p ≤ 0.05, **p ≤ 0.01, ***p ≤ 0.001, ****p ≤ 0.0001.

To further understand how the two drugs achieve sustainable proliferation arrest, we assessed protein levels and phosphorylation states by immunoblot analyses. Immediately upon treatment, both drugs suppressed the phosphorylations that their targets or downstream signaling mediators would otherwise confer: ERK and AKT phosphorylation was diminished by Sotorasib, and RB1 phosphorylation by Palbociclib, in 51T-2D and MIA PaCa-2 cells (Supplementary Fig. 5A, B). After drug removal for another 24 or 48 h, however, the drug combination had a more sustainable impact on cell cycle regulators than the single drugs. In particular, the phosphorylation of RB1 was still reduced when both drugs were combined, and the overall levels of RB1 and RBL1/p107 were also suppressed. In contrast, RBL2/p130 was augmented under the same conditions (Fig. 5A, B). Remarkably, RBL2 is a key member of the repressive dimerization partner, RB-like, E2F and multi-vulval class B (DREAM) complex that represses the transcription of genes such as *BIRC5*, *BRCA1*, *CCNB1*, and *PLK4* [30, 31], i.e. the same proliferation-associated genes that we had found suppressed by the drug combination (Supplementary Figs. 3D–G and 4C). Similar observations were made in NCl-H358 cells (NSCLC; Supplementary Fig. 5C) or when using the KRAS G12D antagonist MRTX1133 together with Palbociclib in AsPC-1 cells (PDAC; Supplementary Fig. 5D). Furthermore, our analyses revealed that the levels of the key activating member of the E2F transcription factor family, E2F1, were reduced upon Palbociclib treatment, but even more strongly upon combined drug treatment (Fig. 5C). In particular, 4 days after drug removal, E2F1 levels remained low upon treatment with the combination, while E2F1 was re-synthesized upon monotherapy. E2F1 is a known activator of the above-named genes [32]. In addition, the mRNA encoding the cell cycle regulator CDKN1B/p27 was upregulated upon Sotorasib and combined treatment (Supplementary Figs. 3D–G and 4C). However, the p27 protein levels appeared more strongly elevated upon combination treatment for 48 h (Fig. 5D). In summary, these analyses elucidate the impact of the drug combinations on the transcriptome as follows: the combined reduction in E2F1 levels and the concurrent increase in the DREAM complex ensure sustained suppression of genes essential for bypassing cell cycle checkpoints. Additionally, the elevated levels of the cell cycle inhibitor p27 reinforce this effect. Consequently, the cells remain arrested in response to the drug combination treatment.

### Growth suppression by inhibitors of KRAS and CDK4 depends on the RB family proteins and p27

**Fig. 6:**
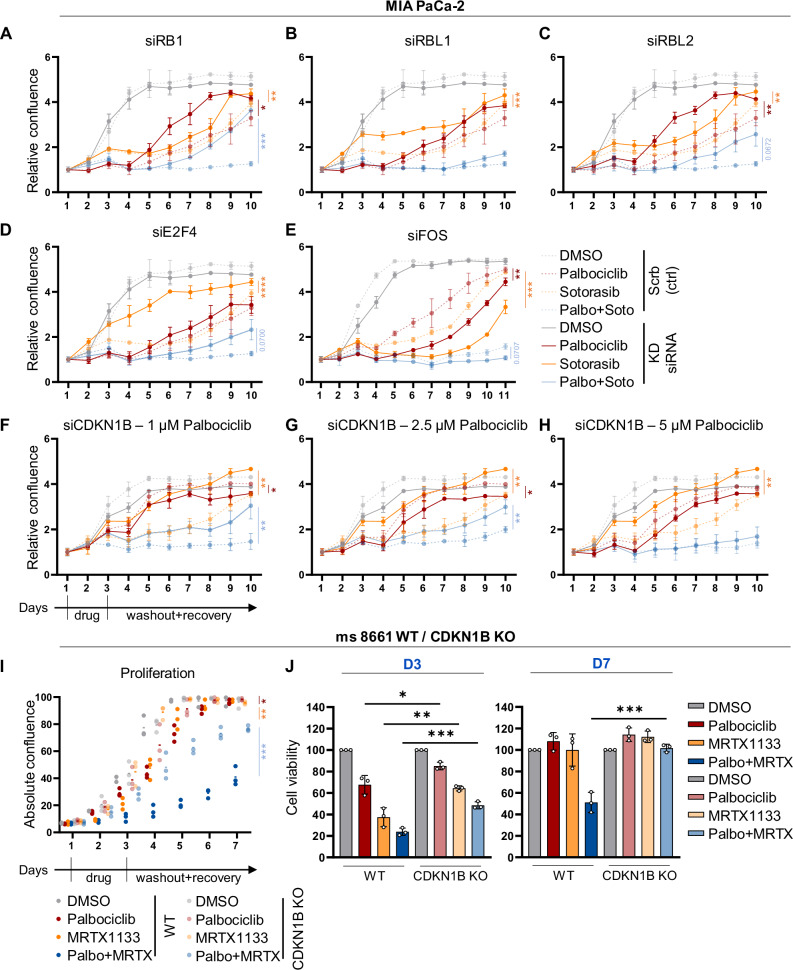
Growth suppression by inhibitors of KRAS and CDK4 depends on the RB family proteins and p27. **A** Proliferation of MIA PaCa-2 cells measured by automated transmission microscopy (Celigo®). Cells were reverse transfected by siRNAs to RB1 (**A**), RBL1 (**B**), RBL2 (**C**), E2F4 (**D**), or FOS (**E**); (scrb = ctrl siRNA). On day 1 after transfection, the cells were treated with DMSO, 10 µM (5 µM for (**E**)) Palbociclib, 5 µM Sotorasib or the combination, for 48 h, followed by 7 days of recovery in normal medium. Means of three technical replicates ±SD. **F** MIA PaCa-2 cells were transfected by siRNAs to deplete CDKN1B (**F**–**H**) or control siRNA (scrb) during seeding. On day 1, the cells were treated with 1, 2.5, or 5 µM Palbociclib, with or without 5 µM Sotorasib, 48 h, followed by seven days of recovery without drugs. Three technical replicates, means ± SD. **I** Proliferation of 8661 cells (murine PDAC) wild type (WT) or CDKN1B knock-out (KO) lines (n = 3 clones, 3 technical replicates each). All cells were treated and observed as in (**A**), with the following specifics: 1 µM Palbociclib, 0.1 µM MRTX1133 or the combination. **J** Cell viability of 8661 WT and CDKN1B KO cells evaluated at D3 and D7 corresponding to (**I**). Statistical analyses: **A**–**J** unpaired t-test (**A**–**I** of AUC); ns not significant, *p ≤ 0.05, **p ≤ 0.01, ***p ≤ 0.001, ****p ≤ 0.0001. Complete statistics in Supplementary Fig. 6A.

To investigate the mechanistic role of RB family proteins and p27 in the response to the drugs, we performed knockdown experiments by transfection of MIA PaCa-2 cells with siRNA. Depletion of RB1 significantly restored cell proliferation despite treatment with the drug combination (Fig. 6A and Supplementary Fig. 6A). While RBL1 knockdown merely attenuated the response to Sotorasib (Fig. 6B), RBL2 depletion yielded some recovery of cells that were treated with the combination (Fig. 6C). E2F4 depletion significantly accelerated cell recovery from Sotorasib treatment (Fig. 6D) but only weakly rescued the cells from combined treatment. FOS depletion significantly diminished the proliferation of cells upon treatment with Palbociclib or Sotorasib (Fig. 6E). With CDKN1B knockdown, Sotorasib-treated cells resumed proliferation rapidly, whereas the response to Palbociclib monotherapy was only slightly affected (Fig. 6F–H). Remarkably, however, CDKN1B depletion allowed significant recovery upon combination treatment, especially when using low doses of Palbociclib. All knockdowns were verified by immunoblot and/or quantitative RT-PCR (Supplementary Fig. 6B–D). To further corroborate these results, we deleted CDKN1B in the murine PDAC cell line 8661 (knockout, KO; Supplementary Fig. 6E). Cells with CDKN1B KO were less susceptible to either KRAS or CDK4 inhibition, compared to parental wild type (WT) cells. Most notably, CDKN1B KO cells recovered from the drug combination almost completely, while WT cells continued to display decreased proliferation and viability (Fig. 6I, J and Supplementary Fig. 6F, G). We conclude that the RB family and CDKN1B/p27 are key mediators of the drug synergy between Palbociclib and Sotorasib or MRTX1133.

Since p27 has a crucial role in mediating the drug synergy, and since p27 is predominantly inhibiting CDK2 [33], we hypothesized that Sotorasib or MRTX1133 can be replaced by a CDK2 inhibitor when combined with the CDK4 inhibitor. Indeed, Palbociclib along with the CDK2 inhibitor Tagtociclib synergistically reduced the proliferation of MIA PaCa-2, 51T-2D, and AsPC-1 cells (Supplementary Fig. 6H–J). Furthermore, cell viability was compromised by this combination, and the Bliss synergy score confirmed the treatment to be additive to synergistic. Taken together, these observations argue that p27 acts as an endogenous cell cycle inhibitor to mediate the synergy of CDK4- and KRAS-inhibitors. Of note, however, CDK2 inhibitors cannot be expected to act specifically on tumor cells and thus are associated with toxicities [34, 35], whereas KRAS inhibitors can be tailored to act exclusively on tumor-associated KRAS mutants. Thus, in conclusion of our results, the combination of CDK4- and KRAS-inhibitors represents a promising strategy for treating KRAS-mutant cancers.

### In an orthotopic syngeneic mouse model of PDAC, Palbociclib does not enhance the therapeutic efficacy of MRTX1133

**Fig. 7:**
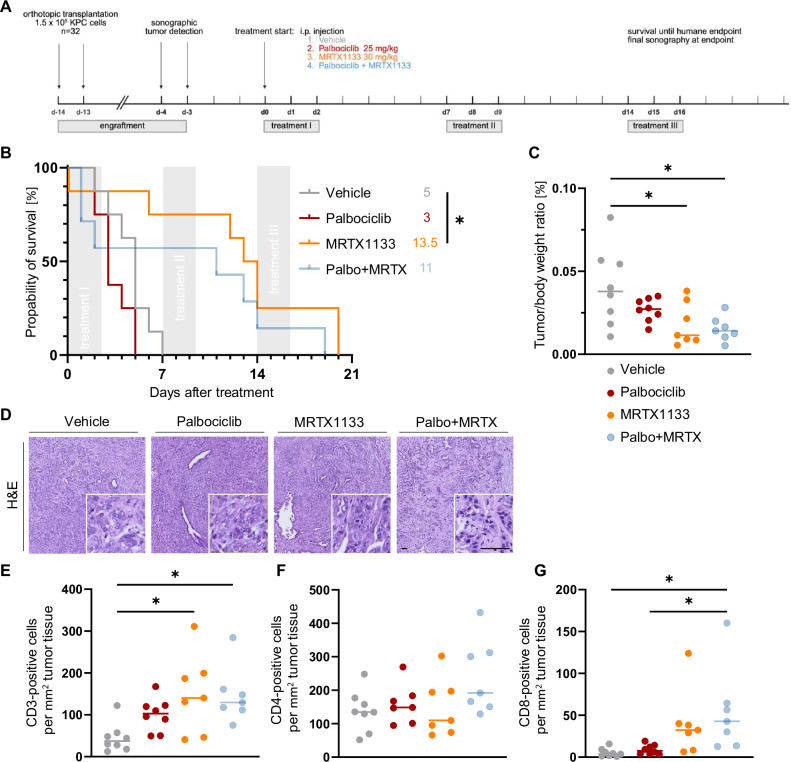
CDK4/6 inhibition does not further enhance the efficacy of KRAS^G12D^ inhibition in an immunocompetent orthotopic PDAC model. **A** In vivo treatment scheme. C57BL/6J mice were orthotopically transplanted with 1.5 × 10^5^ KPC cells. After tumor detection *via* sonography, mice were injected i.p. with (1) vehicle control, (2) Palbociclib 25 mg/kg, (3) MRTX1133 30 mg/kg and (4) Palbociclib + MRTX1133 on three consecutive days followed by a 4-days treatment break until humane endpoint. **B** Kaplan–Meier plot comparing overall survival since treatment initiation of the differently treated groups including the median survival. **C** Comparison of tumor to body weight ratio between the different treatment groups. **D** Representative images of H&E-stained tumor slices across the four treatment groups. Scale bar 50 µm. **E** Quantification of CD3 (**E**), CD4 (**F**), and CD8 (**G**) positive cells per tumor tissue. Statistical analyses: **B** Log-rank (Mantel-Cox) test followed by Bonferroni correction for multiple comparison was used for Kaplan–Meier survival plots; **C**, **E**, **F**, **G** one-way ANOVA followed by Tukey’s multiple comparison; ns not significant, *p ≤ 0.05, **p ≤ 0.01, ***p ≤ 0.001, ****p ≤ 0.0001.

To evaluate the potential of combined KRAS and CDK4 inhibition in an immunocompetent in vivo setting, we employed an orthotopic transplantation model using PDAC cells derived from a genetically engineered *LSL-KrasG12D/+; LSL-Trp53R172H/+; Pdx-1-Cre* (KPC) mouse model [36]. KPC cells were orthotopically injected into the pancreas of syngeneic *C57BL/6J* wild-type mice. Following intrapancreatic tumor detection via ultrasound, mice were randomized into four treatment groups, and Palbociclib and MRTX113 were administered intraperitoneally (i.p.) as monotherapy and in combination (n = 8 per group; Fig. 7A).

Unexpectedly, in several animals, treatment with Palbociclib as single or combination therapy led to a rapid loss of body weight and signs of pain, necessitating early euthanasia (Supplementary Fig. 7A). In contrast, MRTX1133 monotherapy did not affect the body weight; however, its administration caused signs of discomfort or pain shortly after injection (Supplementary Fig. 7B), prompting co-administration of analgesics. Furthermore, mice treated with MRTX1133 developed skin defects around the injection site, which was not observed for animals receiving Palbociclib single treatment or vehicle control. Remaining animals were sacrificed upon reaching defined endpoints (cf. Methods).

Consistent with previous reports [37, 38], MRTX1133 monotherapy significantly prolonged overall survival (13.5 vs. 5 days upon treatment initiation, Fig. 7B). In contrast, Palbociclib was neither able to improve survival on its own, nor did it enhance the therapeutic effect of MRTX1133 in the combination group. Compared to vehicle-treated mice, tumor weights at necropsy were significantly reduced by MRTX1133, both alone and in combination (Fig. 7C). Notably, comparison of tumor volumes, as detected by ultrasound before treatment initiation and immediately prior to animal sacrifice (conducted in 4–5 mice per group), indicated that the decrease in tumor weight upon MRTX1133 treatment was indeed due to the therapeutic efficacy of the drug and was not biased by smaller tumor volumes at treatment initiation (Supplementary Fig. 7C, D). Additional application of Palbociclib had no further impact on tumor weight. Signs of tumor re-growth in MRTX1133- or MRTX1133/Palbociclib-treated mice, potentially indicative for the onset of therapy-induced adaptation processes, occurred upon three weeks of drug administration (Supplementary Fig. 7E).

H&E staining confirmed PDAC morphology of harvested tumors in all four treatment groups, without noticeable changes in tumor cell differentiation or composition across the cohort (Fig. 7D and Supplementary Fig. 7F–H). However, immunohistochemistry staining for CD31/Pecam-1, an endothelial cell marker, revealed a trend towards increased vascularization in Palbociclib- and combination-treated tumors (Supplementary Fig. 7F, I); this at least suggests that Palbociclib might stimulate angiogenesis in the tumors, in agreement with previously reported findings [39]. Analysis of the tumor samples at the study endpoint showed no detectable changes in the expression of key proliferation (Ki67) and apoptosis (cleaved caspase-3, CC3) markers (Supplementary Fig. 7F, J, K), in contrast to our cell culture results. Importantly, and consistent with our in vitro findings illustrating prolonged maintenance of RB1 hypophosphorylation upon combined KRAS and CDK4 inhibition (Fig. 5), immunohistochemical analysis revealed RB1 hypophosphorylation (pRB1) only in combination-treated, but not in MRTX1133- or Palbociclib single-treated animals (Supplementary Fig. 7F, L). Of note, the two outliers with high pRB1 levels in the combination group were harvested more than 3 days after the last drug administration (Supplementary Fig. 7M), suggesting that re-phosphorylation occurred at this time point.

Since previous reports [37, 38] linked MRTX1133 treatment to an accelerated host immune response, and based on the enrichment of interferon-signatures upon KRASi detected in our transcriptome analysis (Supplementary Fig. 4E, F), we determined the relative spleen weights at necropsy. Interestingly, MRTX1133 treatment alone, but not the combination therapy, significantly increased the spleen-to-body weight ratio (Supplementary Fig. 7N). While no difference in the ratio of red pulp compared to the whole spleen was observed (Supplementary Fig. 7O, P), an increase in extramedullary hematopoiesis (EMH) was detected in MRTX1133-treated animals, which may explain the spleen phenotype (Supplementary Fig. 7Q).

To characterize the T-cell response in the tumors, we assessed tumor infiltration by CD3-positive cells in each treatment group. MRTX1133- and combination-treated tumors revealed significantly more extensive T-cell infiltration compared to vehicle-treated animals (Fig. 7E and Supplementary Fig. 7R). While CD4-positive cells displayed a trend of elevation in the combination-treated animals, CD8-positive cells were significantly elevated in the combination-treated tumors compared to vehicle-treated animals (Fig. 7F, G and Supplementary Fig. 7R).

Interferon gamma staining in tumors did not show detectable differences (Supplementary Fig. 7R, S). Moreover, the proportion of CD3-, CD4-, or CD8-positive T-cells within tumor-associated lymph nodes was similar in all treatment groups (Supplementary Fig. 7T–W). Finally, the quantification of a panel of cytokines revealed that CXCL13, ICAM-1, and TIMP-1 were present in all four treatment groups, in plasma and/or tumor samples, without detectable quantitative differences (Supplementary Fig. 7X, Y).

Taken together, these results argue that neither Palbociclib nor MRTX1133 compromise the host immune response, but that they might modulate the TME by inducing angiogenesis (Palbociclib) and T-cell infiltration (MRTX1133).

### Single cell RNA sequencing revealed pronounced cell cycle arrest signatures upon treatment with MRTX1133, and angiogenesis in Palbociclib-treated tumors

**Fig. 8:**
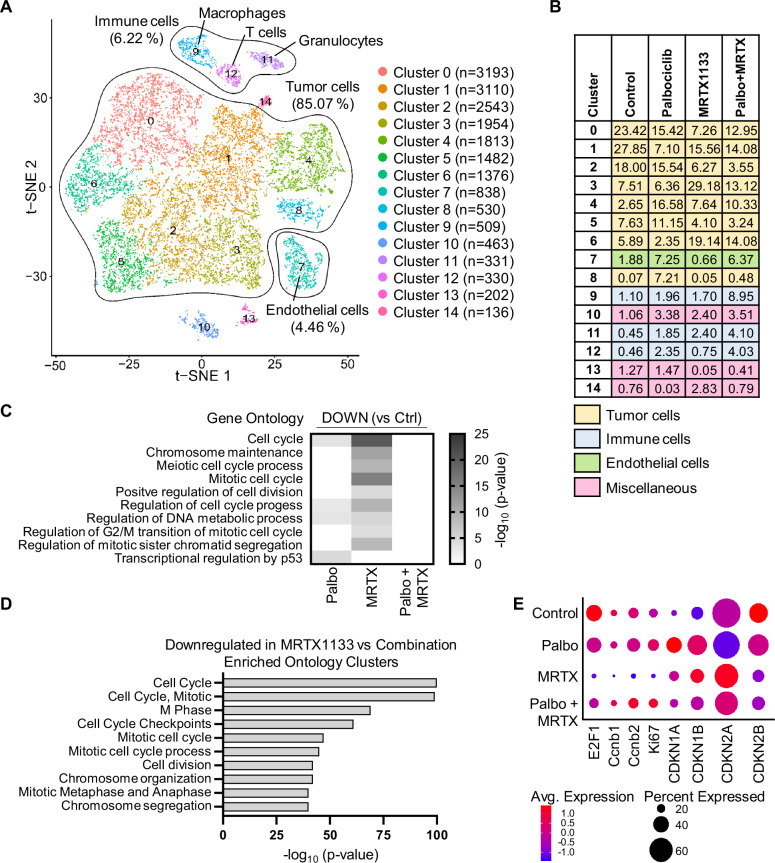
Single cell RNA sequencing revealed pronounced cell cycle arrest signatures upon treatment with MRTX1133, and angiogenesis in Palbociclib-treated tumors. **A** tSNE visualization of clusters derived from single cell RNA sequencing of the mouse tumors (vehicle, Palbociclib, MRTX1133, or combination). n = 2 animals per treatment group. Different colors represent different clusters with cell numbers per cluster mentioned in brackets. Annotated cell types were identified using SingleCellNet and the expression of key markers. **B** Distribution of the cells of each sample in the different clusters. The numbers represent the percentage of cells per sample in each cluster, while the colors of the rows represent the identity of the cells within the cluster. **C** Analysis of gene ontology of differentially expressed genes of Palbociclib, MRTX1133 or combination vs vehicle in tumor cells (clusters 0–6 and 8) via metascape (downregulated, top 1000 genes). Only genes with |log2fold| ≥ 0.25 and adjusted p-value (padj.) < 0.05 were included in the analysis. Supplementary Table 3 contains DE genes of all 15 clusters and of tumor clusters 0–6 and 8 separately. **D** Analysis performed as in (**C**) for the comparison of MRTX1133 vs combination (1980 genes). **E** Dot plot of average expression and percent expressed for tumor cells (clusters 0–6 and 8) for specific genes of the four treatment groups.

While the animals in our study largely benefited from MRTX1133 monotherapy, Palbociclib did not enhance their survival, neither alone nor in combination (Fig. 7B), in contrast with our cell culture analyses. In order to understand the molecular details of this response, we performed single cell RNA sequencing of tumors obtained from the four treatment groups upon necropsy (two mice per treatment group). Fifteen cell clusters were identified across the treatment groups and displayed by t-distributed stochastic neighbor embedding (tSNE). Clusters 0–6 and 8 correspond to the malignant cells expressing *Krt19*, comprising 85% of all cells. Clusters 9, 11, and 12 correspond to immune-mediator cells expressing *Ptprc*, representing 6% of all cells analyzed (Fig. 8A and Supplementary Fig. 8A–C). The tSNE plots split per condition visualized more similarities between vehicle and Palbociclib, and between MRTX1133 and the combination (Supplementary Fig. 8D). scRNA sequencing displayed the largest percentage of T-cells in response to the drug combination. This further supports our immunostaining results, which also revealed more CD3-positive cells for combination-treated and MRTX1133-treated animals, compared to vehicle-treated mice (Fig. 7E). Overall, the combination treatment group comprised the largest fraction of immune cells (T-cells, macrophages and granulocytes) with 17% compared to 2% in vehicle-, 6% in Palbociclib- and 5% in MRTX1133-treated animals. Of note, combination-treated tumors were characterized by a higher percentage of macrophages (8.95%) compared to all other treatment groups (Fig. 8B). Taken together, these results suggest that macrophages, T-cells and other immune cells accumulate in the tumors upon combination treatment.

Furthermore, endothelial cells (cluster 7) were enriched in the groups receiving Palbociclib or the drug combination. These treatment groups were characterized by significantly higher expression of the vascularization marker *CD31/Pecam1* (Supplementary Fig. 8E). This further supports the increase of CD31/Pecam1 observed by immunohistochemistry in the animals receiving Palbociclib (Supplementary Fig. 7I). These results argue in favor of Palbociclib-driven angiogenesis, in agreement with a previous report [39].

In the malignant cell clusters, the gene ontology of differentially expressed genes was analyzed (Fig. 8C and Supplementary Fig. 8F). While MRTX1133-treated tumors, compared to vehicle, revealed significant downregulation of genes associated with cell cycle progression, Palbociclib-treated tumors displayed only small changes of these genes, and, most notably, combination-treated tumors did not reveal any significant changes in this gene ontology. Furthermore, the comparison of MRTX1133 versus combination clearly demonstrated a significant downregulation of similar gene ontologies such as cell cycle, M Phase or cell division (Fig. 8D). In line with these results was the reduced average expression of *E2F1* and its target genes *Ccnb1* and *Ccnb2* in MRTX1133-treated tumors (Fig. 8E). While MRTX1133 treatment upregulated *Cdkn1b*, this was not observed in the combination-treated tumors, in contrast to our in vitro findings. These observations strongly suggest that MRTX1133 induces a profound cell cycle arrest, while Palbociclib antagonizes these effects in the investigated model system. This is in contrast to our observations in cell culture systems. One possible explanation might consist in Palbociclib-driven tumor angiogenesis, leading to increased supply with oxygen and nutrients and perhaps enabling faster tumor cell proliferation.

## Discussion

Our results reveal profound drug synergy against cancer cells when combining inhibitors of KRAS and CDK4 for the activation of an RB1-dependent restriction point of the cell cycle. These observations were particularly striking when quantifying viable cells a few days after drug removal. Thus, the combination of the drugs confers a degree of sustainability that neither of the antagonists achieves on its own. Considering that such durable impact is particularly desirable in the context of cancer, the observations strongly argue to develop these or similar combinations for therapy. While RB1 hypophosphorylation upon combination treatment was also observed in vivo, it notably did not translate into a detectable therapeutic benefit of Palbociclib in animals when combined with the KRAS inhibitor MRTX1133.

Studies performed before direct KRAS inhibitors became available support the concept of targeting Ras signaling along with CDK4. For instance, combining inhibitors of MEK/MAP2K and CDK4 also revealed synergy [20, 22], as did inhibitors of ERK/MAPK3 and CDK4 [21, 24, 25]. Moreover, CDK4 inhibitors might improve the efficacy of drugs targeting BRAF, a kinase downstream of KRAS but upstream of MEK/ERK, e.g., in the context of malignant melanoma [40, 41]. Similar combinations were also reported for colorectal cancer [42, 43]. We have compared KRAS inhibitors with MEK inhibitors in PDAC cells (Supplementary Fig. 1P, Q) and observed significant but less pronounced cooperation of the latter with CDK4 inhibition. Within the limits of comparing compounds with different pharmacological specificities, this argues that direct inhibitors of KRAS act more broadly, by diminishing most if not all signaling pathways that would otherwise be driven by KRAS—besides RAF-MEK-ERK, these also include PI3K-AKT and RAL [44, 45]. Perhaps, the simultaneous reduction in several pathway activities provides more opportunities for synergy with CDK4 inhibition. Along this line, a recent report suggested the combination of KRAS- and CDK4/6 inhibitors to eliminate “persister” cells in tumors that arise from monotherapy with KRAS inhibitors [46].

Our results also suggest that RB1, through its ability to suppress E2F1 activity, is required for the efficacy of the drug combination. In addition, p27/CDKN1B acts as its mediator. These findings argue that tumors with RB1 deletions do not represent good candidates for treatment with CDK4 inhibitors. Mutations or deletions of *CDKN1B* are rare in cancer to begin with [47]. On the other hand, we anticipate that the efficacy of KRAS inhibition could be overcome by activating mutations in the downstream signaling components, e.g., the RAF kinases. However, neither RB1 nor RAF kinases are frequent subjects to mutation in PDAC [48, 49], which is in support of taking the combined inhibition of KRAS and CDK4 to clinical applications for treating PDAC. However, the risk remains that potential therapies with inhibitors of KRAS and CDK4 may evoke the accumulation of such mutations in cancer cells or might induce alternative non-genetic adaptation mechanisms, thus priming therapy resistance. On top of mutations within downstream effectors, mutations within the targets themselves or within factors that act in parallel to the targets may also occur [50], e.g., mutations in the KRAS paralogues HRAS and NRAS, or in CDK4. On the other hand, since each of the two drugs can interfere with cell growth individually through different pathways, this may counteract the onset of therapy-induced genetic or non-genetic adaptation mechanisms.

Prior studies have demonstrated that KRAS inhibition can provoke a robust inflammatory response and promote T cell infiltration into tumors, thereby contributing to its anti-tumor efficacy [37, 38, 51]. Consequently, both MEK and KRAS inhibition sensitize tumors to immune checkpoint blockade [35, 36, 39, 45]. Consistent with these findings, we observed that KRAS inhibition, alone or along with Palbociclib, resulted in elevated T-cell infiltration in our animal model.

Based on our in vivo model, we hypothesize that Palbociclib’s effect on the TME might antagonize the effect of MRTX1133, since Palbociclib-treated tumors are better supplied with oxygen and nutrients. Next to an increase of vascularization, elevated levels of macrophages might also promote angiogenesis [52]. The combination of the MEK inhibitor Trametinib and the CDK4 inhibitor Palbociclib was reported to induce a senescence-associated secretory phenotype (SASP), along with pro-angiogenic factors that remodel the endothelium and facilitate CD8 + T cell infiltration into the TME of murine PDACs [39]. The increased supply of blood in the combination-treated animals may explain why a strong cell cycle arrest was seen in MRTX1133-only-treated tumors, but not in combination-treated tumors. In summary, changes in the TME might increase the resilience of tumors receiving Palbociclib.

To overcome this limitation, effective in vivo application of the KRAS-CDK4 inhibitor combination may require an additional anti-VEGF strategy [53] to prevent vascularization of tumors. Moreover, the combination therapy might benefit from dose optimization or improvement of pharmacokinetics and treatment schedules. Toxicities of CDK4/6 inhibitors such as Palbociclib were previously observed in patients, too [54, 55]. The use of specific CDK4 inhibitors (without targeting CDK6) [56] may limit the toxicities that we observed in the present study.

Early-phase clinical trials are already exploring the combination of KRAS and CDK4/6 inhibitors, within broader multi-agent regimens (e.g., NCT04185883). While our findings underscore the challenges of translating this strategy into effective in vivo therapies, they also highlight a path forward. Beyond pancreatic and non-small cell lung cancers, a wide spectrum of KRAS-mutant tumors—such as colorectal carcinomas and other refractory malignancies—could potentially benefit from such targeted combinations. Crucially, the success of this approach will likely depend on a suitable TME context and possibly on the avoidance of tumor-supportive vascularization.

## Materials and methods

Antibodies

**Table:** 

Reagent or resource	Source	Identifier
AKT	Cell Signaling	Cat# 9272; RRID: AB_329827
Alexa Flour 568 goat Anti-mouse	Thermo Fisher Scientific	Cat# A-11004; RRID: AB_2534072
Alexa Fluor 488 goat Anti-rabbit	Thermo Fisher Scientific	Cat# A-11034; RRID: AB_2576217
Alexa Fluor 555 goat Anti-rat	Thermo Fisher Scientific	Cat# A-21434; RRID: AB_2535855
BrdU / CldU	Abcam	Cat# ab6326; RRID: AB_305426
Cleaved Caspase 3 (CC3)	Cell Signaling	Cat# 9661; RRID: AB_2341188
CD3	Abcam	Cat# ab16669; RRID: AB_443425
CD31	Abcam	Cat# ab28364; RRID: AB_726362
CD4	Abcam	Cat# ab183685; RRID: AB_2686917
CD8α	Cell Signaling	Cat# 85336; RRID: AB_2800052
cMYC	Cell Signaling	Cat# 18583; RRID: AB_2895543
Cyclin D1	Abcam	Cat# ab134175; RRID: AB_2750906
Cyclin E1	Cell Signaling	Cat# 4129; RRID: AB_2071200
Donkey anti-mouse IgG, HRP conj.	Jackson ImmunoResearch Labs	Cat# 715-036-150; RRID: AB_2340773
Donkey anti-rabbit IgG, HRP conj.	Jackson ImmunoResearch Labs	Cat# 711-036-152; RRID: AB_2340590
E2F-1	Cell Signaling	Cat# 3742; RRID: AB_2096936
E2F4	Cell Signaling	Cat# 40291; RRID: AB_2799174
ERK1	Santa Cruz	Cat #sc-94; RRID: AB_2140110
FOS	Proteintech	Cat#66590; RRID: AB_2881950
GAPDH	Abcam	Cat# ab8245; RRID: AB_2107448
HSC70	Santa Cruz	Cat# sc-7298; RRID: AB_627761
Interferon gamma	Invitrogen	Cat # MM700; RRID: AB_223537
KRAS	Sigma-Aldrich	Cat# WH0003845M1; RRID: AB_1842235
Ki67	Abcam	Cat# ab15580; RRID: AB_443209
p27	BD Biosciences	Cat# 554069; RRID: AB_395225
PARP1	Cell Signaling	Cat#9542; RRID: AB_2160739
phospho-AKT (Ser473) (D9E)	Cell Signaling	Cat# 4060; RRID: AB_2315049
phospho-ERK1/2 (Thr202/Thr204)	Cell Signaling	Cat# 4370; RRID: AB_2315112
phospho-RB1 (Ser807/811)	Cell Signaling	Cat# 9308; RRID: AB_331472
phospho-RB1 (T373)	Abcam	Cat# ab52975; RRID: AB_2177344
phospho-RB (Ser807/811)	CellSignaling	Cat# 8516; RRID: AB_11178658
RB1 (4H1)	Cell Signaling	Cat# 9309; RRID: AB_823629
RBL1	Cell Signaling	Cat# 89798; RRID: AB_2800144
RBL2	Cell Signaling	Cat# 13610; RRID: AB_2798274
β-actin	Abcam	Cat# ab6276; RRID: AB_2223210

Chemicals, peptides, and recombinant proteins

**Table:** 

Reagent or resource	Source	Identifier
5-Chloro-2′-deoxyuridine	Sigma-Aldrich	Cat# C6891
Abemaciclib	Selleckchem	Cat# S5716
AC220	Medchem Express	Cat# HY-13001
Bovine Serum Albumin	Sigma	Cat# A4503
25x cOmplete Protease Inhibitor Cocktail	Roche	Cat #11697498001
CytosealTM 60	Epredia	Cat# 83124
DAPI	Sigma-Aldrich	Cat# D9542
DMSO	Geyer	Cat# A3672
DNase I	Zymo Research	Cat# E1010
dNTP Set, 100 mM Solutions	Thermo Fisher Scientific	Cat# R0182
Hoechst33342	Thermo Fisher Scientific	Cat# H3570
Hydrogen peroxide 33% w/v	PanReac AppliChem	Cat# 131077.1211
Isofluran CP	CP Pharma	Cat# 1214
M-MuLV Reverse Transcriptase	New England Biolabs	Cat# M0253
Matrigel® Growth Factor Reduced (GFR) Basement Membrane Matrix, Phenol-Red Free, LDEV-free	Corning	Cat# 356231
MRTX1133	Medchem Express	Cat# HY-134813
Nuclease-Free Water	Life Technologies	Cat# AM9939
Palbociclib PD 0332991 isethionate	Sigma-Aldrich	Cat #PZO199
Pefabloc	Carl Roth	Cat# A154
Pepstatin A	VWR	Cat# A2205
PMSF	Sigma-Aldrich	Cat# 78830
Propidium Iodide	Geyer	Cat# P4864
Rimadyl	Zoetis	N/A
RMC-7977	Medchem Express	Cat# HY-156498
RNase A	Quiagen	Cat# 19101
SCH772984	Selleckchem	Cat# S7101
Sodium fluoride	Sigma-Aldrich	Cat#S1504
Sodium orthovanadate	Sigma-Aldrich	Cat##S6508
Sotorasib	Medchem Express	Cat #HY-114277
SYBR Green	Invitrogen	Cat# S7567
Tagtociclib	ChemieTek	Cat# CT-PF0710
Taq Polymerase	Primetech	Cat# 1800
Trametinib	Selleckchem	Cat# S2673
Trypan blue solution 0.4%	Roth	Cat# 1680.1
TRIzol™ reagent	Life Technologies	Cat# 15596018
Tween-20	Sigma-Aldrich	Cat# P9416

Critical commercial assays

**Table:** 

Reagent or resource	Source	Identifier
Alt-R® CRISPR-Cas9 tracrRNA	Integrated DNA Technologies	Cat# 0000882514
Alt-R™ S.p. Cas9-GFP V3	Integrated DNA Technologies	Cat # 10008100
Annexin V-FITC Apoptosis Detection Kit	Thermo Fisher Scientific	Cat# ICT-9124
CellTiter-Glo® Luminescent Cell Viability Assay	Promega	Cat# G7571
Immobilon Western HRP Substrate	Geyer/Millipore	Cat# WBKLS0500
ImmPACT® DAB Substrate kit, Peroxidase	Vector Laboratories	Cat# SK-4105
Lipofectamine 3000 transfection reagent	Thermo Fisher Scientific	Cat# L3000015
Masson’s Trichrome Stain Kit	Polysciences Europe	Cat# 25088
Tumor Dissociation Kit mouse	Miltenyi Biotec	Cat# 130-096-730
Pierce BCA Protein Assay Kit	Thermo Fisher Scientific	Cat# 23227
Proteome Profiler Mouse Cytokine Array Kit, Panel A	Biotechne	Cat# ARY006
Senescence beta-Galactosidase Cell Staining	Cell Signaling	Cat# 9860
SuperSignal West Femto Maximum Sensitivity Substrate	Thermo Fisher Scientific	Cat# 34095
Transfection reagent TransIT®-LT1	Mirus	Cat #731-0027
VECTASTAIN® ABC kit, Peroxidase (Rabbit IgG)	Vector Laboratories	Cat# PK-4001

Experimental models: cell lines

**Table:** 

Reagent or resource	Source	Identifier
Human: 51T-2D	Provided by Günter Schneider, CRU5002, Göttingen	N/A
Human: AsPC-1	ATCC	Cat# CRL-1682; RRID: CVCL_0152
Human: Capan-1	ATCC	Cat# HTB-79; RRID: CVCL_0237
Human: Capan-2	ATCC	Cat# HTB-80; RRID: CVCL_0026
Human: GöCDX52	Provided by Elisabeth Hessman, CRU5002, Göttingen	N/A
Human: GöCDX53	Provided by Elisabeth Hessman, CRU5002, Göttingen	N/A
Human: GöCDX7	Provided by Elisabeth Hessman, CRU5002, Göttingen	N/A
Human: MIA PaCa-2	ATCC	Cat# CRL-1420; RRID: CVCL_0428
Human: MOLM13	DSMZ	Cat# ACC 554; RRID:CVCL_2119
Human: NCl-H2122	ATCC	Cat# CRL-5985; RRID: CVCL_1531
Human: NCl-H358	ATCC	Cat# CRL-5807; RRID: CVCL_1559
Mouse: 8661 CDKN1B knock-out	Provided by Günter Schneider, CRU5002, Göttingen	N/A
Mouse: 8661 WT	Provided by Günter Schneider, CRU5002, Göttingen	N/A
Mouse: KPC	Provided by Elisabeth Hessmann, CRU5002, Göttingen	N/A

*N/A* not available

Experimental models: organoids

**Table:** 

Reagent or resource	Source	Identifier
PDO-51T (case: KFO-TM047)	Provided by CRU5002, Göttingen	N/A

Experimental models: animals

**Table:** 

Reagent or resource	Source	Identifier
C57BL/6J	Breeding by central animal facility, UMG	N/A

Oligonucleotides

**Table:** 

Reagent or resource	Source	Identifier
siRNA CDKN1B #1	Thermo Fisher Scientific	Cat# s2838
siRNA CDKN1B #2	Thermo Fisher Scientific	Cat# s2837
siRNA CDKN1B #3	Thermo Fisher Scientific	Cat# s2839
siRNA E2F4 #1	Thermo Fisher Scientific	Cat# s4414
siRNA E2F4 #2	Thermo Fisher Scientific	Cat# s4416
siRNA E2F4 #3	Thermo Fisher Scientific	Cat# s223455
siRNA FOS #1	Thermo Fisher Scientific	Cat# s5339
siRNA FOS #2	Thermo Fisher Scientific	Cat# s5340
siRNA FOS #3	Thermo Fisher Scientific	Cat# s5341
siRNA RB1 #1	Thermo Fisher Scientific	Cat# s522
siRNA RB1 #2	Thermo Fisher Scientific	Cat# s523
siRNA RB1 #3	Thermo Fisher Scientific	Cat# s524
siRNA RBL1 #1	Thermo Fisher Scientific	Cat# s11853
siRNA RBL1 #2	Thermo Fisher Scientific	Cat# s11854
siRNA RBL1 #3	Thermo Fisher Scientific	Cat# s11852
siRNA RBL2 #1	Thermo Fisher Scientific	Cat# s11856
siRNA RBL2 #2	Thermo Fisher Scientific	Cat# s11855
siRNA RBL2 #3	Thermo Fisher Scientific	Cat# s11857
siRNA scrambled #1	Thermo Fisher Scientific	Cat# 4390844
siRNA scrambled #2	Thermo Fisher Scientific	Cat# 4390847
primer	5′ → 3′	
*36B4* forward (M. Wienken)	GATTGGCTACCCAACTGTTG	This study
*36B4* reverse (M. Wienken)	CAGGGGCAGCAGCCACAAA	This study
*BIRC* forward (S. Gerber)	TGACGACCCCATAGAGGAAC	This study
*BIRC5* reverse (S. Gerber)	TTCTCCGCAGTTTCCTCAAATTC	This study
*BRCA1* forward (S.Gerber)	TGATCAAGGAACCTGTCTCCAC	This study
*BRCA1* reverse (S. Gerber)	ACTTTCTTGTAGGCTCCTTTTGG	This study
*CCNB1* forward (S. Gerber)	CTGAGACAACTTGAGGAAGAGC	This study
*CCNB1* reverse (S. Gerber)	ACATGGTCTCCTGCAACAAC	This study
*CCND1* forward	CCGAGGAGCTGCTGCAAATG	This study
*CCND1* reverse	CACAGAGGGCAACGAAGGTC	This study
*CCNE1* forward	TCCAGGAAGAGGAAGGCAAACG	This study
*CCNE1* reverse	ATTGTCCCAAGGCTGGCTCC	This study
*CDK1* forward	GGAATAATAAGCCGGGATCTACC	This study
*CDK1* reverse	TAGGAACCCCTTCCTCTTCACT	This study
*CDK2* forward	CATCTTTGCTGAGATGGTGACTC	This study
*CDK2* reverse	CACTTGGGGAAACTTGGCTTG	This study
*CDK4* forward	CTCTGAAGCCGACCAGTTG	This study
*CDK4* reverse	TAAAAGTCAGCATTTCCAGCAGC	This study
*CDKN1B* forward qRT-PCR	CCTGCAACCGACGATTCTT	This study
*CDKN1B* reverse qRT-PCR	TCGAGCTGTTTACGTTTGACG	This study
*CDKN1B* forward PCR (X. Fang)	ATCCCTTGTCCCGACTCACT	This study
*CDKN1B* reverse PCR (X. Fang)	GACCCAATTAAAGGCACCGC	This study
*E2F1* forward	CGGTGTCGTCGACCTGAACT	This study
*E2F1* reverse	AGGACGTTGGTGATGTCATAGATG	This study
*FOS* forward	AAGGAGAATCCGAAGGGAAAGG	This study
*FOS* reverse	TAGTTGGTCTGTCTCCGCTTG	This study
*PLK4* forward (S. Gerber)	CCCTTCTCAGAAAATGAAGCTCG	This study
*PLK4* reverse (S. Gerber)	GAAAGTGTGAGGTCCCGGTG	This study
*RBL2* forward	CTGCCGAGTCGCCCA	This study
*RLB2* reverse	TGAAGATCATTTCCCTCCAGCG	This study

Software and algorithms

**Table:** 

Reagent or resource	Source	Identifier
BioRender	BioRender	https://www.biorender.com/
CeligoTM Software version2	Nexcelom	N/A
FIJI version 1.54f	Image J	N/A
Galaxy GWDG	GWDG	http://galaxy.gwdg.de
Guava Express Pro/Cyto Soft 5.3	Millipore	N/A
Image Lab version 5.2.1	BioRad	N/A
PRISM version 9 + 10	GraphPad	N/A
QuPath version 0.5.1	Bankhead et al.^62^	https://qupath.github.io/
R environment version 4.1.2	www.r-project.com	N/A
R package: “Colorbrewer” version 1.1-2	Neuwirt^57^	N/A
R package: “ggplots2” version 3.3.6	Wickham et al.^58^	N/A
R package: “qglots” version 3.1.3	Warnes et al.^59^	N/A
R Studio version 2022.07.02 + 552	www.rstudio.com	N/A
Slideview VS200	Olympus	VS200
Synergyfinder	synergyfinder.fimm.fi	N/A
Vevo 2100 Imaging System	VisualSonics	N/A
ZEN Software version 3.6	Zeiss	N/A

### Cell culture, cell lines, and inhibitors

The human cell lines AsPC-1, NCl-H358, HCl-H2122 and Capan-2 were maintained in Roswell Park Memorial Institute (RPMI 1640, 42401042, Life Technologies) supplemented with 10% Fetal Calf Serum (FCS, ACSM0190, Anprotec), 50 U/mL Penicillin, 50 μg/mL Streptomycin (15140122, GIBCO) and 2 mM Glutamine (25030024, GIBCO). Capan-1 and MOLM13 were cultivated as described above with 20% FCS. Human MIA PaCa-2 cells were maintained in Dulbecco’s modified Eagle’s medium (DMEM, 31600091, Thermo Fisher Scientific) supplemented with 10% FCS, 50 U/mL Penicillin, 50 μg/mL Streptomycin and 2 mM Glutamine. Murine KPC (LSL-KrasG12D/+; LSL-Trp53R172H/+; Pdx-1-Cre) cells were maintained in DMEM similar to MIA PaCa-2 cells with additional 1% non-essential amino acids (11140050, Thermo Fisher Scientific). Murine 8661 cells were cultivated in DMEM GlutaMAX (61965059, GIBCO) with 10% FCS and 2 mM Glutamine. The patient-derived cell line 51 T-2D was established from a patient (case: KFO-TM047) from the CRU5002 cohort [27]. 51T-2D cells were maintained in RPMI 1640 supplemented with 10% FCS and 2 mM Glutamine. Further patient-derived xenograft lines GöCDX7, GöCDX52, and GöCDX53 (cases: KFO-TM004, KFO-TM056, KFO-TM057) were maintained in a 3:1 ratio of keratinocyte-serum free medium with 25 mg bovine pituitary extract (BPE) and 2.5 µg epidermal growth factor (EGF) (17005042, GIBCO) and RPMI 1640 supplemented with 10% FCS. All cell lines were cultured at 37 °C, 5% CO_2_ and regularly tested to ensure the absence of Mycoplasma contaminations. Patient-derived organoids (PDO-51T) were cultivated as described [27].

For drug treatment, we used Abemaciclib (S5716, Selleckchem), AC220 (HY-13001, MedChem Express), MRTX1133 (HY-134813-5, Hycultec GmbH), Palbociclib (PZ0199-5MG, Sigma-Aldich), RMC-7977 (HY-156498, MedChem Express), SCH772984 (S7101, Selleckchem), Sotorasib (HY-114277, MedChem Express), Tagtociclib (CT-PF0710, ChemieTek) or Trametinitb (S2673, Selleckchem). DNA synthesis was quantified by 5-chloro-2-deoxyuridine (CldU) incorporation (25 µM; Sigma-Aldrich).

Transient transfections were carried out using Lipofectamine 3000 (L3000008, Thermo Fisher Scientific). siRNAs were transfected at a final concentration of 10 nM as smart pool targeting CDKN1B (s2837, s2838, s2839, Thermo Fisher Scientific), E2F4 (s4414, s4416, s223455, Thermo Fisher Scientific), FOS (s5339, s5340, s5341), RB1 (s552, s523, s524, Thermo Fischer Scientific), RBL1 (s11852, s11853, s11854, Thermo Fischer Scientific), RBL2 (s11855, s11856, s11857, Thermo Fisher Scientific); scrambled siRNA was used as control (s4390844, s4390847, Thermo Fisher Scientific).

Targeted deletion (knock-out, KO) of CDKN1B was performed in murine 8661 cells. CDKN1B was deleted by CRISPR/Cas9 using gRNA (5’-GCGGATGGACGCCAGACAAG-3’ and 5’-CAAACGTGAGAGTGTCTAAC-3’), Alt-R® CRISPR-Cas9 tracrRNA (0000882514, Integrated DNA Technologies) and Alt-R™ S.p. Cas9-GFP V3 (10008100, Integrated DNA Technologies) through lipofection (TransIT®-LT1, Mirus, Lot No. 12094394). Forty-eight hours after lipofection, the media were changed, and green fluorescent protein (GFP) signals were checked through the microscope. Twenty-four hours after the media change, single-cell cloning was conducted by seeding WT and CDKN1B KO cells into 96-well plates (0.75 cells per well). Fifteen hours after seeding, the wells were manually checked for the existence of single cells in each well. Wells with more than one cell were excluded from further handling. After 2–3 weeks of cultivation, clones were expanded and analyzed. To select clones containing the desired deletion, dot western blot was performed to exclude clones with p27 protein expression. In addition, PCR was performed using the forward (5’-ATCCCTTGTCCCGACTCACT-3′) and reverse primers (5’-GACCCAATTAAAGGCACCGC-3′). The targeting of CDKN1B was confirmed by Sanger sequencing of the PCR product.

### Cell viability assays

The Cell Titer-Glo® Luminescent Cell Viability Assay (G7571, Promega) was used to determine the viability of adherent cells. Viability was plotted using R environment and R Studio (www.r-project.com, www.r-studio.com, version 4.2.1) with the following packages “Colorbrewer” ([57] version 1.1-2), “ggplots2” ([58] version 3.3.6) and “gplots” ([59] version 3.1.3). The synergy of two drugs was calculated via synergyfinder.fimm.fi [60] using the BLISS score and interpreted as follows. Bliss score >10: synergy; -10 – 10: additive mechanism; <-10: antagonism.

### Cell proliferation assays

Adherent cells were seeded into 96-well plates (3606, Corning) and treated with drugs the following day for 48 h. Then, the cells were incubated with their respective media. Cell confluence was measured every 24 h by bright field microscopy using the Celigo^TM^ Cytometer (Nexcelom), for up to 11 days. Confluence was determined with the corresponding Celigo^TM^ software (Nexcelom, software version 2.0) by analyzing the percentage of surface occupied by adherent cells over the total surface. Suspension cells (MOLM13) were seeded in medium with treatment on day 0. After 48 h cells were washed and centrifuged and reseeded in fresh medium without treatment. Cells were counted every day using trypan blue exclusion to quantify the cell number and viability.

### Immunoblot analyses

Whole cell lysates were prepared in RIPA lysis buffer, i.e., 20 mM Tris-HCl pH 7.5, 150 mM NaCl, 10 mM EDTA, 1% Triton-X 100, 1% sodium deoxycholate, 0.1% SDS, 2 M urea, and protease inhibitors (pepstatin, leupeptin hemisulfate, aprotinin, AppliChem) and sonicated for 10 min. The BCA protein assay kit (23227, Thermo Fisher Scientific) was used to determine the total protein concentration. Samples were boiled for 5 min at 95 °C in Laemmli buffer, and equal amounts of proteins were separated by SDS-PAGE, followed by transfer to a nitrocellulose membrane. The membrane was blocked with 5% (w/v) non-fat milk (Roth) in TBS supplemented with 0.1% Tween-20 (AppliChem) for 2 h. Primary antibody incubation was performed at 4 °C overnight. Peroxidase (HRP)-conjugated secondary antibodies (Jackson ImmunoResearch Labs) were applied to the membranes, and proteins were detected using Immobilon Western Substrate (WBKLS0500, Millipore) or Super Signal West Femto Maximum Sensitivity Substrate (34095, Thermo Fisher Scientific). All uncropped immunoblots are available in a separate file as Supplementary material.

### Reverse transcription and real-time quantitative PCR (qRT-PCR)

Total RNA was extracted from cells using TRIzol® (15596018, Life Technologies). Two hundred microliter Chloroform was added per 1000 µL TRIzol. For phase separation, samples were centrifuged, and the aqueous phase was used to precipitate the RNA with 800 µL isopropanol. Two washing steps with 75% ethanol were performed before the RNA was resuspended in nuclease-free water (AM9939, Life Technologies). The concentration was determined via spectrophotometry.

RNA was reverse-transcribed using oligo-dT and random nonamers as primers, followed by qRT-PCR analysis using SYBR Green (S7567, Invitrogen), as previously described [61]. Gene expression levels were normalized to the mRNA levels from the housekeeping gene *36B4*, and the analysis was conducted using the ΔΔCt method.

### Transcriptome analyses

Total mRNA of 51T-2D and AsPC-1 cells was obtained as described above. Sequencing of mRNA (≥400 ng) was performed on Illumina NovaSeq 6000 by Novogene (read length: paired-end 150 bp). mRNA sequencing data were processed in the Galaxy environment provided by the GWDG (Gesellschaft für Wissenschafltiche Datenverarbeitung mbH Göttingen, https://galaxy.gwdg.de/). In short, a quality check was performed using FastQC (version 0.69). Afterwards, the first 11 nucleotides of each read were trimmed (Trim, version 0.0.2). Fastq files were aligned to the GRCh38 reference genome using HISAT2 (version 2.0.5.2). Read counts for each sample and gene were acquired via featureCounts (version 1.6.3+galaxy2). Finally, DeSeq2 (version 2.11.40.6+galaxy1) was used to perform differential gene expression analysis and to obtain normalized counts (Supplementary Tables 1 and 2). To analyze differentially regulated genes, only genes with a cut-off of |log2fold| ≥ 0.6, adjusted p-value (padj.) < 0.05, and baseMean ≥15 were included. Gene Set Enrichment Analysis of hallmark gene sets (GSEA, version 4.1.0, https://www.gsea-msigdb.org/gsea/msigdb/index.jsp) was used to identify impaired pathways. Differentially expressed genes were correlated with the Molecular Signature Database (MsigDB) using the Enrichr platform (https://maayanlab.cloud/Enrichr/). Z-Score heat maps of differentially regulated genes were obtained from Morpheus (https://software.broadinstitute.org/morpheus/). Raw data are available at GEO GSE301146 and GSE301148.

### Nucleoside analog incorporation assay

Cells were seeded in 96-well plates (3606, Corning) and treated as indicated. Twenty-five microliter CldU was applied to cells for 2 h at 37 °C, 5% CO_2_ before fixation with 4% PFA (Sigma-Aldrich) in PBS for 20 min. Cells were washed 4x in PBS. Permeabilization and denaturation were performed using 0.5% Triton-X-100/2.5 M HCl for 1 h. Blocking was carried out with 3% BSA (w/v) + 0.1% Tween-20 in PBS for 1 h. This was followed by incubation with a primary antibody (α-BrdU, ab6326, Abcam) overnight at 4 °C. After three washing steps with blocking solution, the cells were incubated with the secondary antibody (A-21434, Thermo Fisher Scientific), together with 4′,6-diamidino-2-phenylindole (DAPI, D9542, Sigma-Aldrich) for 1 h at RT. Images were acquired using the Cell Discoverer 7® (Zeiss) with 20x magnification. ZEN software (Zeiss, version 3.6) was used to quantify the CldU signal over the DAPI signal.

### Immunofluorescence staining of organoids

Organoids were seeded in 96-well plates (3606, Corning) and treated as indicated. On day 7, the organoids were incubated with Hoechst 33342 (100 μg/mL, H3570, Thermo Fisher Scientific) and Propidium Iodide (PI, P4864, Geyer). Images were acquired using the Cell Discoverer 7® (Zeiss) with ×20 magnification. Brightfield and immunofluorencence images were acquired as Z-stacks to depict complete organoids.

### Annexin V assay

Cell death was analyzed by performing Annexin V staining. The Annexin V-FITC Apoptosis Detection Kit (ICT-9124, Biozol) was used, with the addition of PI (10 μg/mL, P4864, Geyer), Hoechst 33342 (100 μg/mL, H3570, Thermo Fisher Scientific). To quantify cell death, cells were seeded in low density and stained at the indicated time points. Analysis was performed using the Celigo^TM^ Cytometer (Nexcelom) with the corresponding Celigo^TM^ software (Nexcelom, software version 2.0).

### Flow cytometry

To determine the DNA content (cell cycle distribution) in response to different treatment conditions, cells were harvested by trypsin treatment at the indicated time points. After centrifugation, cell pellets were washed in PBS and fixed in 75% EtOH overnight. For the analysis of the DNA content, cells were first washed with PBS twice and then subjected to 0.5 mg/mL RNase A (19101, Quiagen) treatment for 30 min at 37 °C before staining by propidium iodide (1 μg/mL, P4864, Geyer) for 15 min. Cell cycle profiles were obtained using the Guava® EasyCyte plus flow cytometer. Analysis was performed using the Guava Software Cyto Soft.

### Cell senescence determination by senescence-associated beta-galactosidase (SAB) staining

Cell senescence was analyzed using the Senescence beta-Galactosidase Cell Staining kit (9860, Cell Signaling). First, the cells were washed and fixed. The staining solution was prepared freshly for every use and was applied overnight at 37 °C. Imaging was performed using a Zeiss Axiocam 503 color microscope. Finally, FIJI was used to analyze ten images per condition with the script imagej_cell_count_jython (https://github.com/lquenti/imagej_cell_count_jython).

### Syngenic orthotopic mouse model of PDAC

Forty microliter cell suspension containing 1.5 × 10^5^ KrasG12D;Trp53R172H/+ (KPC) cells in culture medium and 50% matrigel was injected into the pancreatic tail of C57BL/6J mice aged 4 to 6 months (n = 32) as described previously [36]. Small animal ultrasound scan (Vevo 2100 Imaging System, VisualSonics) under Isoflurane (1214, CP Pharma) anesthesia was performed to detect tumor engraftment. Upon successful tumor detection, mice were assigned to treatment groups considering equal distribution of sex, physical constitution, age and tumor volume. Vehicle-control, Palbociclib (25 mg/kg, PZO199, Sigma-Aldrich) and/or MRTX1133 (30 mg/kg, HY-134813, Medchem Express) were administered i.p. once a day within weekly cycles of three consecutive treatment days followed by 4 days recovery until humane endpoint. If possible, a final small animal ultrasound scan was performed. The physical condition of all mice was assessed daily and Rimadyl (5 mg/kg body weight; Zoetis) was administered after treatment with Palbociclib and/or MRTX1133 if indicated by signs of discomfort. Humane endpoints include weight loss >11% >24 h or >20%, signs of severe or treatment-independent pain and tumor size >1 cm. Spleen, pancreas and tumor were harvested and weighted after euthanasia. Plasma samples were harvested using EDTA and centrifuged for 20 min 2000g. Animal experiments were conducted in accordance with the animal welfare regulations and were approved by the Niedersächsisches Landesamt für Verbraucherschutz und Lebensmittelsicherheit (24-00539).

### Immunohistochemical staining

H&E staining was conducted by the Department of Pathology, UMG, Prof. Dr. P. Ströbel. Spleen H&E stainings were analyzed by pathologist M. Bleyer, DPZ, for the degree of extramedullary hematopoesis (EMH). The red pulp of spleens was assessed for differences in composition of myeloid and erythroid precursors as well as megakaryocytes to grade the degree of EMH. For IHC, 4 µm-thick slices from formalin-fixed and paraffin-embedded (FFPE) tissue blocks were deparaffinized, and rehydrated and the antigens were retrieved by boiling in citric acid pH 6.0. The endogenous peroxidase was inhibited by 3% hydrogen peroxide (131077.1211, PanReac AppliChem) for 10 min and unspecific binding sites were blocked by 10% BSA in PBS 0.1% Tween-20 (P9416, Sigma-Aldrich). Incubation of the primary antibodies (anti-CD3, 1:200, ab16669, abcam,; anti-CD31, 1:100, ab28364,abcam; anti-CD4, 1:400, ab183685,abcam; anti-CD8, 1:200, 85336, Cell Signaling; anti-IFNgamma, 1:400, MM700, Invitrogen; anti-Ki67, 1:600, ab15580, abcam; anti-phospo-RB, 1:500, 8516, Cell Signaling) was conducted over night at +4 °C in 1% BSA (A4503, Sigma) in PBS 0.1% Tween-20 (P9416, Sigma-Aldrich) followed by incubation of the biotinylated secondary antibody (anti-rabbit IgG, PK-4001, Vector Laboratories) at room temperature for 1 h. The Avidin-Biotin complex from the VECTASTAIN^®^ ABC kit (PK-4001, Vector Laboratories) was incubated 30 min to allow assembling prior to binding to the secondary antibody at RT for 1 h. Detected antigens were visualized using the ImmPACT^®^ DAB Substrate kit (SK-4105, Vector Laboratoris) according to the manufacturer’s instructions followed by counterstaining in Hematoxylin for 5 min, dehydration and mounting in Cytoseal^TM^ 60 (83124, Epredia^TM^). Whole tissue sections were scanned in ×20 magnification by slide scanner (VS200, Olympus).

IHC staining was quantified using QuPath bioimage analysis software (version 0.5.1) [62]. Malignant regions were manually annotated, hereby physiologic pancreas, cystic lesions, large blood vessels, tissue folds and staining artefacts were excluded. Color correction, quantification and classification algorithm was trained by two representative image areas of each treatment group. Cells were counted using the “positive cell detection” tool after optimization with automatically estimation of stain vectors. Positive CD31 staining was calculated per tumor size using the pixel classification tool by creating a thresholder measuring only stained area.

Masson’s trichrome staining was performed using a stain kit from Polysciences Europe (25088), following the manufacturer’s guidelines. Briefly, the tissue was deparaffinized, rehydrated, and fixed with Bouin’s fixative overnight at room temperature. The next day, the tissue was stained sequentially in a working solution of Weigert’s hematoxylin, Biebrich scarlet-acid fuchsin, phosphotungstic/phosphomolybdic acid, and aniline blue. The staining was fixed with 1% acetic acid prior to dehydration and mounting, as previously described. Stained slides were scanned and tumors were annotated using QuPath. Further analysis was performed with ImageJ using color threshold followed by particle analysis.

### Cytokine array

Cytokines and chemokines were analyzed using the Proteome Profiler Mouse Cytokine Array Kit (ARY006, Biotechne). The analysis was performed using either 100 µl plasma (pooled from two animals/treatment group) or 400 µg tumor tissue (pooled from two animals/treatment group). The tumors were homogenized in lysis buffer, i.e., 50 mM HEPES, 150 mM NaCl, 1 mM EGTA, 10% Glycerol, 1% Triton X-100, 100 mM NaF, 10 mM Na4P207, containing freshly added protease inhibitors 1× cOmplete Protease Inhibitor Cocktail (#11697498001, Roche), 100 µM NaF (S1504, Sigma), 10 µM sodium orthovanadate (#S6508, Sigma Aldrich) and 1 mM PMSF (#78830, Sigmal Aldrich) and centrifuged at 13000 x *g* for 20 min. The supernatant was used to determine the protein concentration using the BCA protein assay kit (23227, Thermo Fisher Scientific). Equal amounts of total protein were used for array analysis. Two samples per group were selected based on their day of sacrifice (vehicle/Palbociclib: day 4 and 5; MRTX1133/combination: day 13 and 14).

### Single cell RNA sequencing

Two samples per group were selected based on their day of sacrifice (vehicle/Palbociclib: day 4 and 5; MRTX1133: day 12 and 14; combination: day 11 and 14). Single cells were isolated from mouse tumors using the Tumor Dissociation Kit mouse (130-096-730, Miltenyi Biotec) and the program 37C_m_TDK_2. After the dissociation, DNase A (200 U/mL, E1010, Zymo Research) treatment (as suggested in step 11 of the manufacturers protocol) was performed. Single cell suspensions were counted and stained prior to library preparation starting with 17k nuclei (vitality 60–78%). For library construction, the PIPseq™ T2 3′ Single Cell RNA Kit v4.0 was used, enabling the profiling of up to 5000 individual cells/sample. This protocol employs particle-templated instant partitions (PIPs) to encapsulate cells with barcoded beads, facilitating efficient mRNA capture and cDNA synthesis and using IMI, a sample-specific index/barcode, usually introduced during library preparation, to distinguish different samples pooled in the same sequencing run. The sequencing was performed on the NovaSeq6000, using an S1 flow cell and generating 500 million reads/sample. The sequencing depth was corresponding to a minimum of 20,000 reads per cell.

Raw base call (BCL) files were converted to FASTQ format and processed using bcl2fastq (version 2.20.0.422). The Illumina BaseSpace DRAGEN Single Cell RNA Pipeline (version 4.4.4) on the BaseSpace Cloud platform. The DRAGEN single-cell pipeline performed read alignment, barcode processing, UMI counting, and gene quantification. Reads were aligned to the Mus musculus (based on GENCODE mm39) provided within the DRAGEN reference package.

Barcode/MI Source were selected as Barcode/MI read and the kit section switched to Illumina Single Cell 3′ RNA Library Kit. Cell barcodes and UMIs were automatically extracted and filtered according to DRAGEN single-cell quality control criteria by using “Ratio” method to create maximum number of cells with expected noise which calculated by estimation the count threshold as max (Te, Tm). Tm is 10% of the count seen in the cell at the 10th percentile of the expected cells (defined as 10,000). Te is 50% of the count seen in the least abundant expected cell. To give the flexibility to adjust quality filtering in the analysis section. The rest of the parameters were kept to their default values. Barcodes, features and matrix tables were processed further to be analyzed.

Further analysis took place using Seurat (version 5.3.01) following standard filtering parameters. Following dimensionality reduction, clustering took place using a resolution of 0.5. Differentially expressed genes were called using the FindMarkers function and plots were generated using the TSNEPlot, FeaturePlot, and VlnPlot functions of Seurat [63]. Specific clusters were subsetted using the subset function of Seurat according to the expression of the relevant markers (PTPRC for immune clusters and KRT19 for tumor clusters) and cell type classification using the SingleCellNet package [64]. Training took place with the provided TM10x mouse atlas training dataset subsetted to include PDAC relevant cells where alveolar macrophages, bladder cells, bladder urothelial cells, endocardial cells, kidney-related cells, luminal epithelial cell of mammary gland and lung endothelial cells were removed. Violin plots were then generated using the sc_violinClass function of SingleCellNet2 with the dLevel option to define separation by condition or seurat_clusters. Statistical significance was determined by the Wilcoxon Rank Sum test. Differentially regulated genes were filtered with a threshold of |log2fold| ≥ 0.25 and adjusted p-value (padj.) < 0.05 (Supplementray Table 3). Metascape was used to analyze gene ontologies (https://metascape.org/gp/index.html#/main/step1) [65]. Raw data are available at GEO repository GSE311611.

### Statistics

Statistical testing was performed using GraphPad Prism Software (Version 10). Unpaired t-tests were used for statistical analysis of area-under-curve (AUC, cell proliferation analysis), ordinary one-way ANOVA followed by Turkey’s multiple comparison was used for RT-PCRs, immunofluorescence staining, immunohistochemistry staining and organoid viability analysis. Log-rank (Mantel-Cox) test followed by Bonferroni correction for multiple comparison was used for Kaplan–Meier survival plots. The Kruskal–Wallis Test and post hoc Dunn’s multiple comparison was used for EMH grading. Wilcoxon Rank Sum test served to analyze violin plots derived from single cell RNA sequencing. Ns, not significant; ∗p-value ≤ 0.05; ∗∗p-value ≤ 0.01; ∗∗∗p-value ≤ 0.001; ∗∗∗∗p-value < 0.0001. All statistical details can be found in the figure legends.

### Ethics approval and consent to participate

All methods were performed in accordance with the relevant guidelines and regulations. Animal experiments were conducted in accordance with the animal welfare regulations and were approved by the Niedersächsisches Landesamt für Verbraucherschutz und Lebensmittelsicherheit (24-00539). Humane endpoints include weight loss >11% >24 h or >20%, signs of severe or treatment-independent pain and tumor size >1 cm. Informed consent for the generation and characterization of PDAC models was obtained from all patients in accordance with the regulations of the ethical committee of the University Medical Center Göttingen (ethical approval number: 11/5/17).

## Supplementary information

Supplemental Figures and Legends

Supplementary Table 1

Supplementary Table 2

Supplementary Table 3

Uncropped Western blots

Checklist

## Data Availability

The mRNA sequencing datasets generated during and analyzed during the current study are available at GEO repository (https://www.ncbi.nlm.nih.gov/geo/): GSE301146 and GSE301148. The single cell RNA sequencing dataset generated and analyzed during the current study are available at GEO repository: GSE311611.
