# Supplementary material for: Simultaneous targeting of KRAS and CDK4 synergistically induces durable growth arrest in pancreatic cancer cells
Source: Cell Death Dis. 2025 Dec 23;17(1):129. doi: 10.1038/s41419-025-08362-w (PMC12847887; doi:10.1038/s41419-025-08362-w)
Supplement: Supplementary file 1 — Supplemental Figures and Legends [file 41419_2025_8362_MOESM1_ESM.pdf]

Suppl. Figure 1

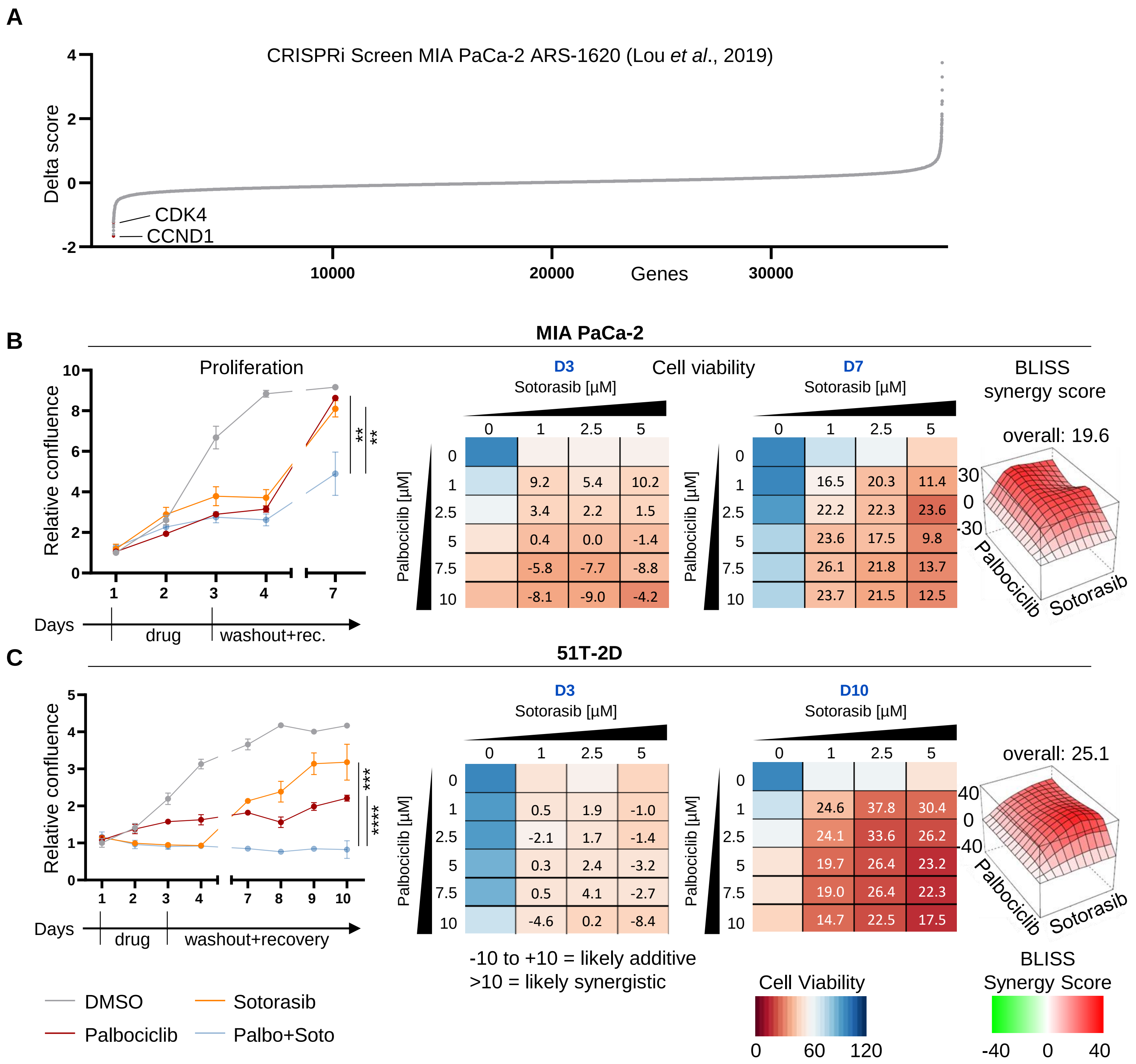

Suppl. Figure 1 continued

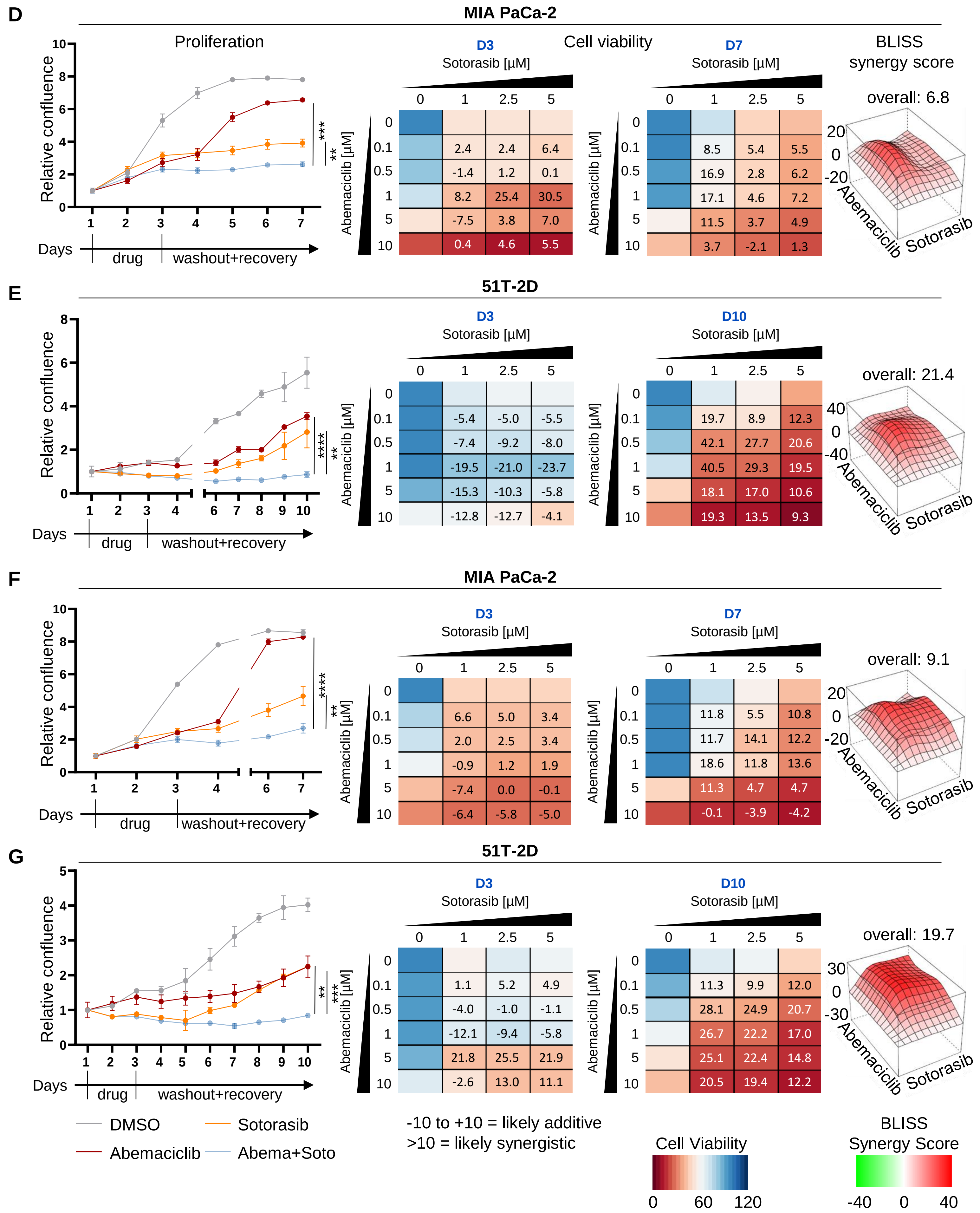

Suppl. Figure 1 continued

H

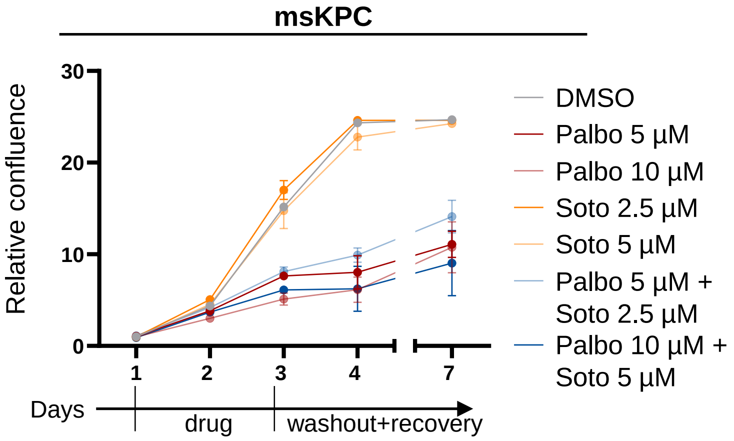

I

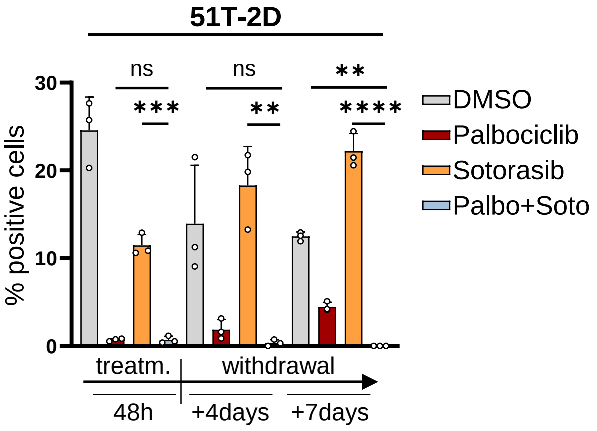

J

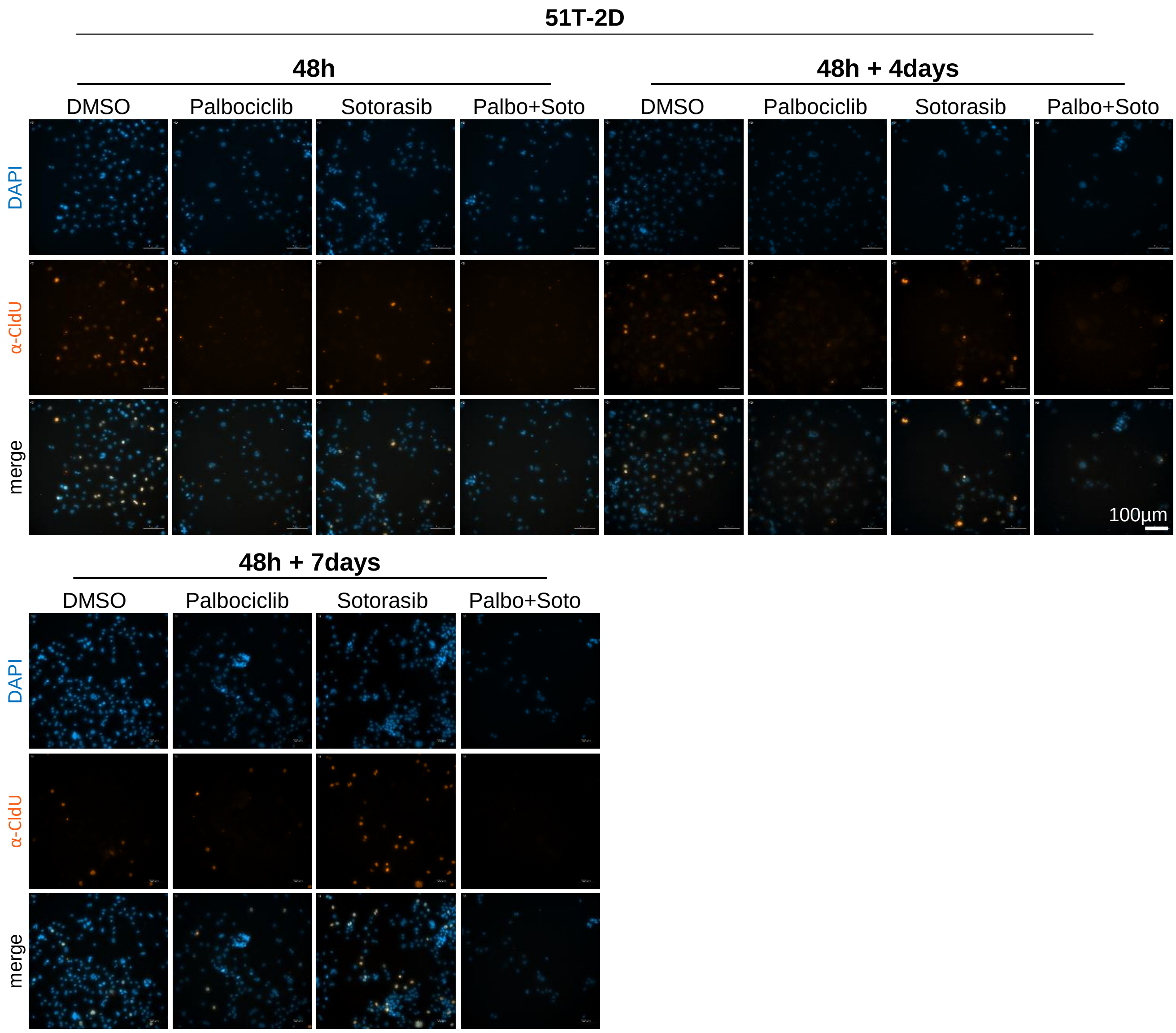

Suppl. Figure 1 continued

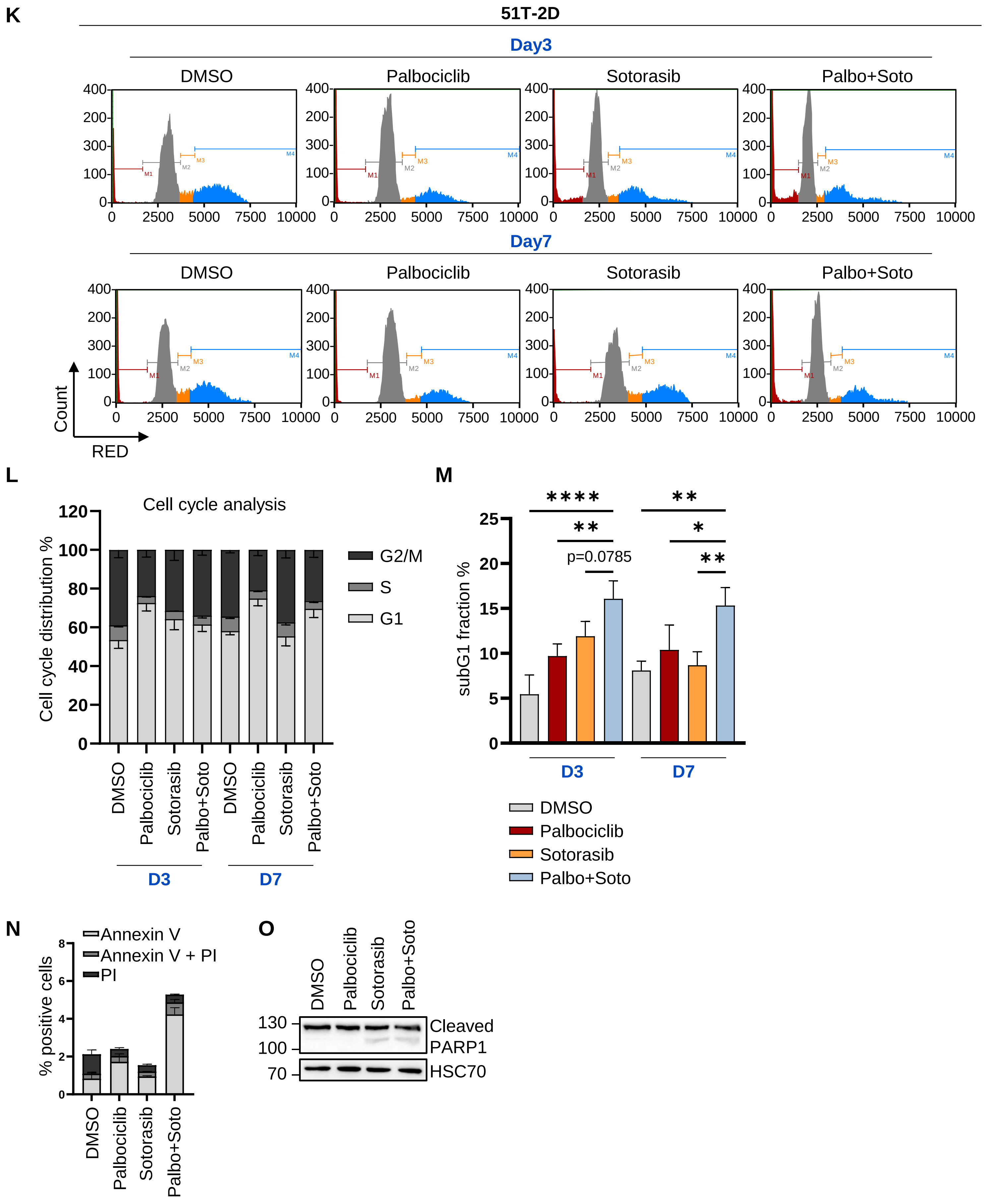

Suppl. Figure 1 continued

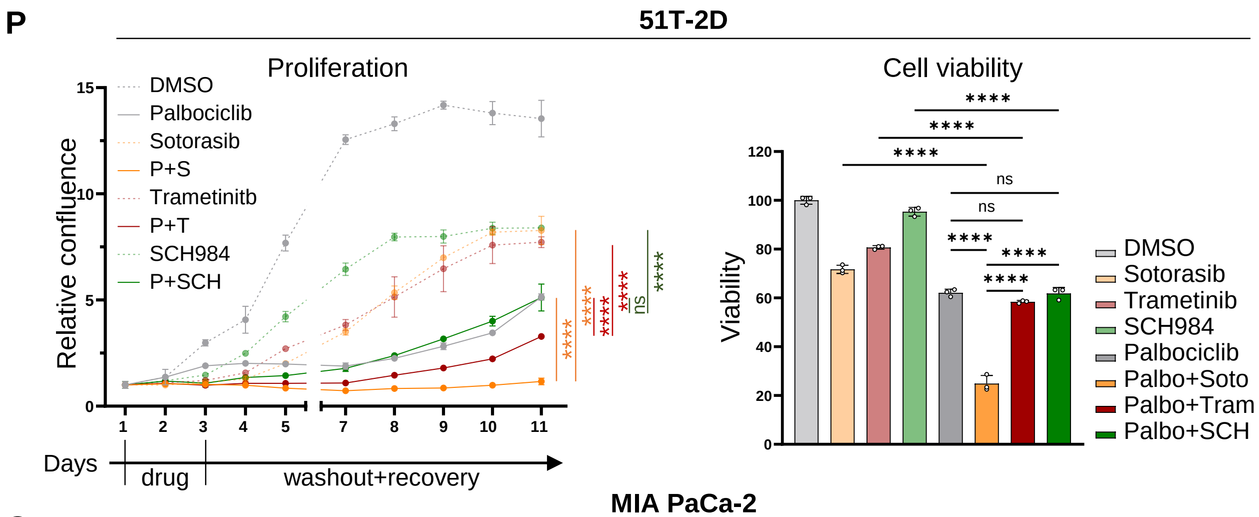

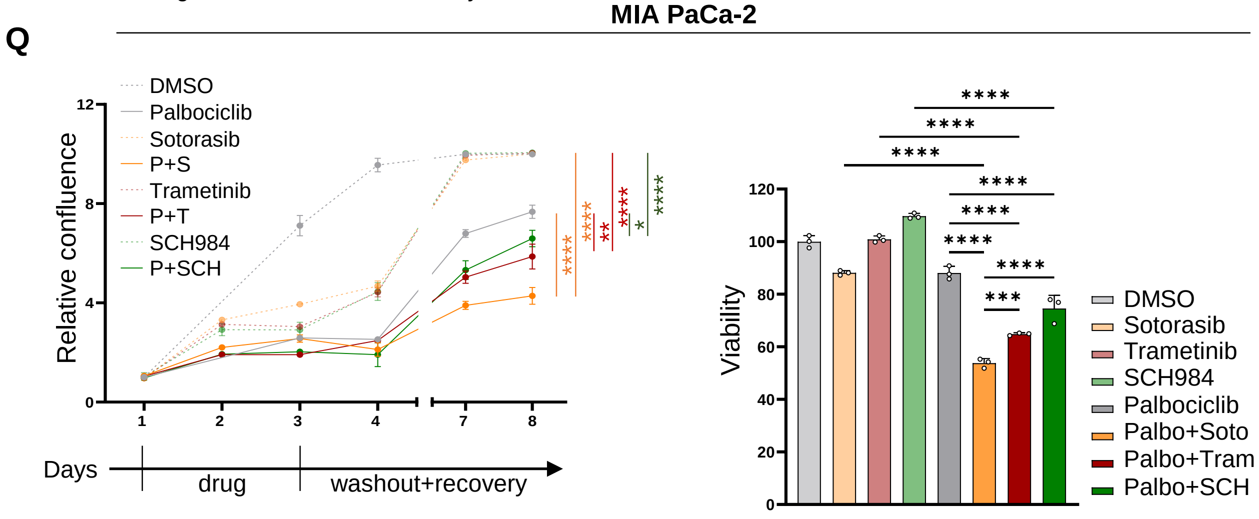

Suppl. Figure 1: The KRAS mutant G12C-targeting drug Sotorasib synergizes with Palbociclib to suppress the growth of pancreatic cancer cells and organoids, with sustainability after drug removal.

(A) CRISPRi screen of MIA PaCa-2 cells treated with 300 nM ARS-1620 in comparison to DMSO (Lou *et al.,* 2019). Analysed Delta score of Suppl. Table 4 therein. CDK4 and CCND are highlighted in red.

(B) (left) Proliferation of MIA PaCa-2 cells, measured by automated microscopy (Celigo®) upon treatment with DMSO, 5 µM Palbociclib and 2.5 µM Sotorasib or the combination for 48 h, followed by four days of recovery in normal medium. Graphs indicate the mean of three technical replicates ± SD. (right) Heat maps depict the viability of MIA PaCa-2 cells right after treatment (D3) and after recovery (D7), normalized to the DMSO control. Bliss synergy scores were determined based on cell viability at D10.

(C) 51T-2D cell proliferation was determined as in (B); 5 µM Palbociclib and 2.5 µM Sotorasib; D3 and D10 BLISS synergy map at D10.

(D) MIA PaCa-2 cells, 1 µM Abemaciclib and 5 µM Sotorasib, D3 and D7 BLISS synergy map at D7.

(E) 51T-2D cells, 0.5 µM Abemaciclib and 1 µM Sotorasib, D3 and D10.

(F) MIA PaCa-2 cells, 1 µM Abemaciclib and 5 µM Sotorasib, D3 and D7.

(G) 51T-2D cells, 0.5 µM Abemaciclib and 1 µM Sotorasib, D3 and D10.

(H) KPC cells, 5/10 µM Palbociclib and/or 2.5/5 µM Sotorasib for 48 h, followed by four days of recovery in normal medium. Means of three replicates ± SD.

(I) 51T-2D cells treated with 5 µM Palbociclib, 2.5 µM Sotorasib or both, for 48 h, with subsequent washout and further incubation for four or seven days. The cells were stained with DAPI and α-CldU to indicate DNA synthesis. CldU positive cells were quantified using the Celldiscoverer7®.

(J) Representative images to (I) are shown. Scale bar 100 µm.

(K) Representative profiles of flow cytometry with PI staining to identify DNA content and cell cycle distribution. 51T 2D cell were treated with DMSO, 5 µM Palbociclib and 2.5 µM Sotorasib or the combination for 48 h, followed by four days of recovery in normal medium.

(L) Quantification of the cell cycle distribution corresponding to (K). Data shown as mean ± SD, n=4.

(M) Quantification of the sub-G1 fraction corresponding to (K).

(N) Apoptosis quantification using the Celigo®. 51T-2D cells were as in (I) for 48 h followed by seven days of recovery in normal medium. For analysis of apoptosis cells were stained with Annexin V and PI. Quantification was carried out via the Celigo®.

(O) Immunoblot analysis of whole-cell lysates of 51T-2D. Cells were treated as in (I) for 48 h. HSC70 served as loading control.

(P) 51T-2D cells, 5 µM Palbociclib, 2.5 µM Sotorasib, 25 nM Trametinib or 0.5 µM SCH772984. Bar graphs show cell viability right after treatment (D3) and after recovery (D11), normalized to the DMSO control.

(Q) MIA PaCa-2 cells, 10 µM Palbociclib, 2.5 µM Sotorasib, 25 nM Trametinib or 0.5 µM. Cell viability as in (M) at D3 and D11.

Statistical analyses: B, C, D, E, F, G unpaired t-test; I, M, P, Q one-way ANOVA followed by Tukey's multiple comparison (B, C, D, E, F, G, P, Q: of AUC); ns: not significant, *p ≤ 0.05, **p ≤ 0.01, ***p ≤ 0.001, ****p ≤ 0.0001.

Suppl. Figure 2

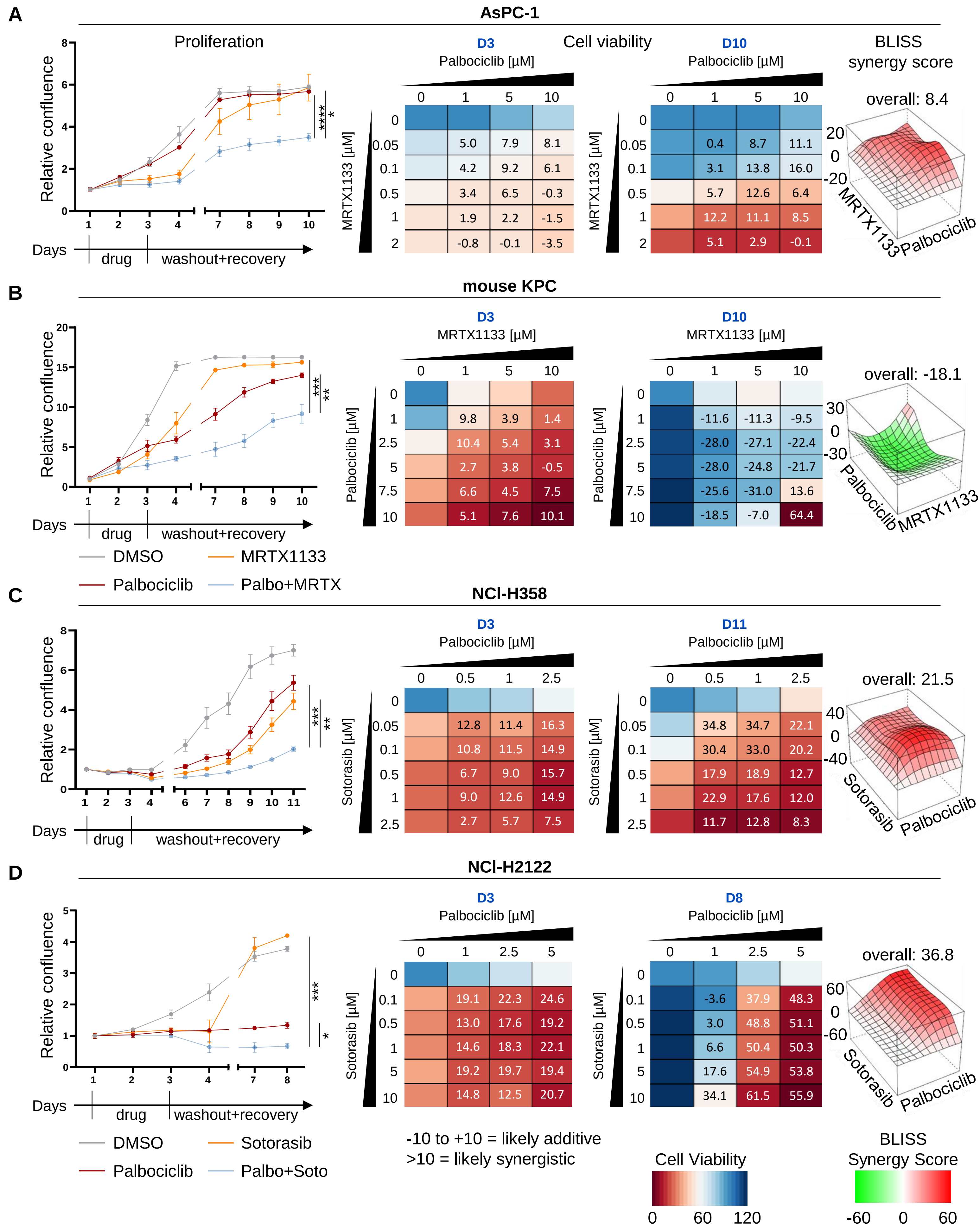

Suppl. Figure 2 continued

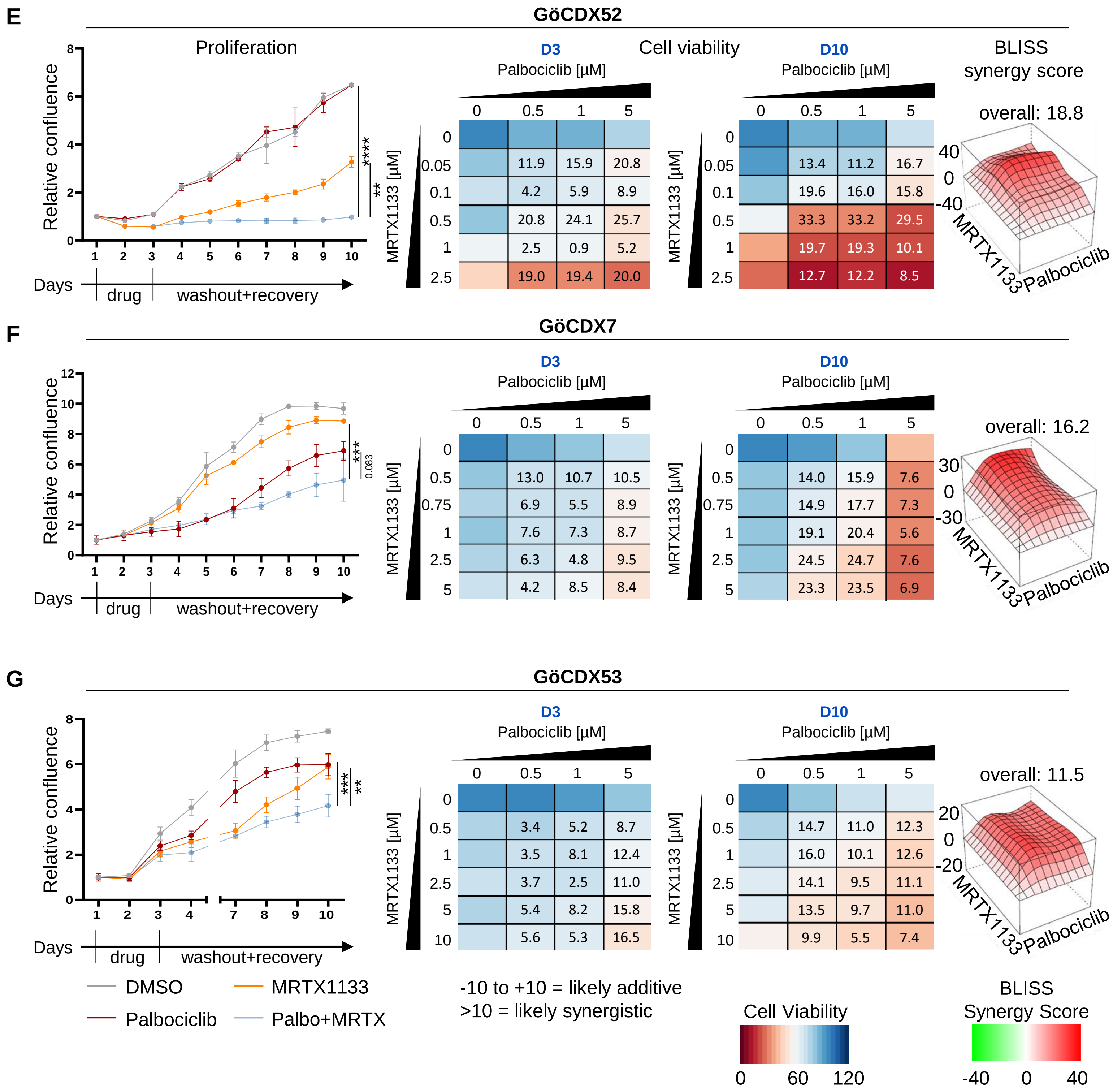

Suppl. Figure 2 continued

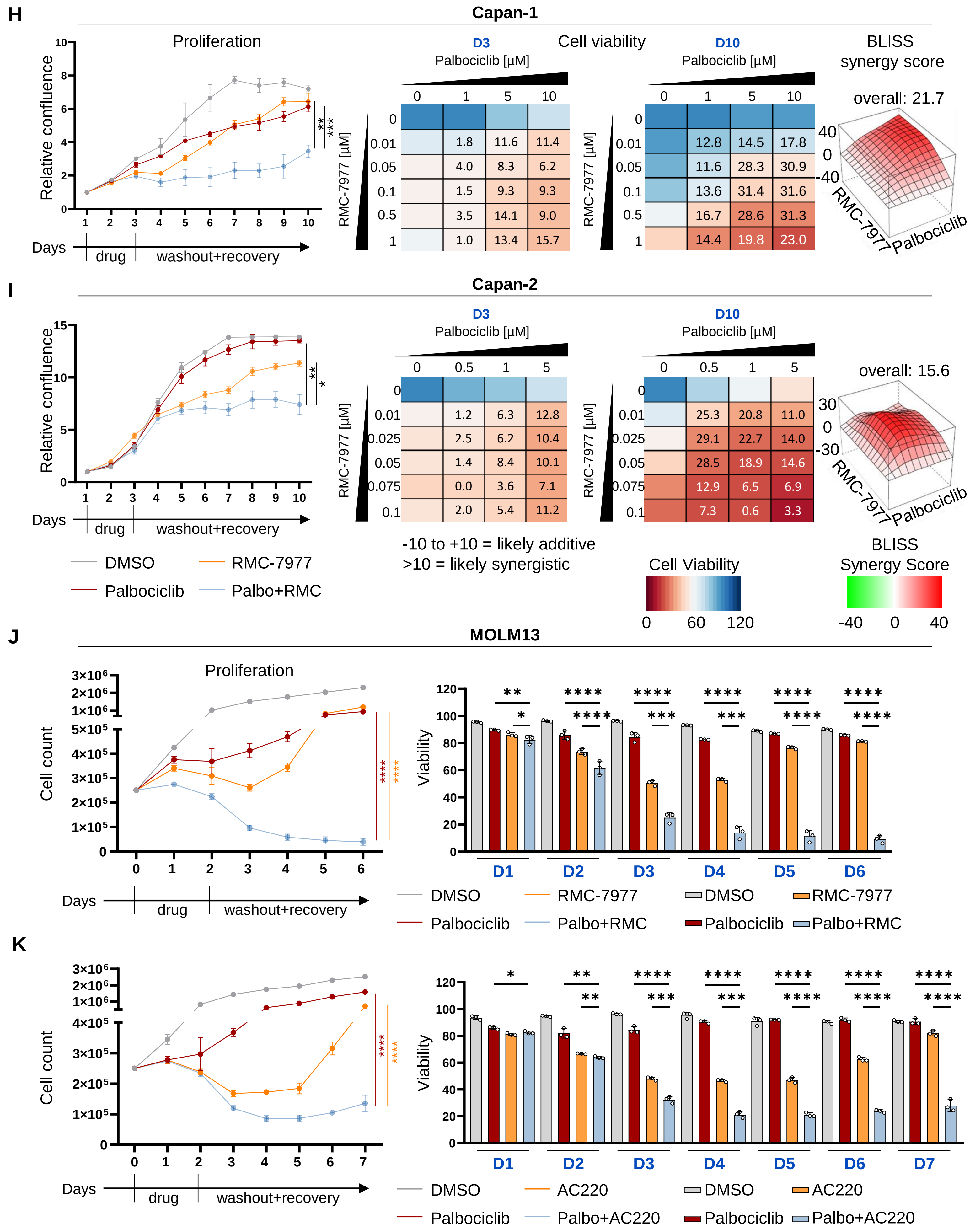

Suppl. Figure 2 continued

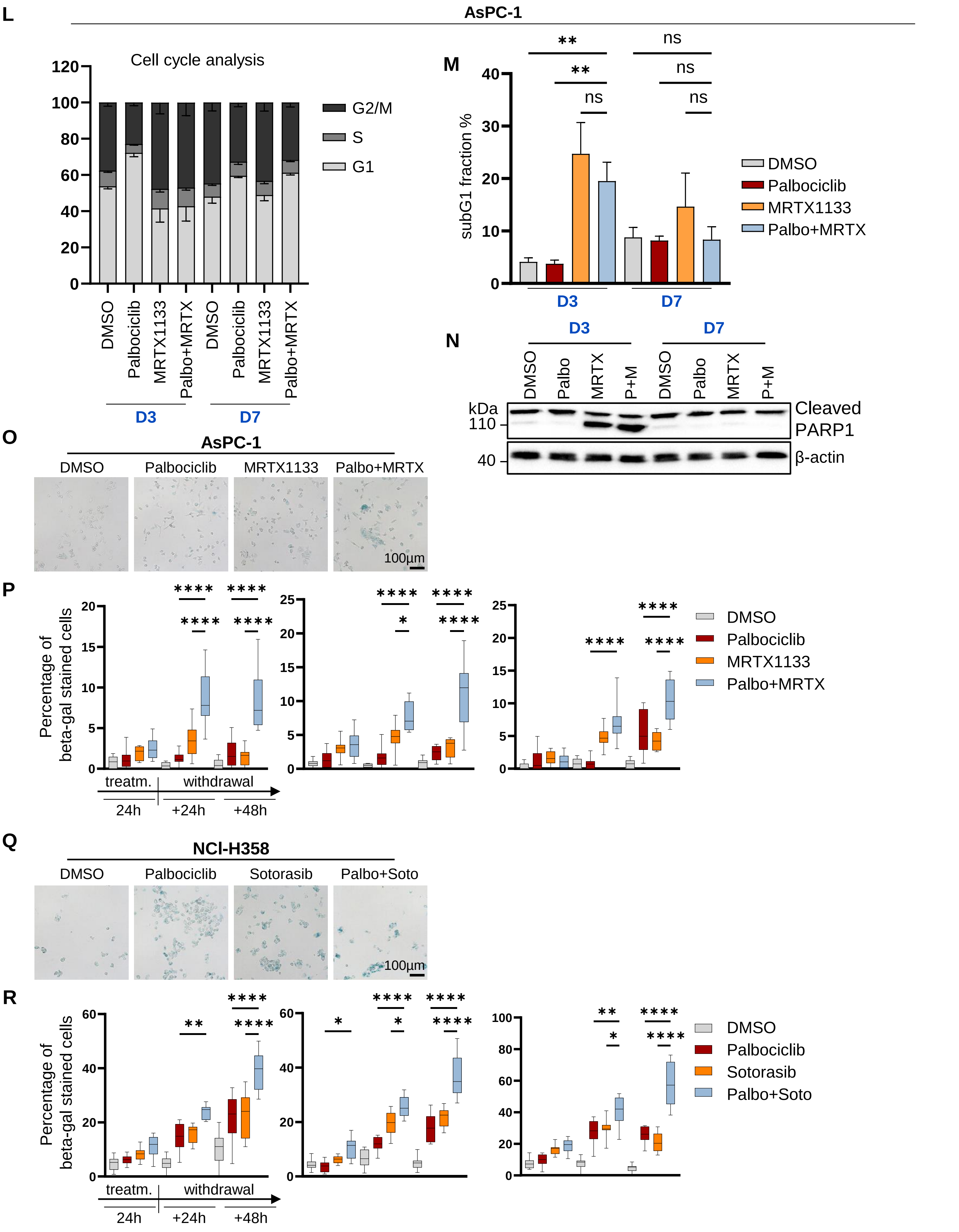

Suppl. Figure 2: Palbociclib and KRAS inhibitors synergize to suppress PDAC and NSCLC cell proliferation.

All assays were carried out as described in the legend to Suppl. Figure 1.

(A) AsPC-1 cells, 5 µM Palbociclib and 0.1 µM MRTX1133, D3 and D10. BLISS scores were determined for D10.
(B) KPC cells, 5 µM Palbociclib and 5 µM MRTX1133, D3 and D10.
(C) NCl-H358 cells, 0.5 µM Palbociclib and 50 nM Sotorasib, D3 and D11.
(D) NCl-H2122 cells, 5 µM Palbociclib and 10 µM Sotorasib, D3 and D8.
(E) GöCDX52 cells, 1 µM Palbociclib, 0.5 µM MRTX1133, D3 and D10.
(F) GöCDX7 cells, 0.5 µM Palbociclib, 0.5 µM MRTX1133, D3 and D10.
(G) GöCDX53 cells, 5 µM Palbociclib, 5 µM MRTX1133, D3 and D10.
(H) Capan-1 cells, 5 µM Palbociclib and 0.1 µM RMC 7977, D3 and D10.
(I) Capan-2 cells, 10 µM Palbociclib and 0.05 µM RMC 7977, D3 and D10.
(J) MOLM 13 cells, 1 µM Palbociclib and 0.1 µM RMC 7977 for 48 h (D2), following four days of recovery in normal medium (D6). Trypan blue exclusion was used to quantify the viability of cells (right). The graphs indicate the means of three biological replicates ± SD.
(K) MOLM 13 cells, 1 µM Palbociclib and 5 nM AC220, D2 and D7 as described in (J).
(L) Quantification of the cell cycle distribution as discribed in Suppl. Figure 1K. AsPC-1 cells were treated with DMSO, 5 µM Palbociclib and 0.5 µM MRTX1133 or the combination for 48 h, followed by four days of recovery in normal medium Data shown as mean ± SD, n=3.
(M) Quantification of the sub-G1 fraction according to (L).
(N) Immunoblot analysis of whole-cell lysates of AsPC-1 corresponding to (L). β-actin served as loading control. n=2.
(O) Representative images of senescence associated beta-galactosidase (SAB) staining in AsPC-1 cells treated with DMSO, 5 µM Palbociclib, 0.1 µM MRTX1133 or the combination for 24 h, followed by 48 h of recovery in normal medium. Brightfield images: sharpen: 75 %, brightness -25 %, contrast +50 %, saturation 120 %. Scale bar 100 µm.
(P) Quantification of beta-galactosidase positive cells was performed using ImageJ. 10 Images were quantified and are shown as mean ± SD; one representative replicate is shown, n=3.
(Q) SAB staining in NCl-H358 cells treated with DMSO, 1 µM Palbociclib, 0.1 µM Sotorasib or the combination for 24 h, followed by 48 h without drugs. Image settings as in (H).
(R) Quantification of NCI-H358 cell senescence as in (P), n=3.

Statistical analyses: A, B, C, D, E, F, G, H, I, J, K unpaired t-test; M, P, R one-way ANOVA followed by Tukey's multiple comparison (A, B, C, D, E, F, G, H, I, J, K: of AUC); ns: not significant, *p ≤ 0.05, **p ≤ 0.01, ***p ≤ 0.001, ****p ≤ 0.0001.

Suppl. Figure 3

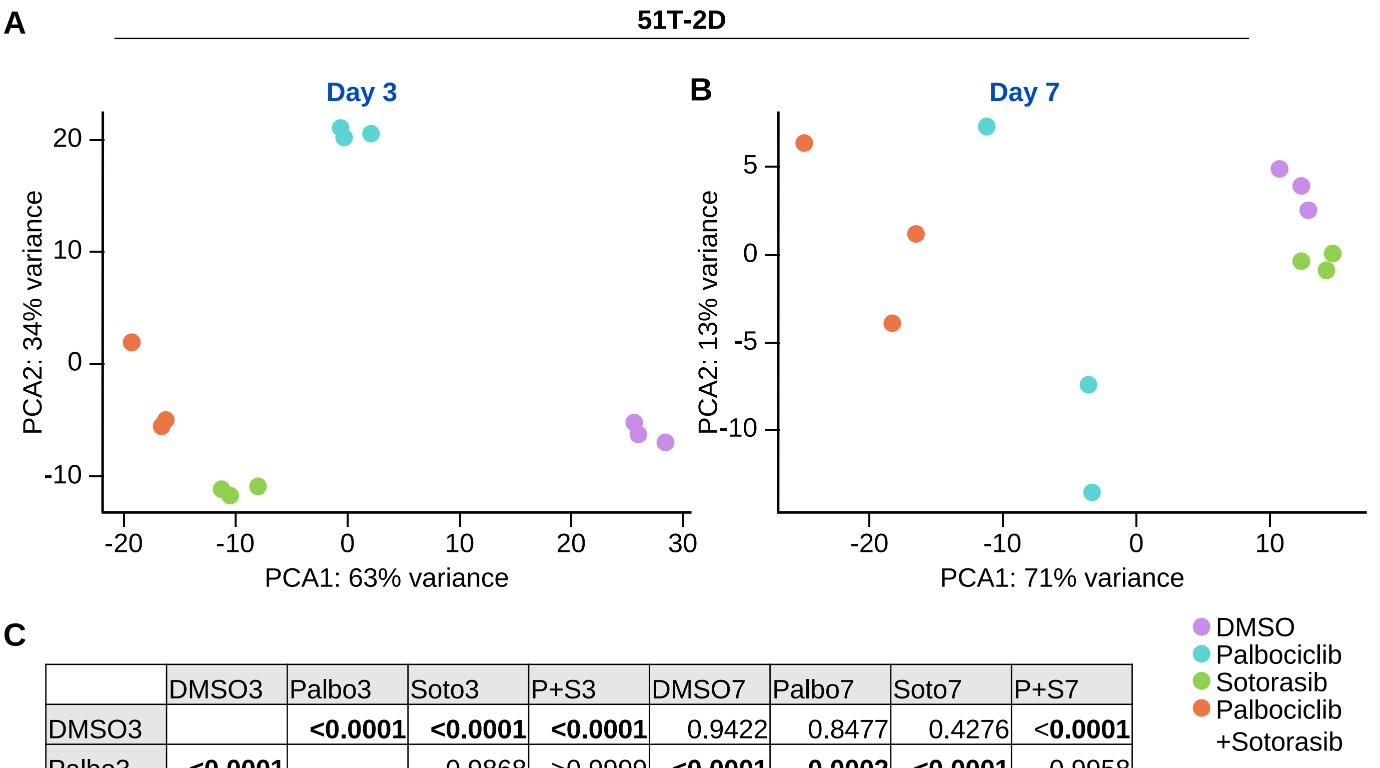

C

	DMSO3	Palbo3	Soto3	P+S3	DMSO7	Palbo7	Soto7	P+S7
DMSO3		**<0.0001**	**<0.0001**	**<0.0001**	0.9422	0.8477	0.4276	<**0.0001**
Palbo3	**<0.0001**		0.9868	>0.9999	**<0.0001**	**0.0002**	**<0.0001**	0.9958
Soto3	**<0.0001**	0.9868		0.9933	**<0.0001**	0.0091	**<0.0001**	>0.9999
P+S3	**<0.0001**	>0.9999	0.9933		**<0.0001**	**0.0004**	**<0.0001**	0.9983
DMSO7	0.9422	<**0.0001**	<**0.0001**	<**0.0001**		0.1625	0.9844	<**0.0001**
Palbo7	0.8477	**0.0002**	**0.0091**	**0.0004**	0.1625		**0.0112**	**0.0052**
Soto7	0.4276	<**0.0001**	<**0.0001**	<**0.0001**	0.9844	**0.0112**		<**0.0001**
P+S7	<**0.0001**	0.9958	>0.9999	0.9983	<**0.0001**	**0.0052**	<0.0001	

Suppl. Figure 3 continued

D

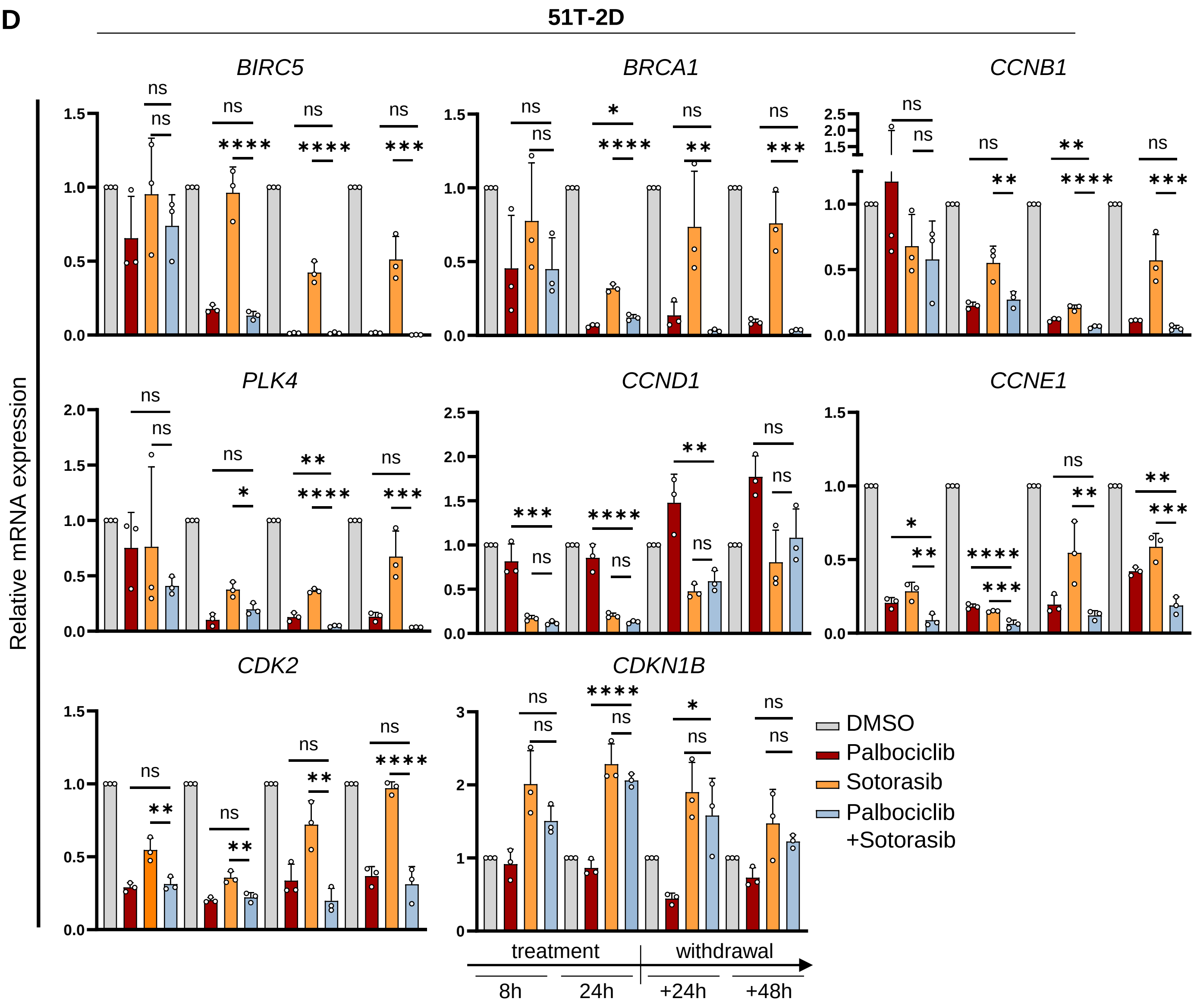

Suppl. Figure 3 continued

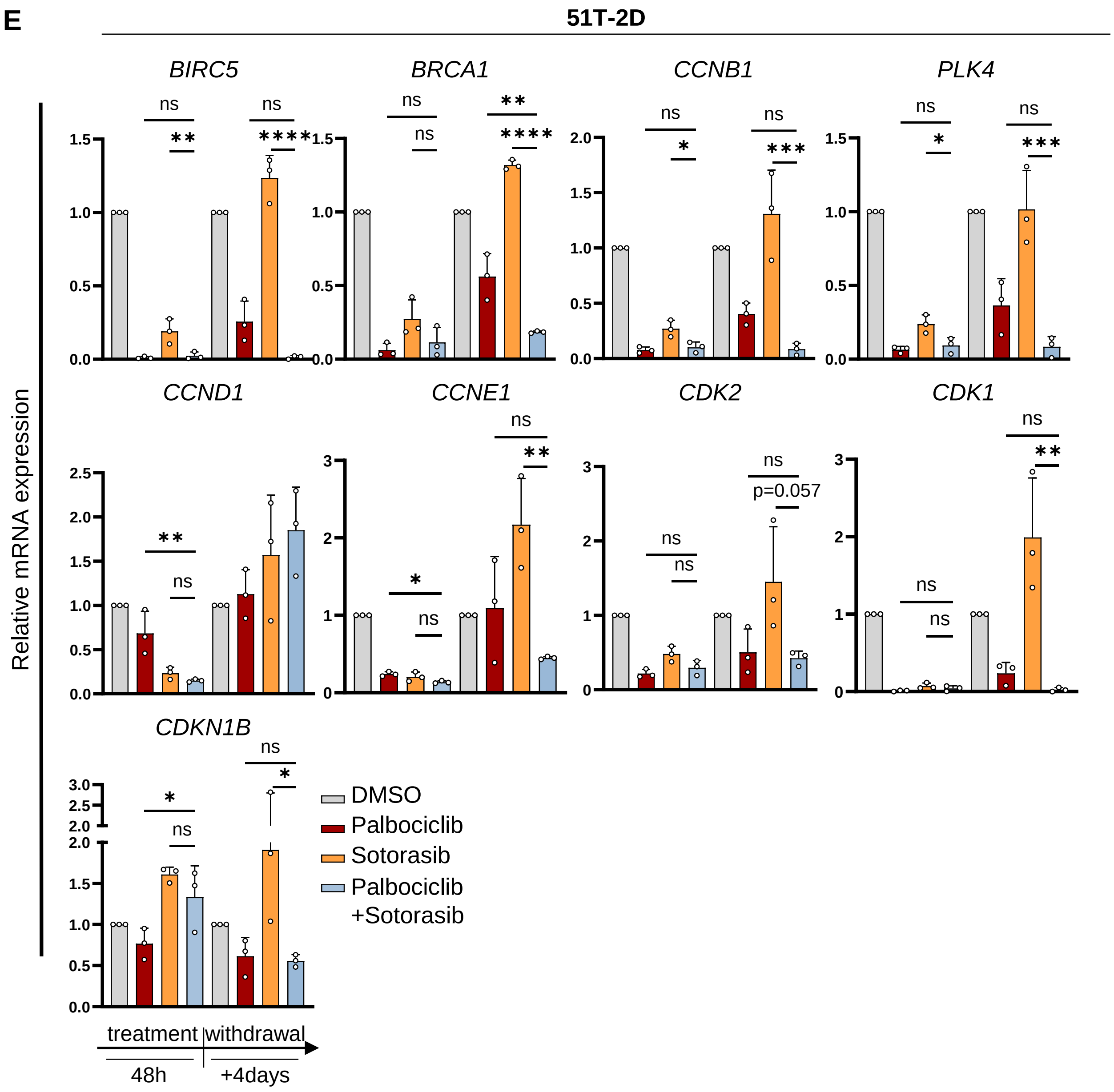

Suppl. Figure 3 continued

F

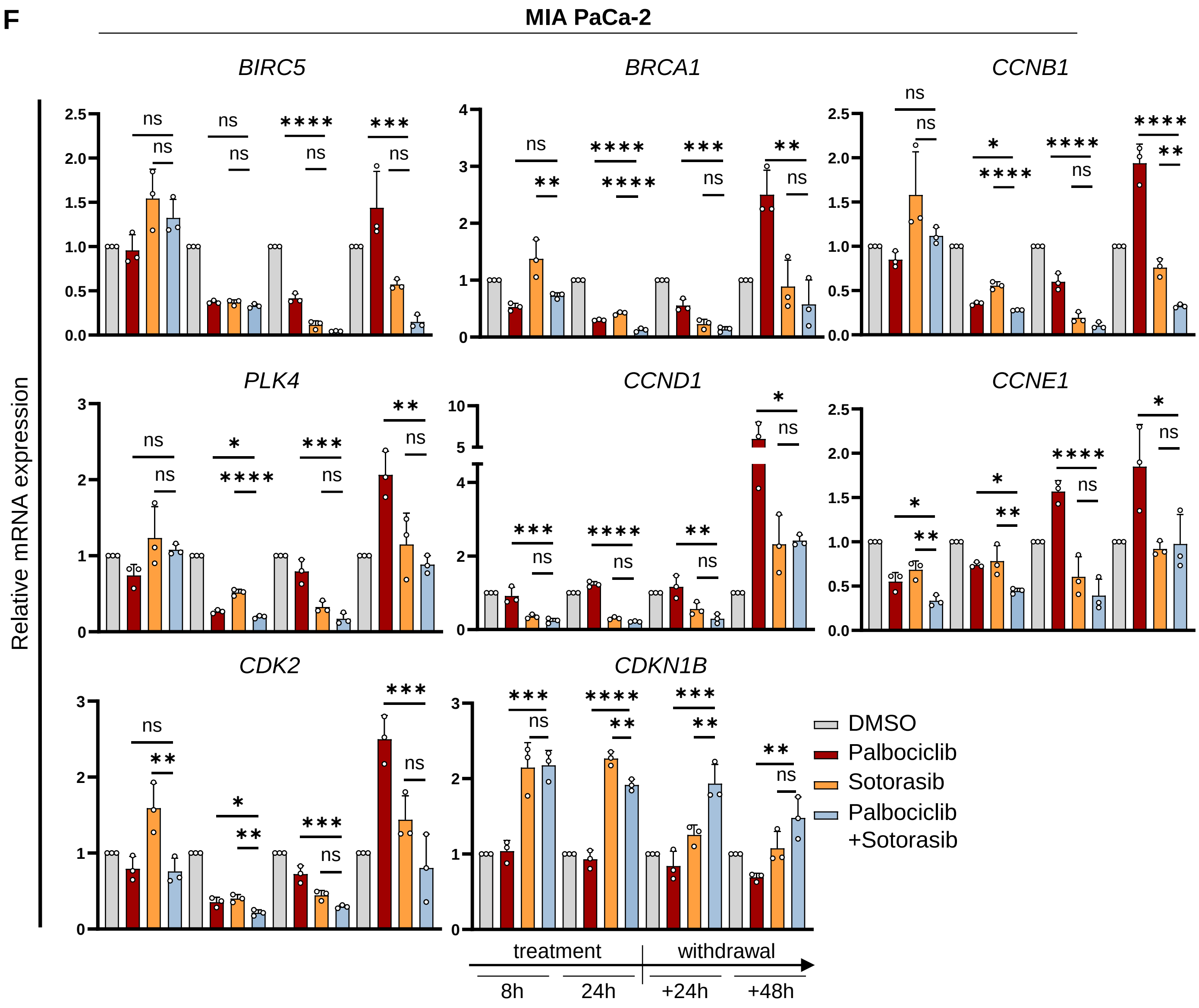

Suppl. Figure 3 continued

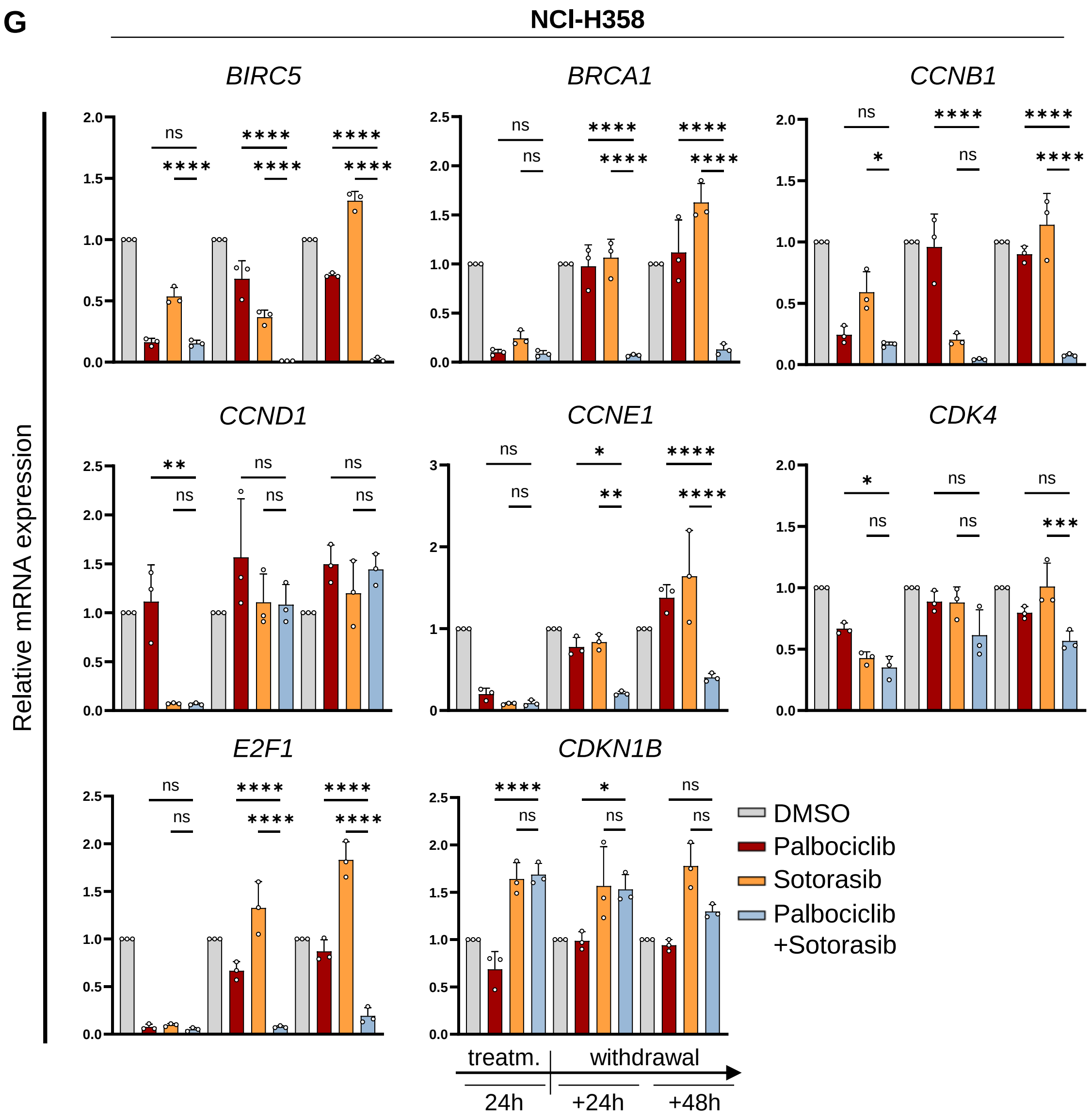

Suppl. Figure 3 continued

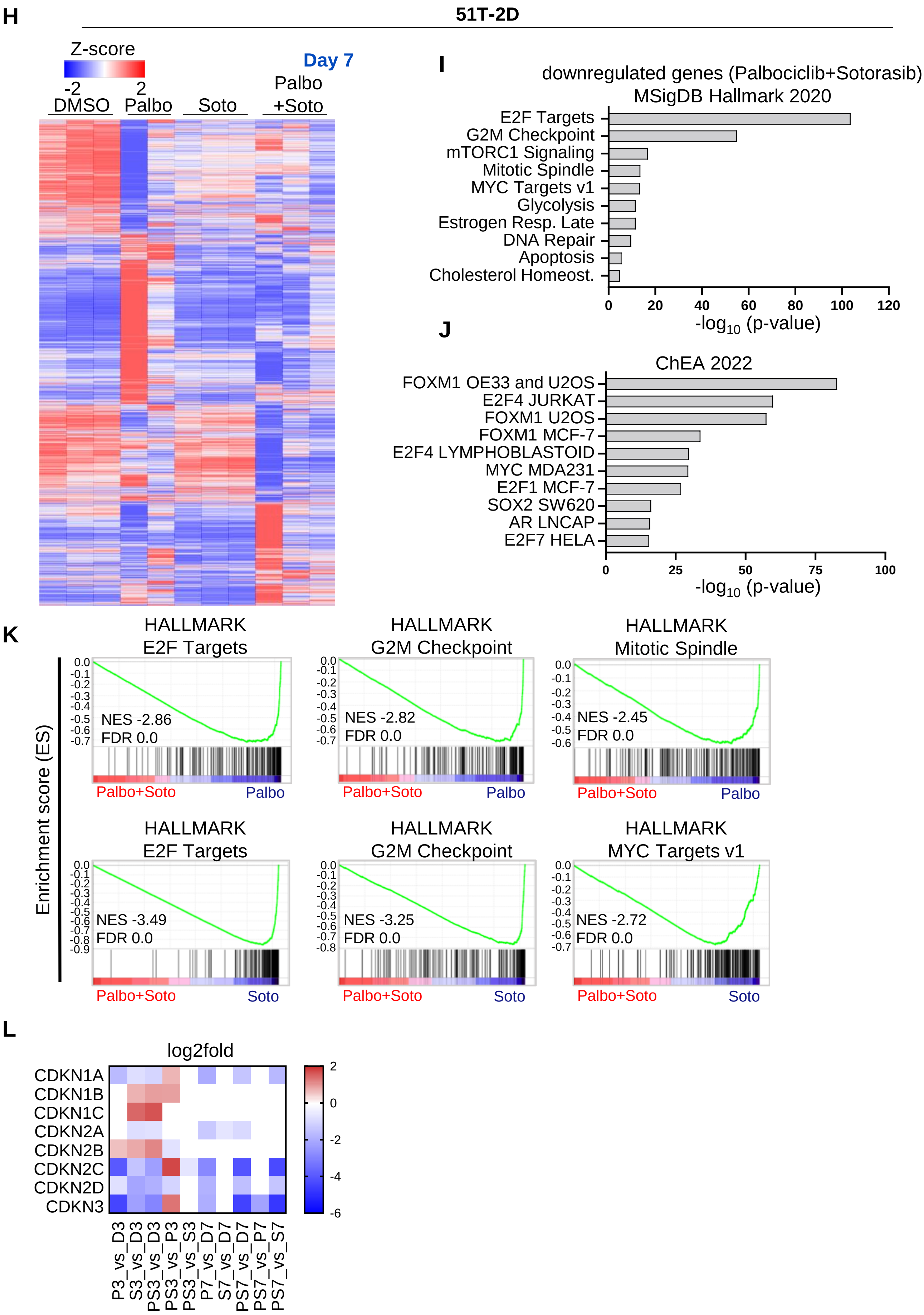

Suppl. Figure 3: Inhibitors of KRAS G12C and CDK4 cooperate to switch the transcriptome towards ceased proliferation.

(A) PCA plot of RNA-seq data derived from 51T-2D cells revealed clustering of treatment on day 3 (48 h of treatment) and (B) day 7 (48 h of treatment + 4 days washout). Upper replicate of Palbociclib day 7 was excluded (Palbociclib day 7: n=2), rest n=3.

(C) Statistical analyses: one-way ANOVA followed by Tukey’s multiple comparison of Figure 3E.

(D) Expression of E2F target genes and cell cycle regulators in 51T-2D cells treated with DMSO, 5 µM Palbociclib, 2.5 µM Sotorasib or the combination of both for 8 and 24 h and washout for 24 or 48 h. Expression levels were normalized to 36B4 mRNA and shown as mean ± SD.

(E) Expression of genes in 51T-2D cells treated and analysed as in (D) for 48 h and washout for four days.

(F) Expression of genes in MIA PaCa-2 cells treated with 10 µM Palbociclib, 5 µM Sotorasib or the combination of both for 8 and 24 h and washout for 24 or 48 h, determined as in (D).

(G) Expression of genes in NCI-H358 cells treated with 1 µM Palbociclib, 0.1 µM Sotorasib or the combination of both for 24 h and washout for 24 or 48 h, determined as in (D).

(H) Heat map depicting differentially expressed genes according to the z-score after performing DeSeq2 analysis four different samples (DMSO, 5 µM Palbociclib, 2.5 µM Sotorasib or combination treatment for 48 h + 4 days washout (Day7), n=3) of 51T-2D cells. Only genes with |log2fold| ≥ 0.6, adjusted p-value (padj.) < 0.05, and baseMean ≥ 15 were included in the analysis.

(I) Downregulated genes upon treatment with Palbociclib + Sotorasib (48 h treatment + 4 days washout) vs DMSO were correlated with the Molecular Signature Database (MSigDB) Hallmark 2020 and ChEA 2022 (J) dataset using the Enrichr platform to identify potentially impaired pathways. (|log2fold| ≥ 0.6, padj. < 0.05, baseMean ≥ 15) Top 10 (ChEA 2022: human), p-value ranked (-log10).

(K) Gene set enrichment analysis (GSEA) of combination treatment vs Palbociclib or Sotorasib after 48 h of treatment + 4 days washout (D7) of Hallmarks (h.all.v2023.2).

(L) Expression of cyclin dependent kinase inhibitors after 48 h treatment (3) or 48 h treatment + 4 days of drug withdrawal (7): |log2fold| ≥ 0.6.

Statistical analyses: D, E, F, G one-way ANOVA followed by Tukey’s multiple comparison; ns: not significant, *p ≤ 0.05, **p ≤ 0.01, ***p ≤ 0.001, ****p ≤ 0.0001.

Suppl. Figure 4

A

AsPC-1

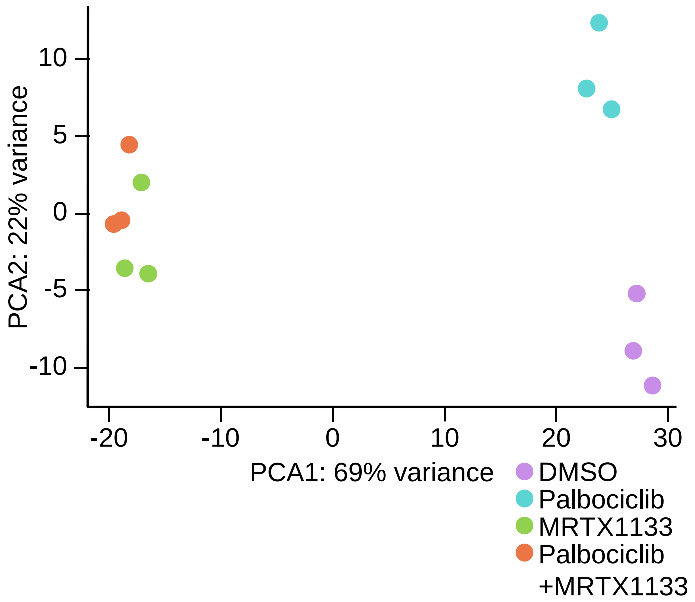

B

	DMSO	Palbo	MRTX	P+M
DMSO		**0.0005**	**<0.0001**	**<0.0001**
Palbo	**0.0005**		0.7890	0.5862
MRTX	**<0.0001**	0.7890		0.9871
P+M	**<0.0001**	0.5862	0.9871	

C

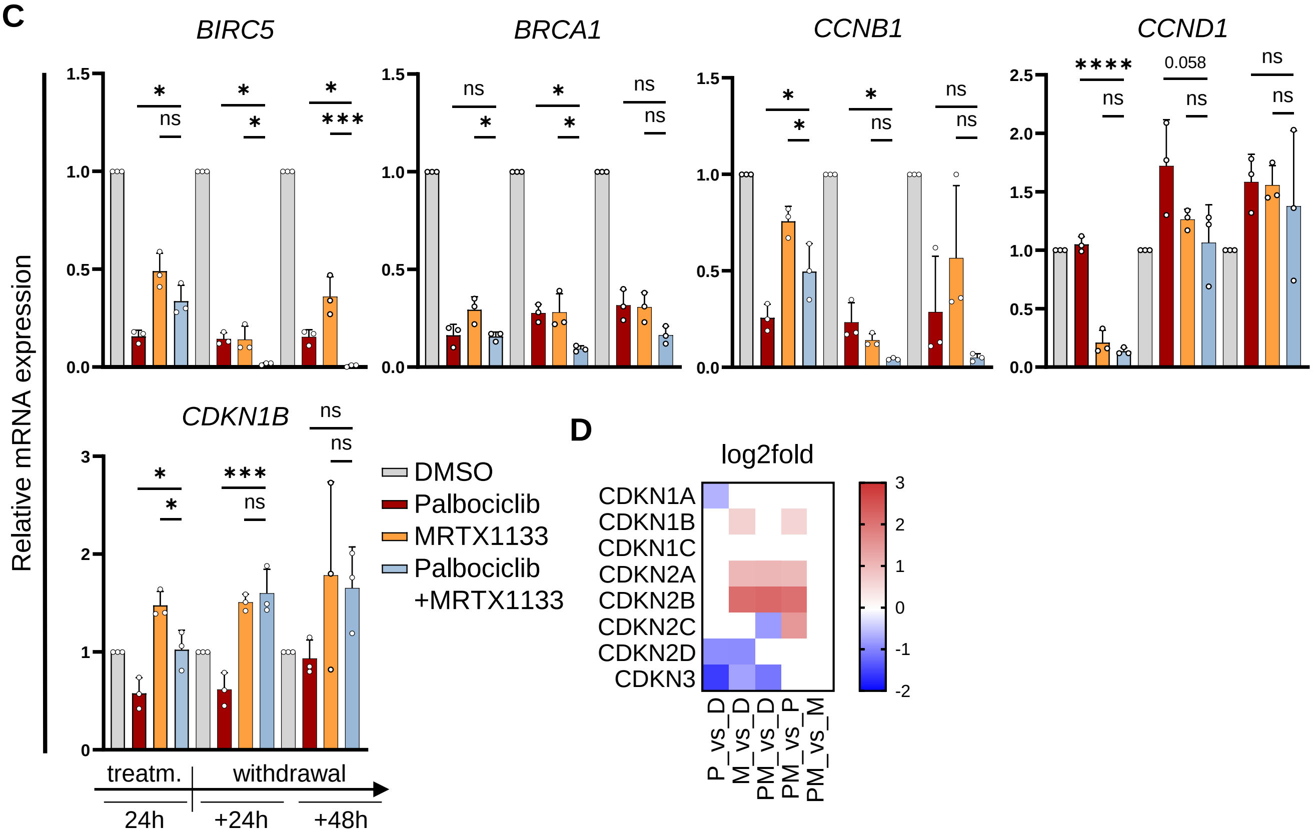

Suppl. Figure 4 continued

E

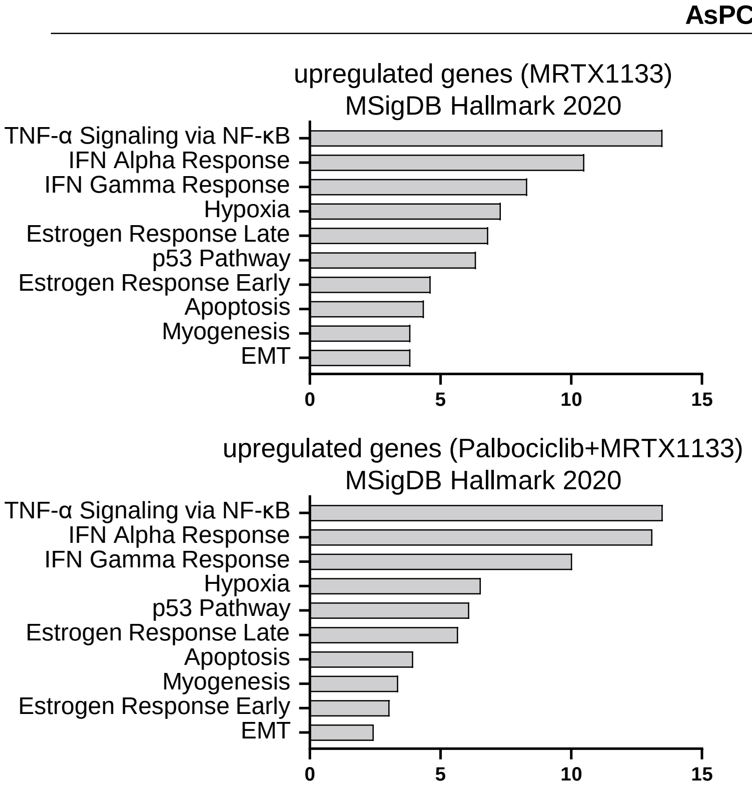

F

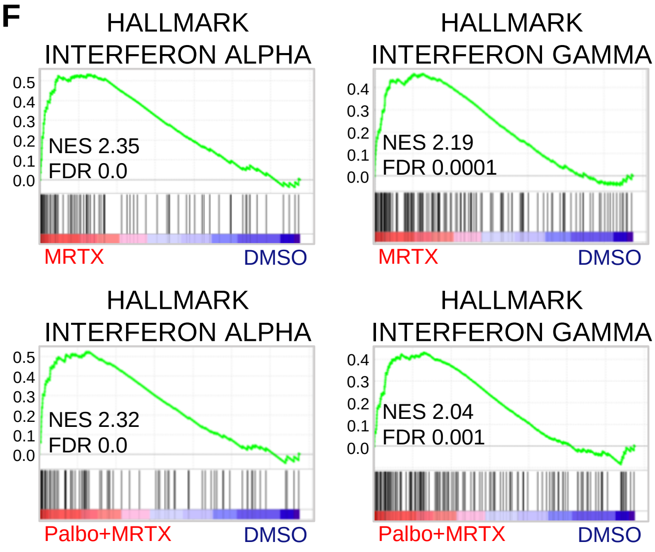

Suppl. Figure 4: Transcriptome alterations in PDAC cells treated with Palbociclib and MRTX1133.

(A) PCA plot of RNA-seq data derived from AsPC-1 cells indicating clustering of the treatment groups on day 2 (24 h of treatment), n=3.
(B) Statistical analyses: one-way ANOVA followed by Tukey's multiple comparison of Figure 4E.
(C) Expression of E2F target genes and cell cycle regulators in AsPC-1 cells treated with 5 µM Palbociclib, 0.5 µM MRTX1133 or the combination of both for 24 h and washout for 24 or 48 h. Expression levels were normalized to 36B4 mRNA and shown as mean ± SD.
(D) Expression of cyclin dependent kinase inhibitors after 24 h treatment: |log2fold| ≥ 0.6.
(E) Upregulated genes in MRTX1133 or Palbo+MRTX (24 h treatment) vs DMSO were correlated with the Molecular Signature Database (MSigDB) Hallmark 2020 dataset, using the Enrichr platform to identify upregulated pathways. Top 10, p-value ranked (log10).
(F) Gene set enrichment analysis (GSEA) of MRTX1133 or combination treatment vs DMSO (control) after 24 h of treatment; revealing Interferon-responsive genes induced by KRAS inhibition hallmarks (h.all.v2023.2).

Statistical analyses: B, C one-way ANOVA followed by Tukey's multiple comparison; ns: not significant, *p ≤ 0.05, **p ≤ 0.01, ***p ≤ 0.001, ****p ≤ 0.0001.

Suppl. Figure 5

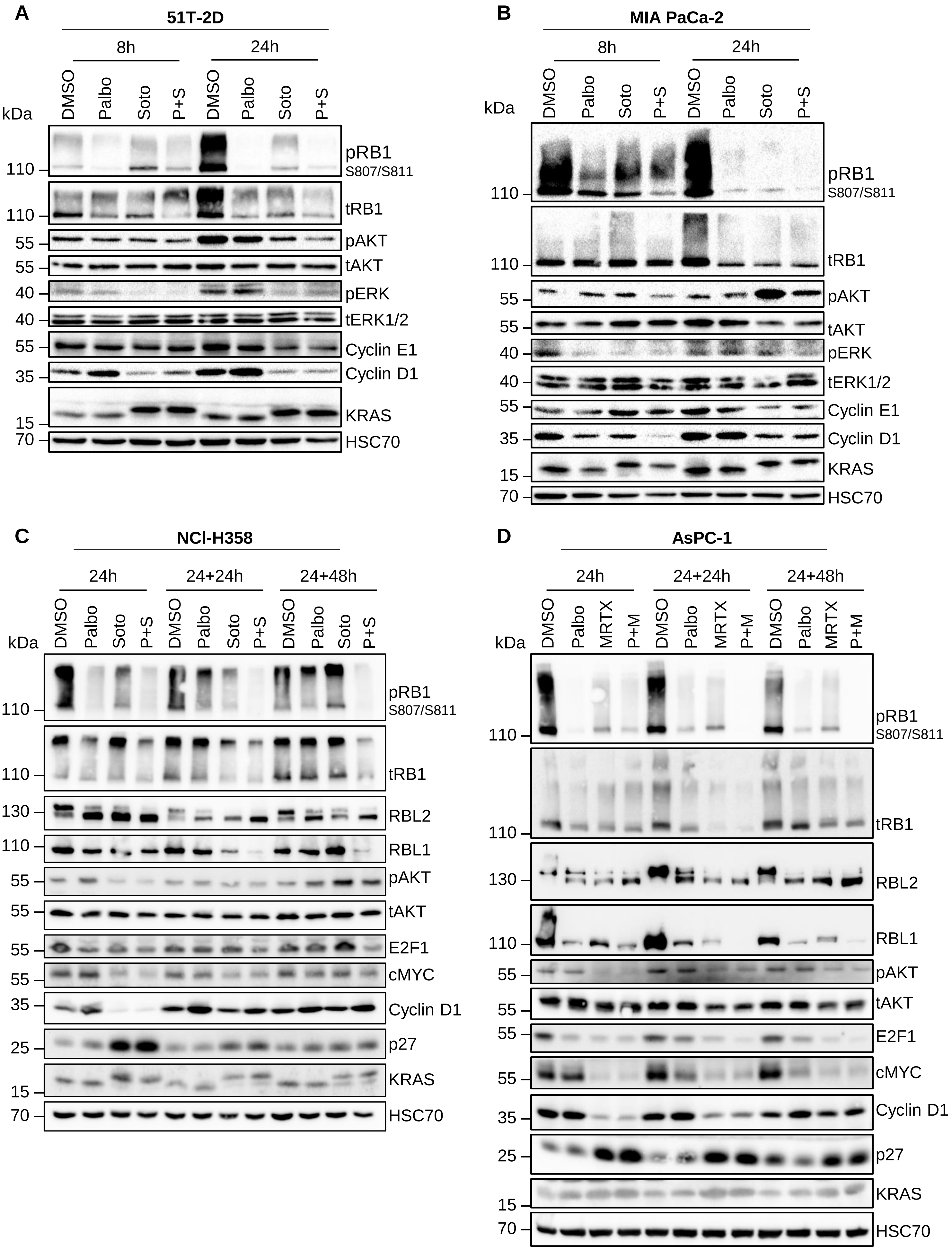

Suppl. Figure 5: Upon inhibition of KRAS and CDK4, E2F1 largely disappears while CDKN1B/p27 levels increase and RB family proteins adopt a hypophosphorylated state.

(A) Immunoblot analysis of whole-cell lysates of 51T-2D cells treated with DMSO, 5 µM Palbociclib, 2.5 µM Sotorasib or combination treatment for 8 or 24 h. HSC70 served as sample control. One representative immunoblot shown; n=2.
(B) MIA PaCa-2 cells treated with 10 µM Palbociclib and/or 5 µM Sotorasib for 8 or 24 h. n=2.
(C) NCl-H358 cells treated with 1 µM Palbociclib and/or 0.1 µM Sotorasib for 24 h and washout for 24 or 48 h. n=2.
(D) AsPC-1 cells treated with 5 µM Palbociclib and/or 0.5 µM MRTX1133 for 24 h and washout for 24 or 48 h. n=2.

Suppl. Figure 6

A

MIA PaCa-2

	RB1	RBL1	RBL2	E2F4	FOS	CDKN1B (1 µM)	CDKN1B (2.5 µM)	CDKN1B (5 µM)
DMSO (scrb) vs. (target)	0.6663	0.6663	0.6663	0.6663	**0.0077**	**0.0073**	**0.0073**	**0.0073**
Palbociclib (scrb) vs. (target)	**0.0179**	0.1933	**0.0069**	0.3880	**0.0012**	**0.0237**	**0.0201**	0.1114
Sotorasib (scrb) vs. (target)	**0.0012**	**0.0004**	**0.0078**	**0.0006**	**0.0002**	**0.0035**	**0.0035**	**0.0035**
Palbo+Soto (scrb) vs. (target)	**0.0002**	0.3435	0.0672	0.0700	0.0707	**0.0066**	**0.0020**	0.0728

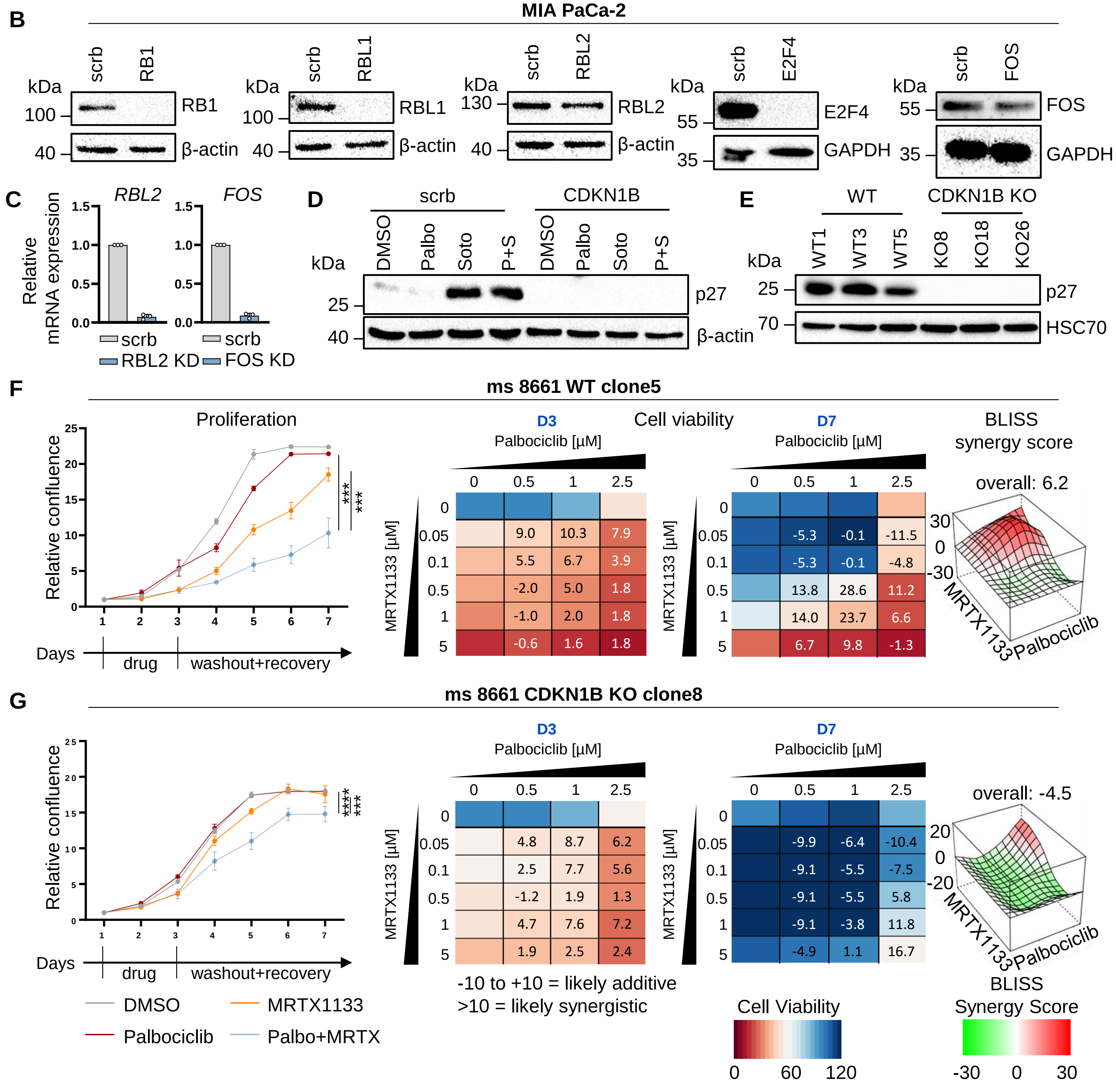

Suppl. Figure 6 continued

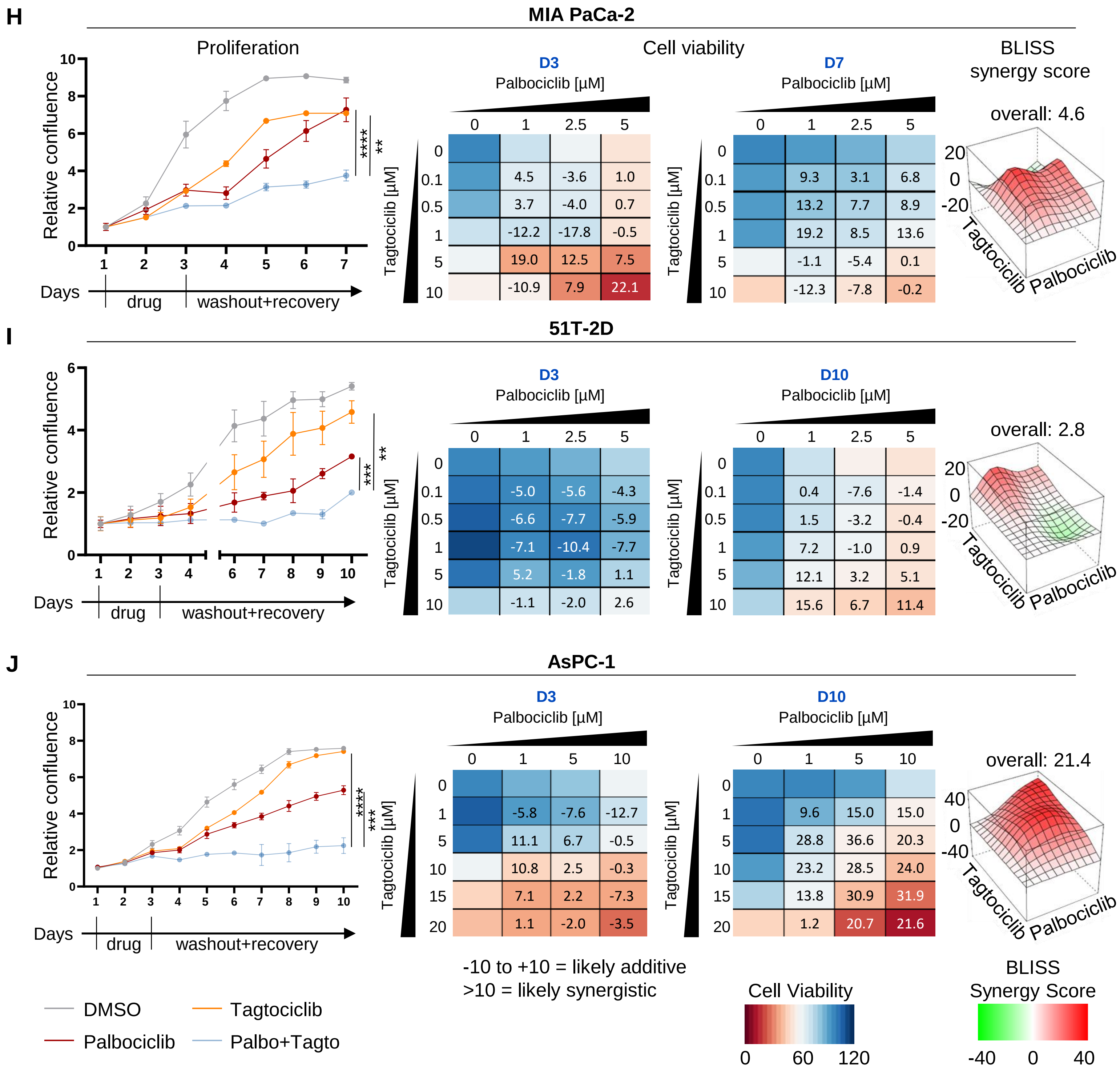

Suppl. Figure 6: Growth suppression by inhibitors of KRAS and CDK4 depends on the RB family proteins and p27.

(A) Statistical analyses corresponding to Figure 6 A, B, C, D, E, F, G, H unpaired t-test of. Bold: $p < 0.05$; scrb = control siRNA; target = target siRNA.
(B) Immunoblot analysis of MIA PaCa-2 cells corresponding to Figure 6 A, B, C, D, E confirming knockdowns. HSC70/GAPDH/β-actin served as loading control.
(C) Expression of RBL2/FOS in MIA PaCa-2 cells after knockdown of RBL2 or FOS corresponding to Figure 6 C, E. Scrb siRNA transfected cells served as controls. Data was normalized to 36B4 mRNA and is shown as mean ± SD, n=3.
(D) Immunoblot analysis of MIA PaCa-2 cells corresponding to (Figure 6 F, G, H) confirming knockdowns. β-actin served as loading control.
(E) Immunoblot analysis of 8661 cells CDKN1B WT clones 1, 3, 5 and CDKN1B knock-out KO clones 8, 18, 26 confirming the knock-out of CDKN1B.
(F) 8661 WT (F) and CDKN1B knock-out (G) were treated and observed as in Suppl. Figure 1, with the following conditions: 1 µM Palbociclib, 0.5 µM MRTX1133 or the combination. Evaluation at D3 and D7.
(H) Proliferation of MIA PaCa-2 cells as in Suppl. Figure 1, 5 µM Palbociclib, 1 µM Tagtociclib, evaluation at D3 and D7.
(I) Proliferation of 51T-2D cells as in Suppl. Figure 1, 5 µM Tagtociclib, 1 µM Palbociclib, D3 and D10.
(J) Proliferation of AsPC-1 cells as in Suppl. Figure 1 5 µM Palbociclib, 5 µM Tagtociclib, D3 and D10.

Statistical analyses: A, C, F, G, H, I, J unpaired t-test (A, F, G, H, I, J: of AUC); ns: not significant, $^*p \leq 0.05$, $^{**}p \leq 0.01$, $^{***}p \leq 0.001$, $^{****}p \leq 0.0001$.

Suppl. Figure 7

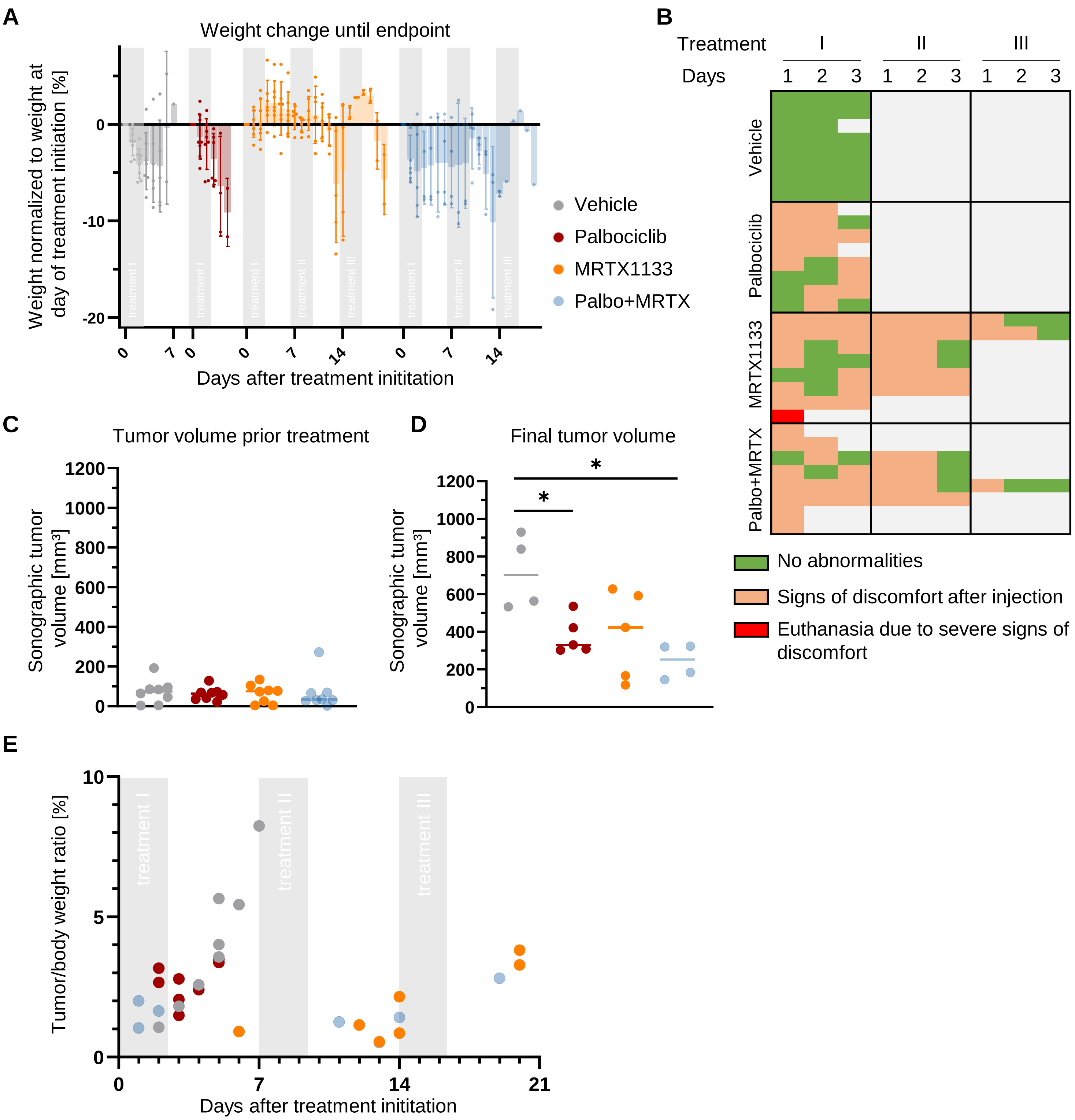

Suppl. Figure 7 continued

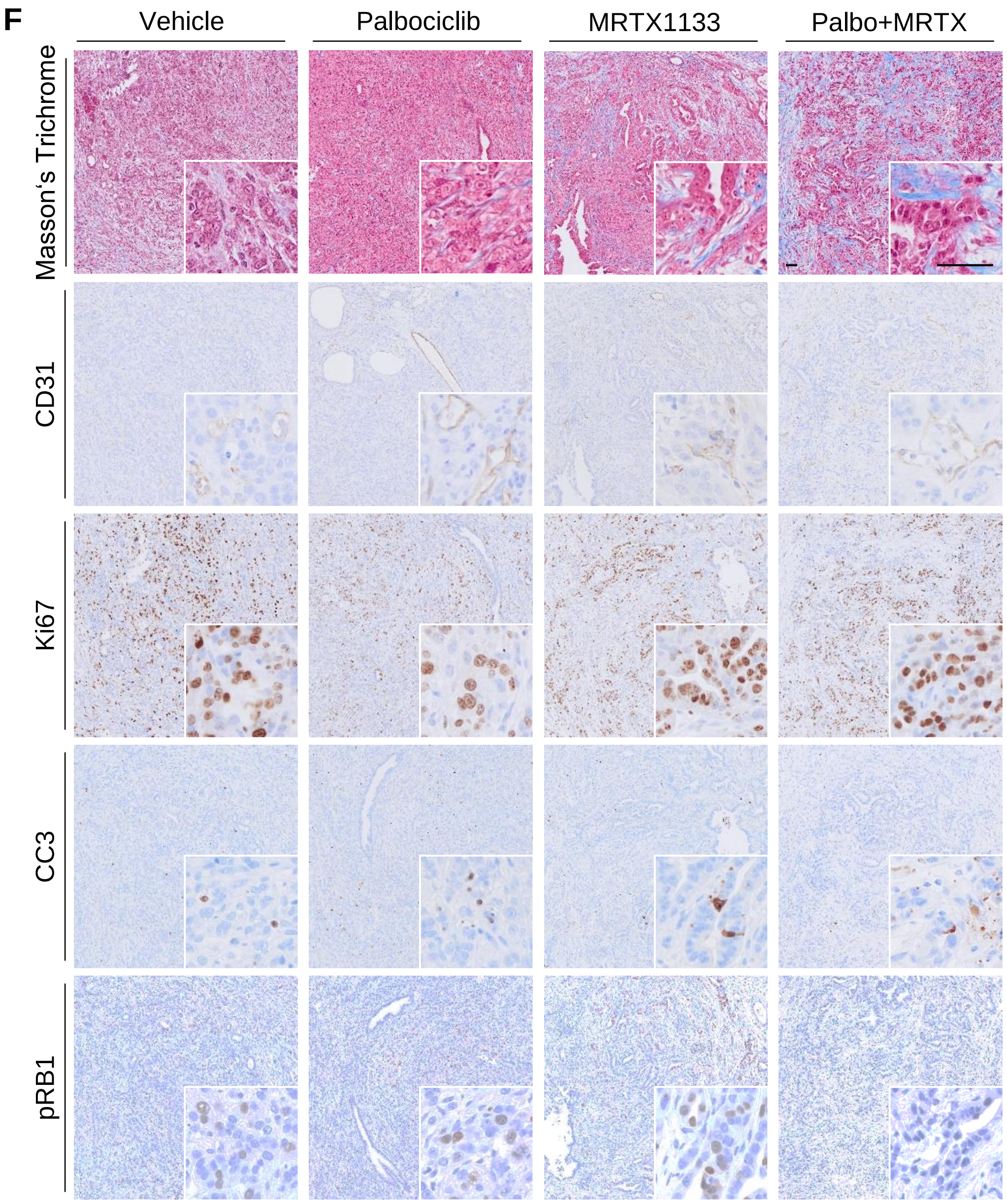

Suppl. Figure 7 continued

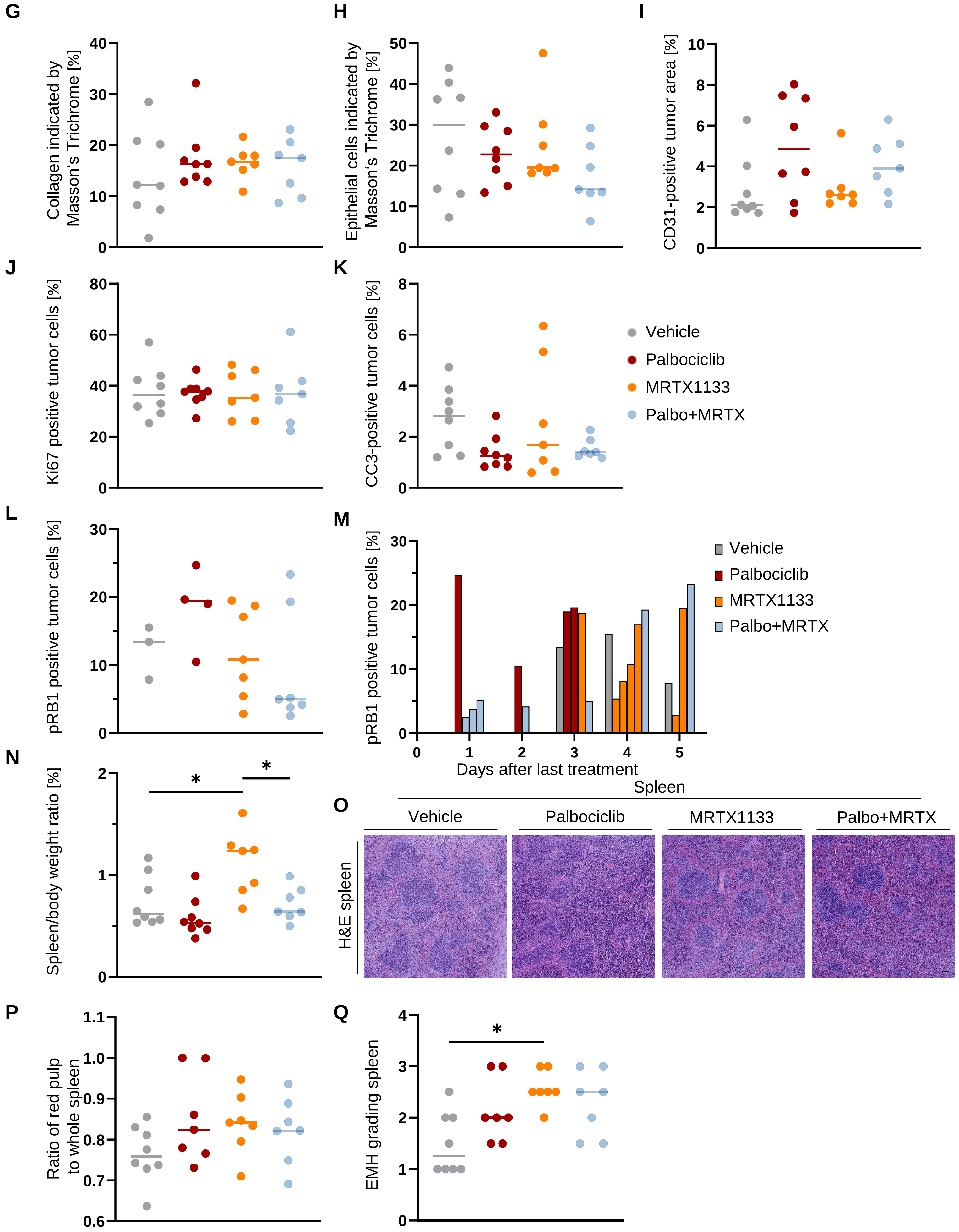

Suppl. Figure 7 continued

R

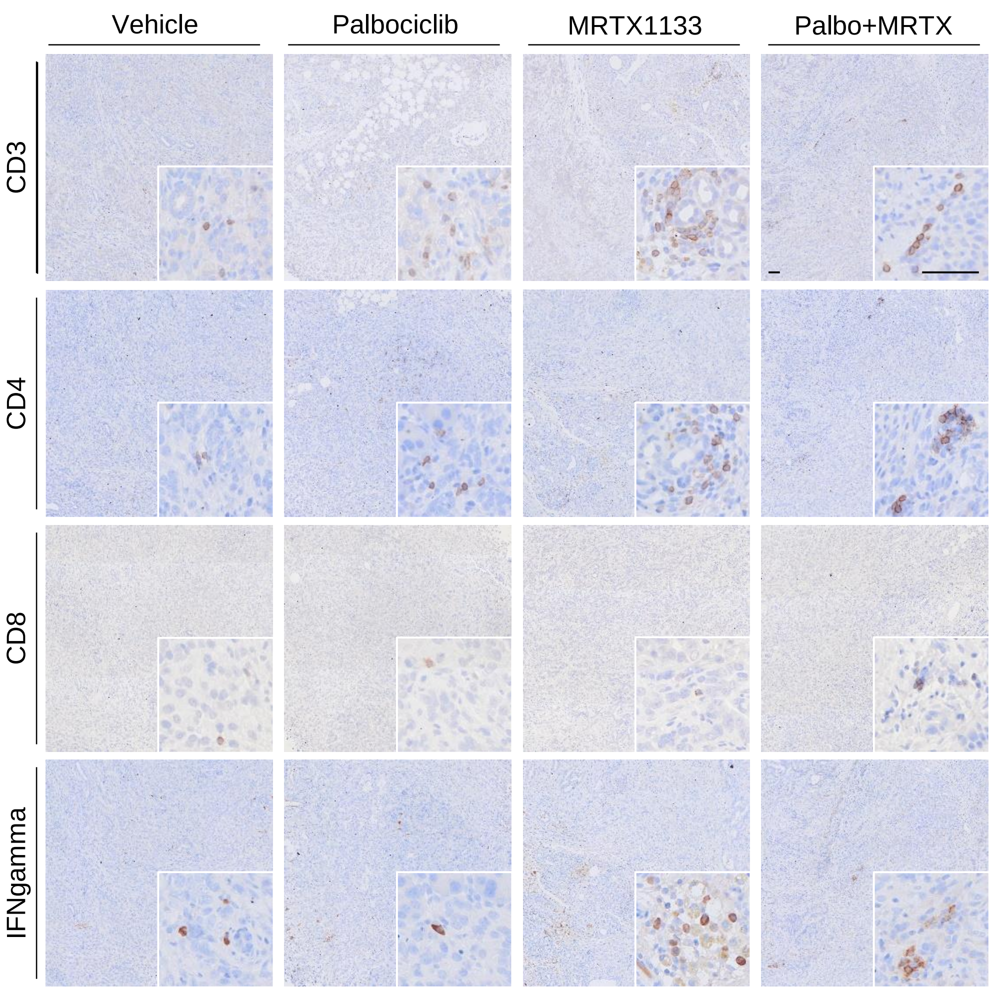

S

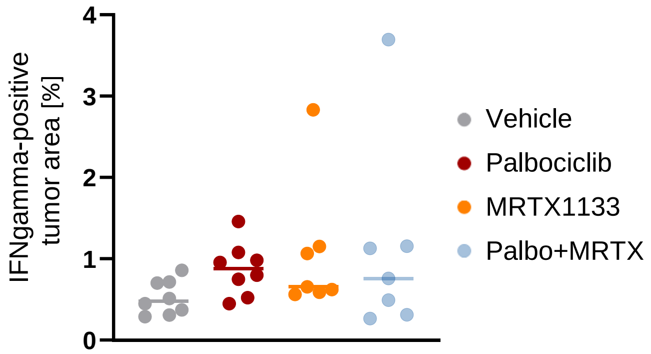

Suppl. Figure 7 continued

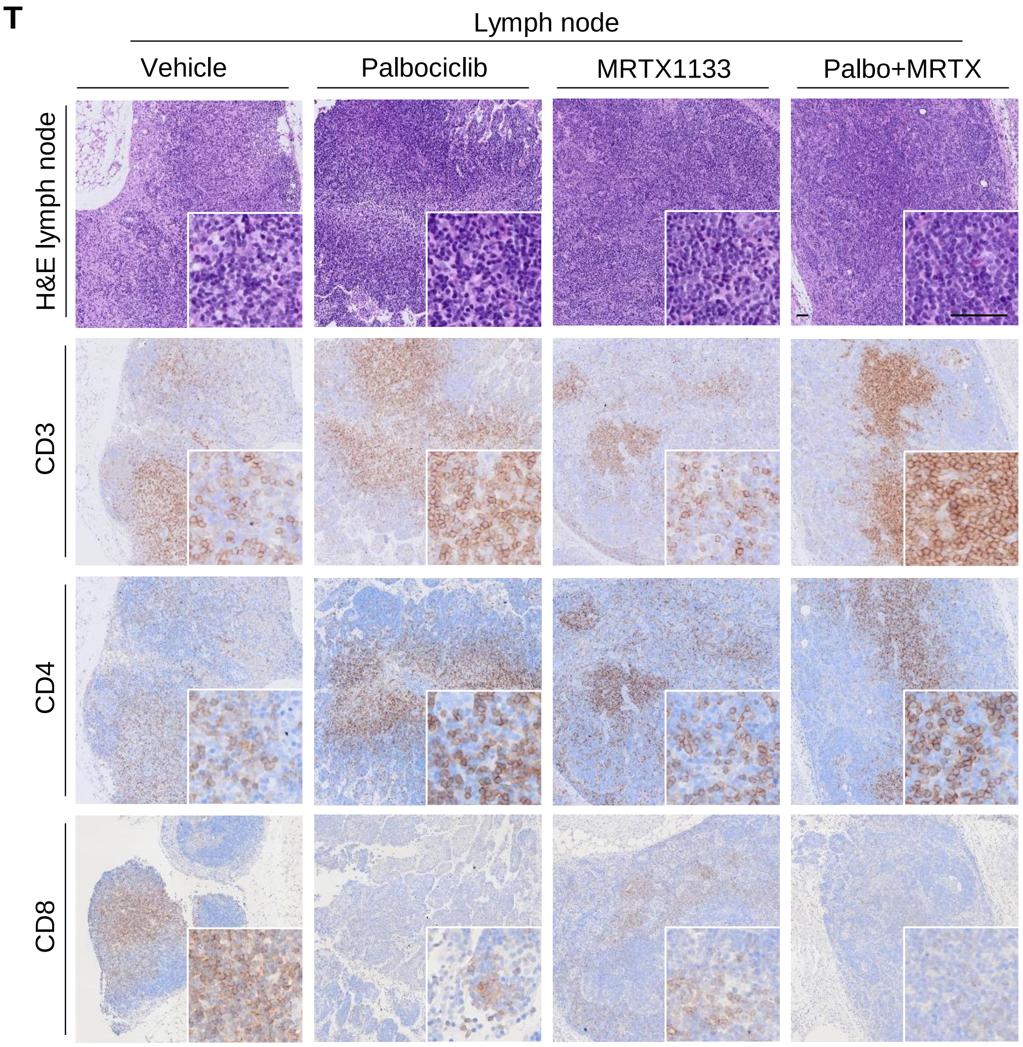

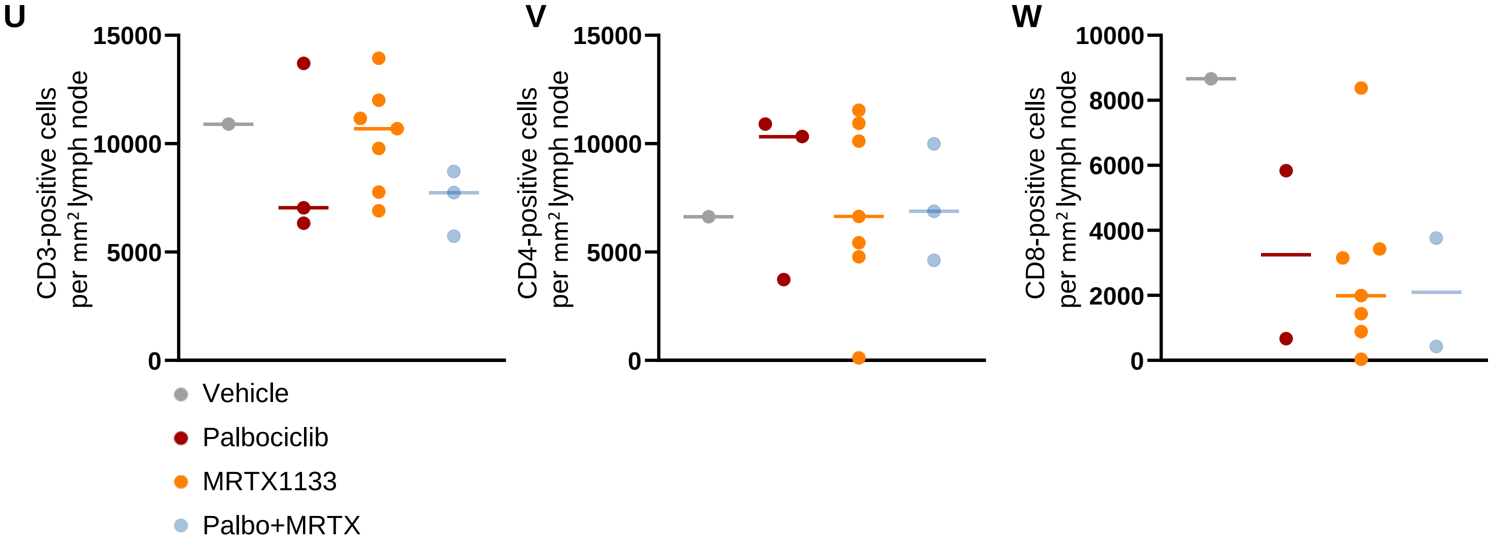

Suppl. Figure 7 continued

X

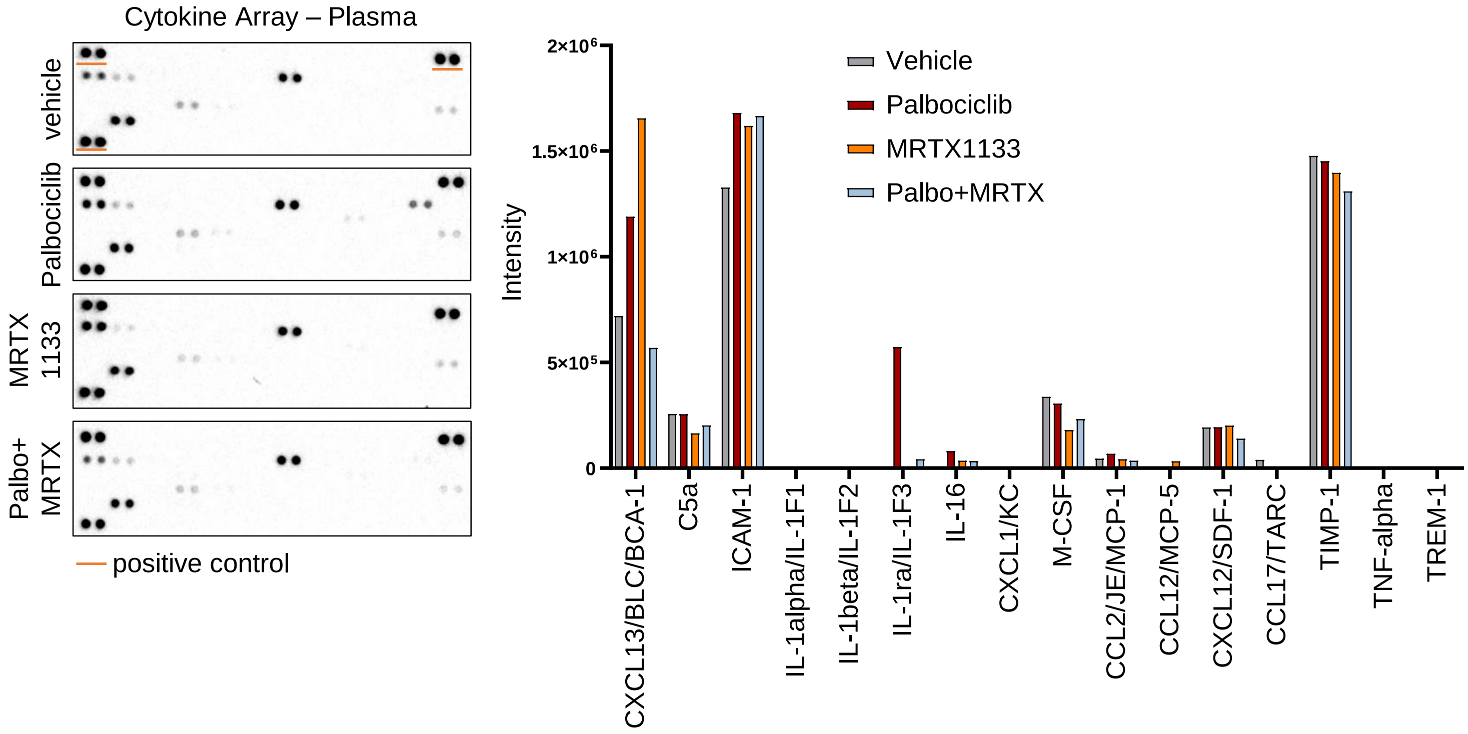

Y

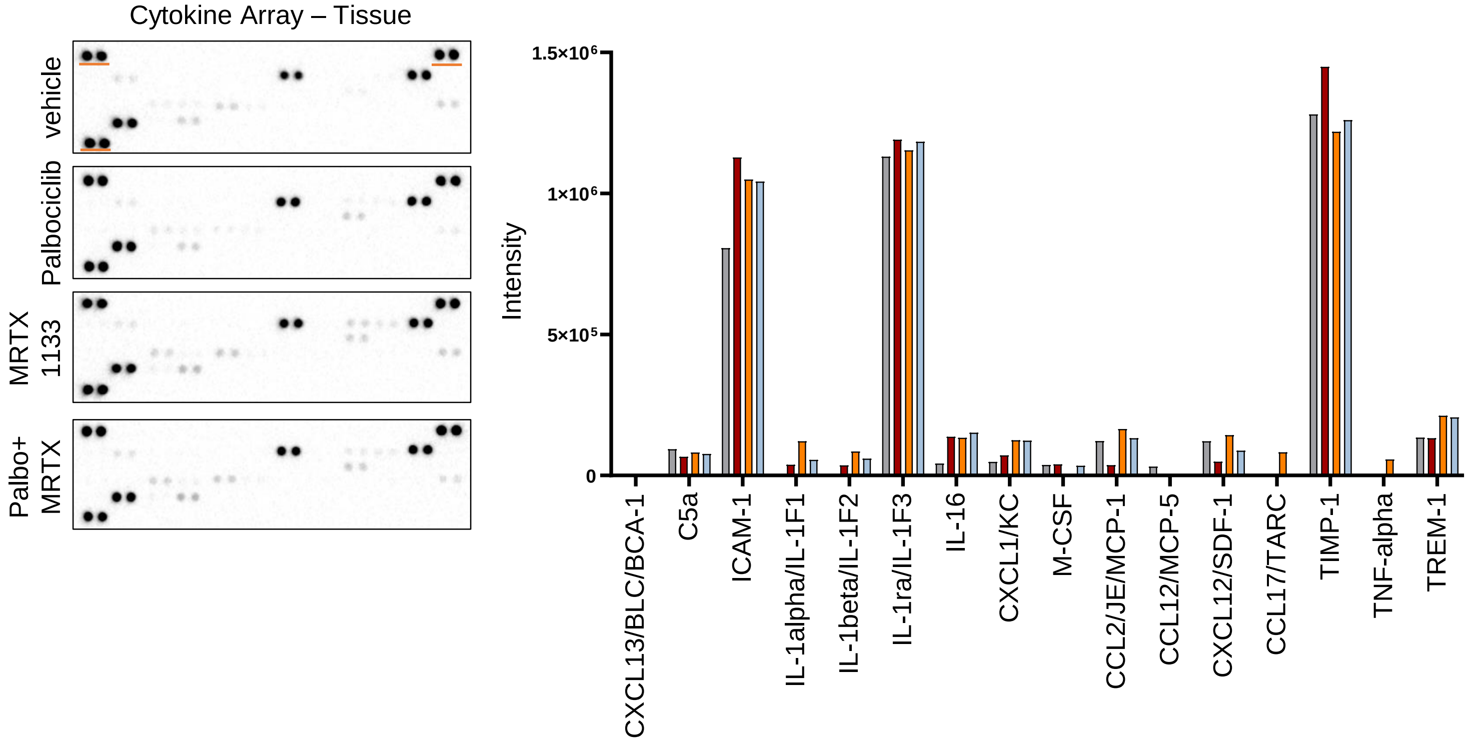

Suppl. Figure 7: CDK4/6 inhibition does not further enhance the efficacy of KRASG12D inhibition in an immunocompetent orthotopic PDAC model.

(A) Relative weight loss over time under treatment normalized to weight before treatment initiation across the four treatment groups. Treatment days are indicated.
(B) Overview of observed side effects per mouse within 1 to 5 h after i.p. injection of the respective drug combinations. Signs of discomfort served as indication for analgetic treatment.
(C) Tumor volumes were detected via ultrasound prior to treatment initiation.
(D) Tumor volumes at final endpoint detected via sonography (4-5 animals each group).
(E) Comparison of tumor to body weight ratio between the different treatment groups over time after treatment initiation.
(F) Representative images of immunostainings of the four treatment groups of quantifications in G-L. Scale bar 50 µm.
(G) Quantification of the following immunostainings: Masson’s Trichrome to analyse the collagen or epithelial fraction (G, H), vascularization as CD31 positive tumor area (I), proliferation as Ki67 positive tumor cells (J), apoptosis as cleaved caspase 3 (CC3) positive tumor cells (K), phosphorylated RB1 (pRB1) positive tumor cells (L), quantification of pRB1 positive tumor cells with respect to the latency upon last drug administration (M).
(N) Comparison of spleen to body weight ratio between the different treatment groups.
(O) Representative images of H&E staining of spleens. Scale bar 50 µm.
(P) Quantified ratio of red pulp to whole spleen of H&E staining of spleens.
(Q) Grading of extra medullary hematopoesis (EMH) in spleens. The degree of EMH was scored by pathologist M.B.
(R) Representative images of immunostainings of the tumors of the four treatment groups corresponding to Figure 7E, F, G, and (S).
(S) Quantification of interferon gamma positive tumor area.
(T) Representative images of immunostainings of lymph nodes of quantifications in (U-W) including H&E staining.
(U) Quantification of CD3 (U), CD4 (V) and CD8 (W) positive cells per lymph node. A different number of lymph nodes was detected for each treatment group.
(X) Cytokine array performed on plasma (X) or tissue (Y) samples of the four treatment groups. Quantification (right) of positive signals from the membranes (material from two mice pooled from each treatment group).

Statistical analyses: D, G, H, I, J, K, L, N, P, S, U, V, W one-way ANOVA followed by Tukey’s multiple comparison; Q Kruskal-Wallis Test and post hoc Dunn’s multiple comparison; ns: not significant, $*p \leq 0.05$, $**p \leq 0.01$, $***p \leq 0.001$, $****p \leq 0.0001$.

Suppl. Figure 8

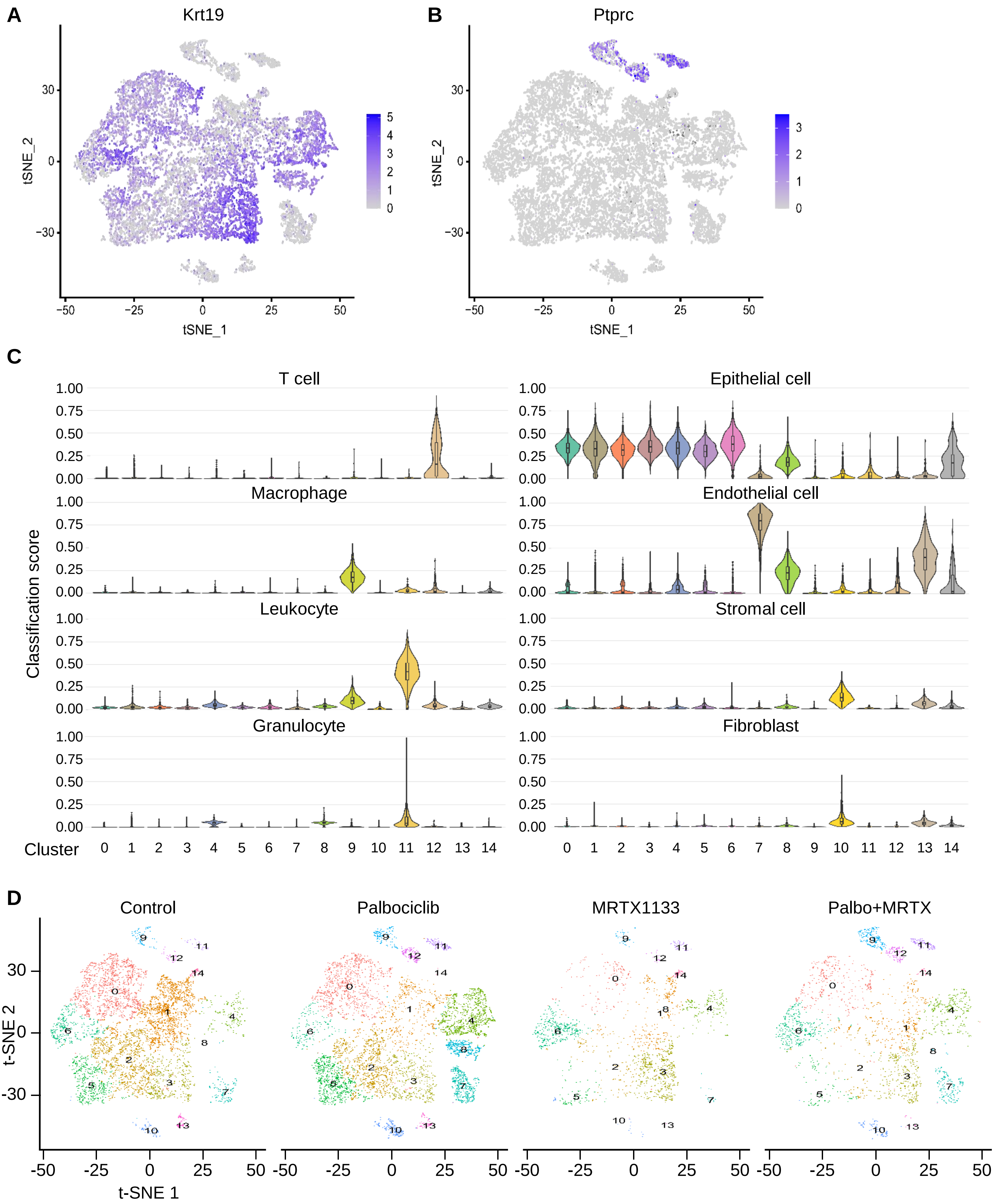

Suppl. Figure 8 continued

E

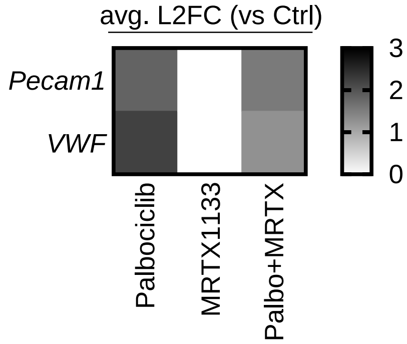

F

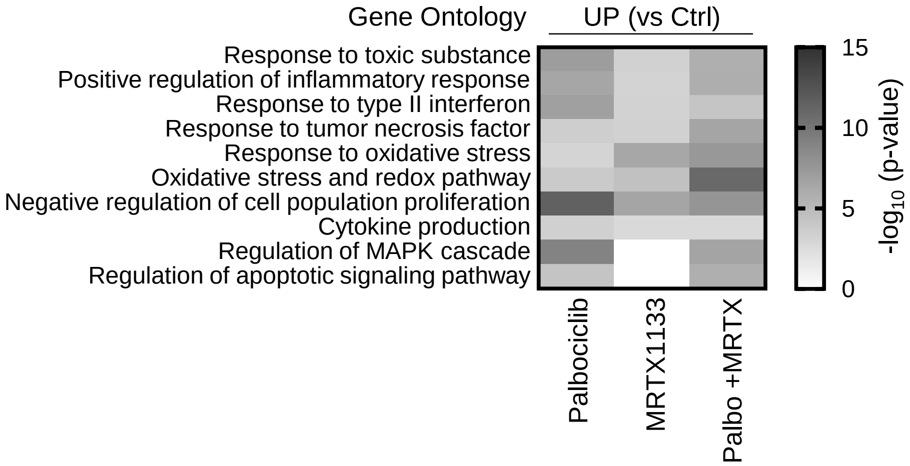

Suppl. Figure 8. Single cell RNA sequencing revealed pronounced cell cycle arrest signatures upon treatment with MRTX1133, and angiogenesis in Palbociclib-treated tumors.

(A) Feature plots of tumor samples with expression of Krt19 (A) and Ptprc (B) to identify different cell populations.

(C) Violin plot representing the cell type score per cluster as obtained from the automatic classifier SingleCellNet. Higher score represents increased similarity to the respective cell type. Different colors represent the clusters from the presented tSNE in Figure 8A.

(D) tSNE visualization of the clusters derived from single cell RNA sequencing of the mouse tumors split per condition corresponding to Figure 8A.

(E) Average expression of Pecam1 and VWF as markers for vascularization of Palbociclib, MRTX1133 or combination versus vehicle derived from Suppl. Table 3: |log2fold| ≥ 0.25.

(F) Analysis performed as in Figure 8C for upregulated genes in tumor cell clusters 0-6 and 8 (top 3000).

Suppl. Table 1:
Transcriptome analysis, 51T-2D cells, cf. Figure 3A. Cells were treated for 48 h (Day3) with DMSO, 5 µM Palbociclib, 2.5 µM Sotorasib or combination or 48 h of treatment + 4 days washout (Day7), (n=3). Table contains DE genes and normalized read counts. Only genes with |log2fold| ≥ 0.6, adjusted p-value (padj.) < 0.05, and baseMean ≥ 15 were included in the analysis.

Suppl. Table 2:
Transcriptome analysis, AsPC-1 cells, cf. Figure 4A. Cells were treated for 24 h with DMSO, 5 µM Palbociclib, 0.5 µM MRTX1133 or combination, (n=3). Table contains DE genes and normalized read counts. Only genes with |log2fold| ≥ 0.6, adjusted p-value (padj.) < 0.05, and baseMean ≥ 15 were included in the analysis.

Suppl. Table 3:
Single cell RNA sequencing analysis of tumors, cf. Figure 8A. n=2 animals per treatment group (vehicle, Palbociclib, MRTX1133 or combination). Only genes with |log2fold| ≥ 0.25 and adjusted p-value (padj.) < 0.05 were included in the analysis. Table contains DE genes of all 15 clusters and of tumor clusters 0-6 and 8 separately.
