# Supplementary material for: Simultaneous targeting of KRAS and CDK4 synergistically induces durable growth arrest in pancreatic cancer cells
Source: Cell Death Dis. 2025 Dec 23;17(1):129. doi: 10.1038/s41419-025-08362-w (PMC12847887; doi:10.1038/s41419-025-08362-w)
Supplement: Supplementary file 5 — Uncropped Western blots [file 41419_2025_8362_MOESM5_ESM.pdf]

Figure 5

A

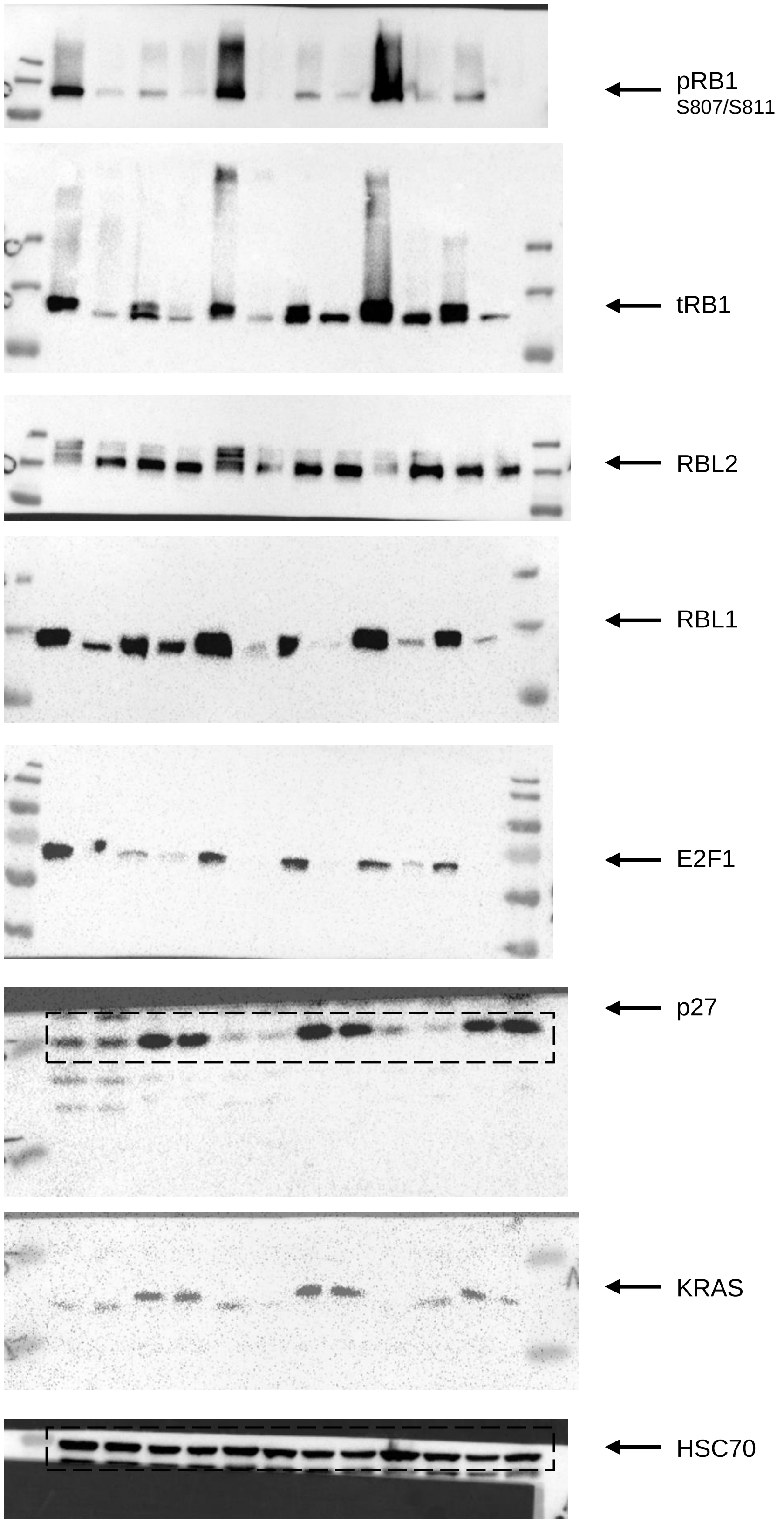

CyclinD1 was stained on the membrane first and covered as signal was strong

tAKT was stained on the membrane first and covered as signal was strong

Figure 5

B

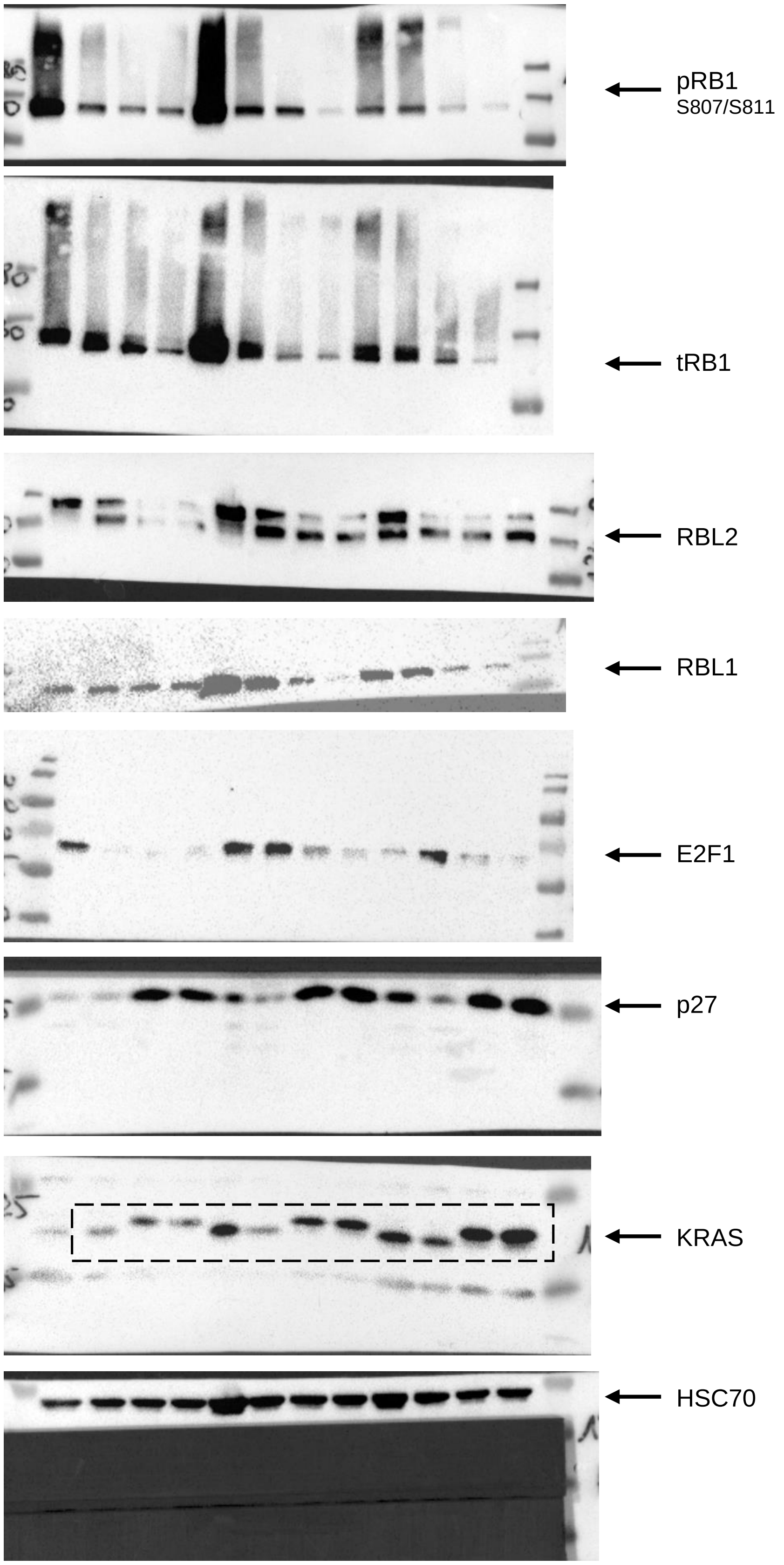

CyclinD1 was stained on the membrane first and covered as signal was strong

tAKT was stained on the membrane first and covered as signal was strong

Figure 5

C

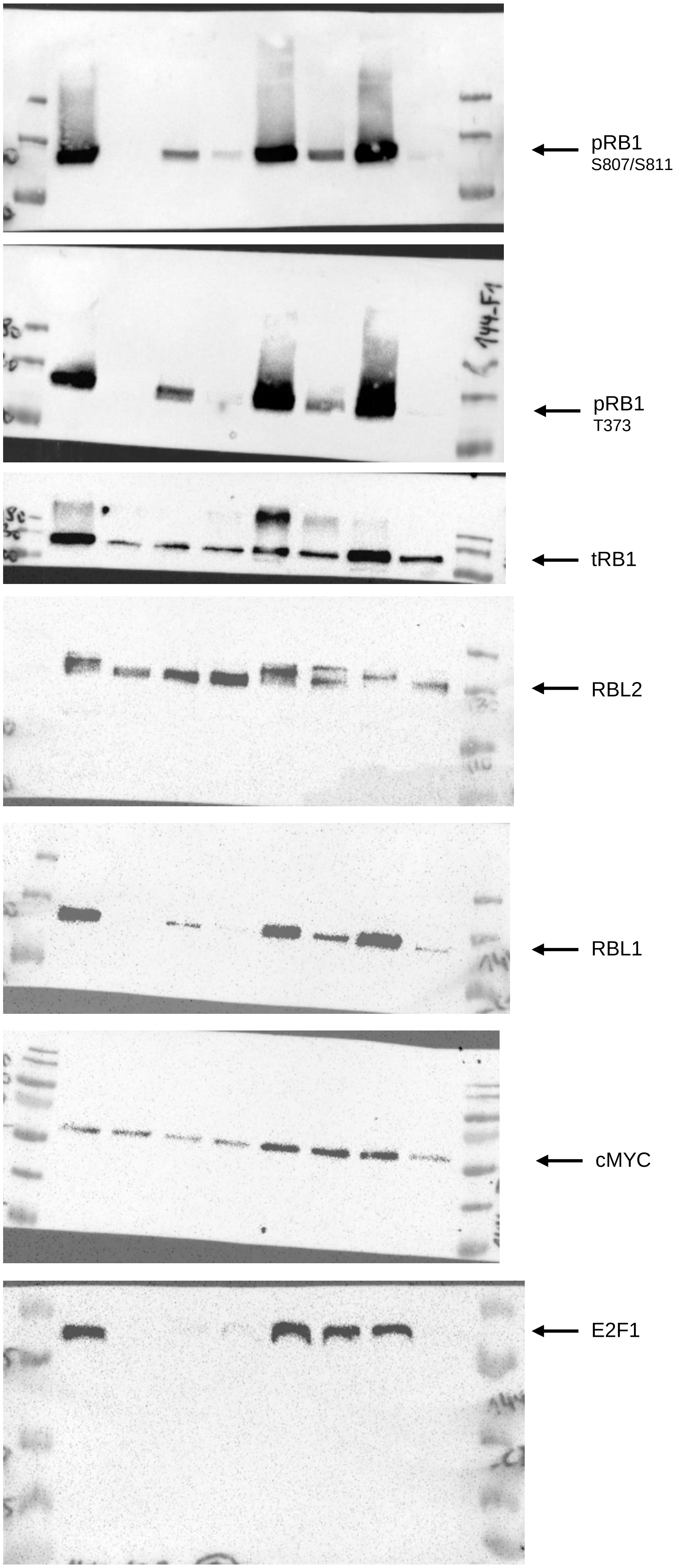

Figure 5

C continued

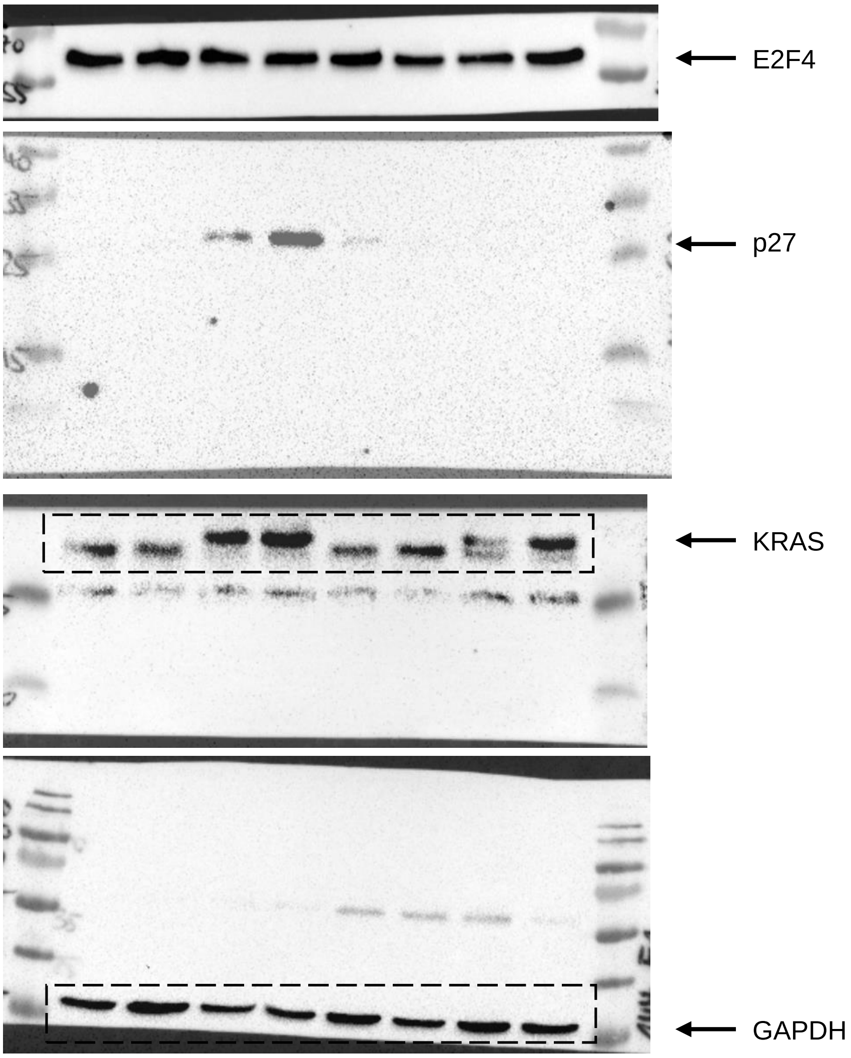

cMYC was stained on the membrane first and not covered

Suppl. Figure 1

O

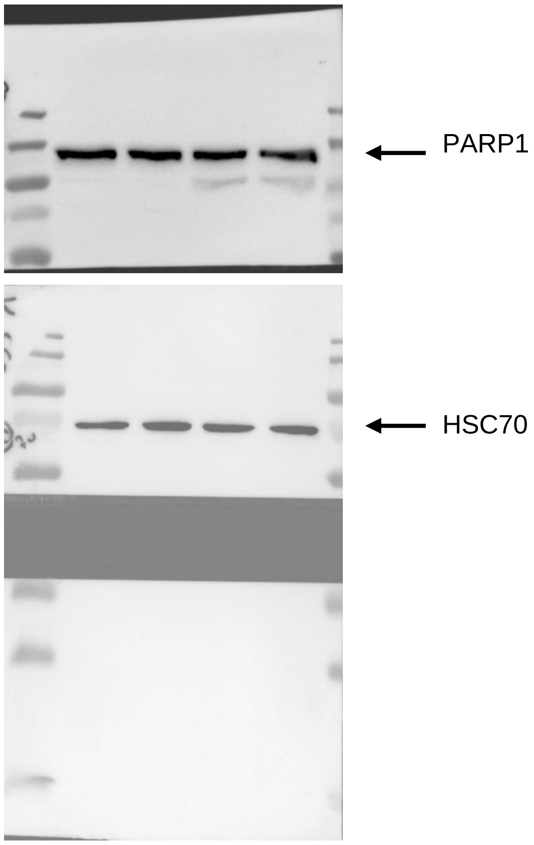

tERK was stained on the membrane first and covered as signal was strong

Suppl. Figure 2

N

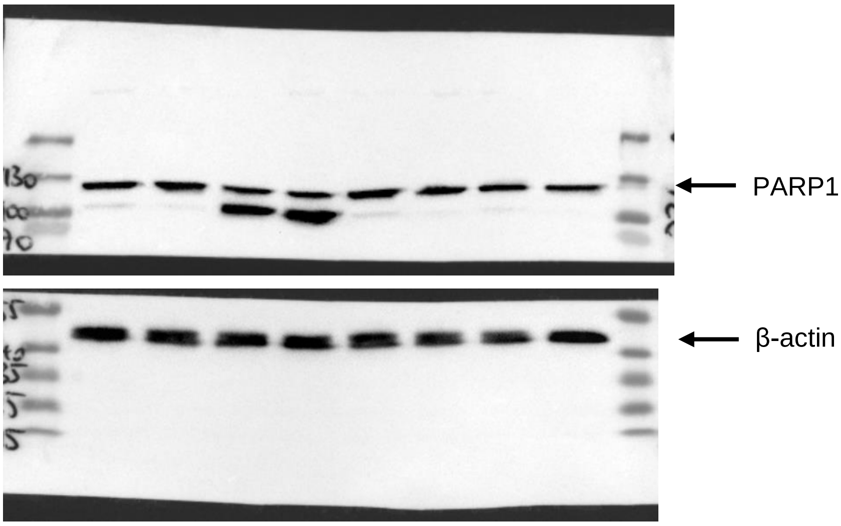

Suppl. Figure 5

A

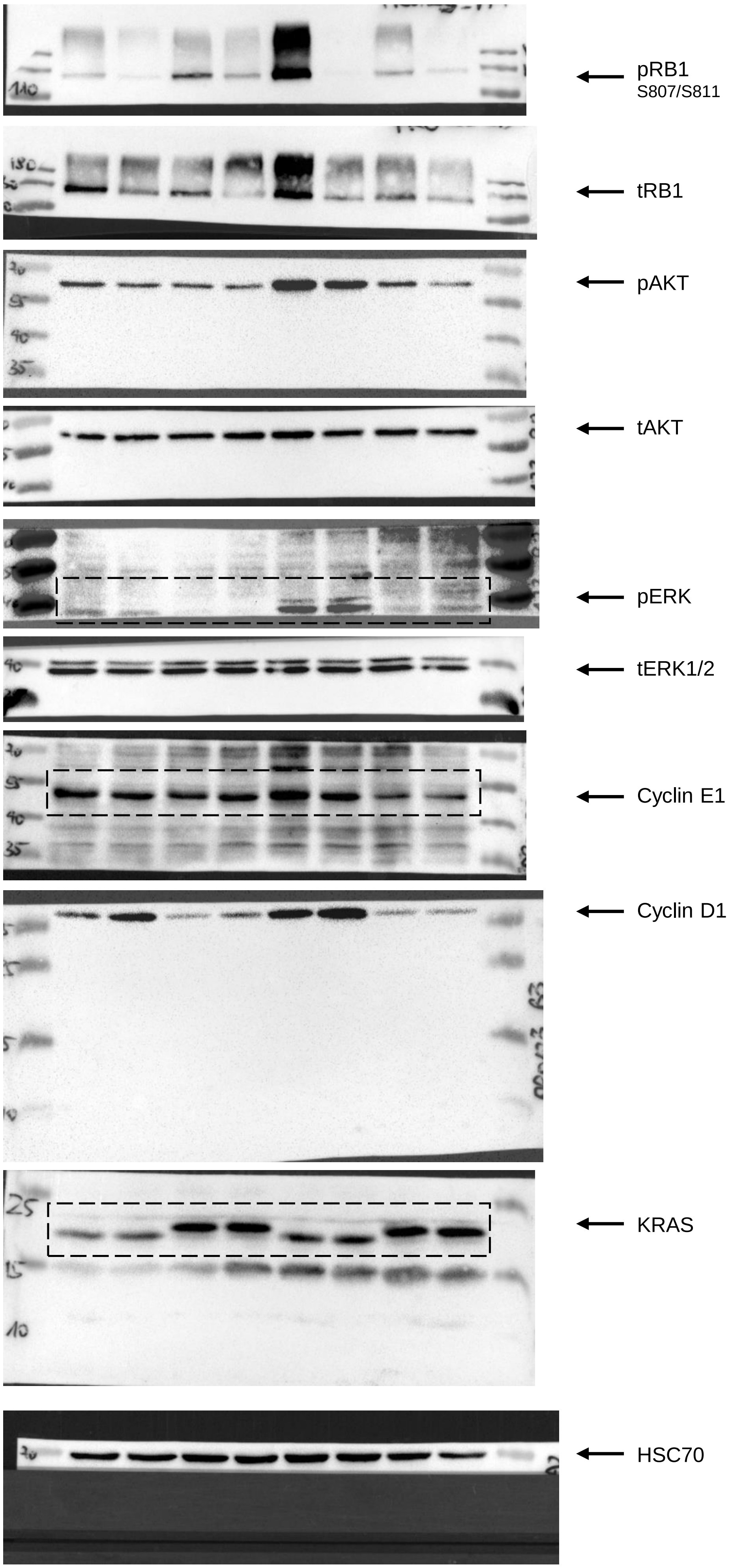

tERK and pAKT were stained on the membrane first and covered as signals were strong

Suppl. Figure 5

B

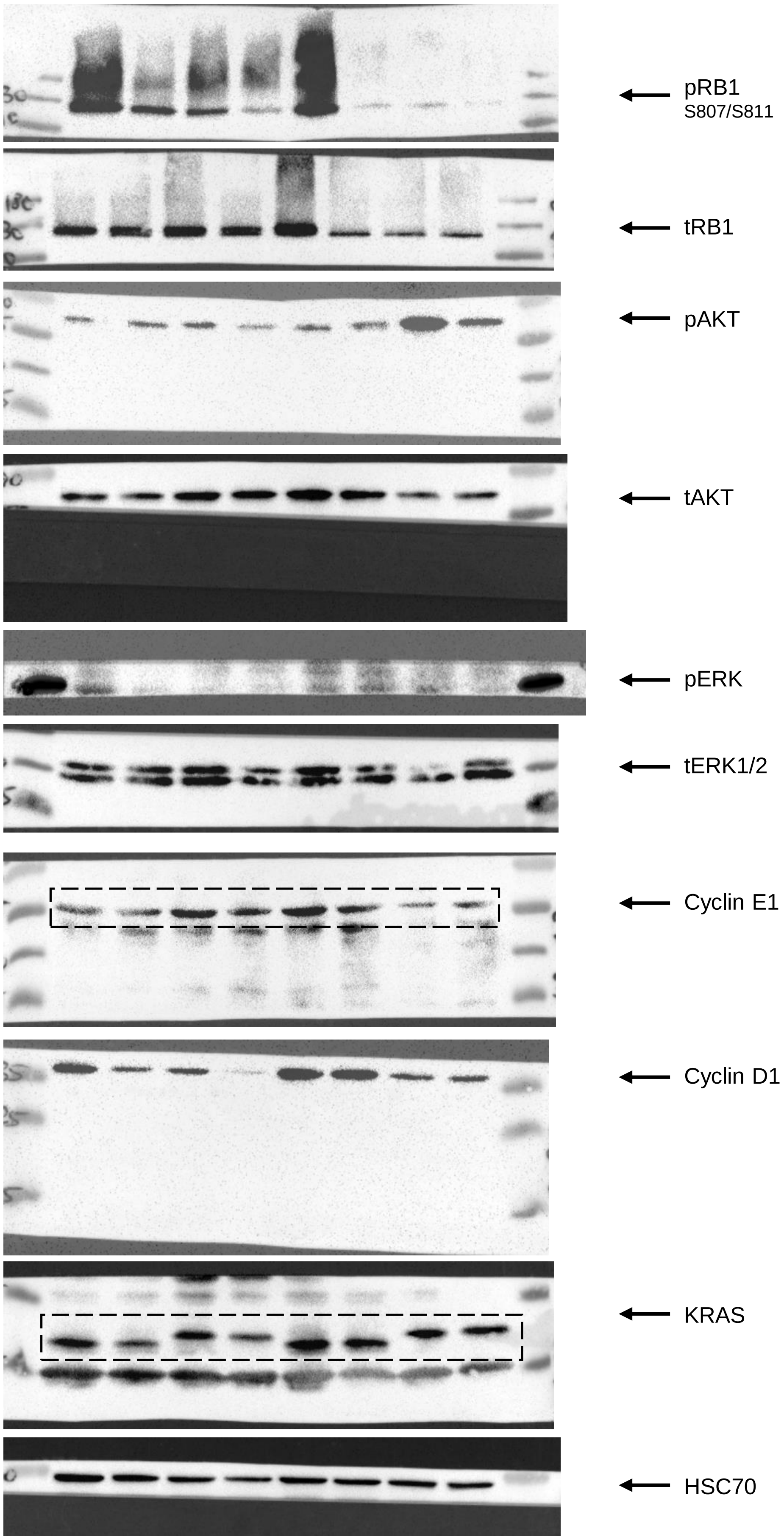

tERK was stained on the membrane first and covered as signal was strong

pERK and tAKT were stained on the membrane first and covered as signals were strong

Suppl. Figure 5

C

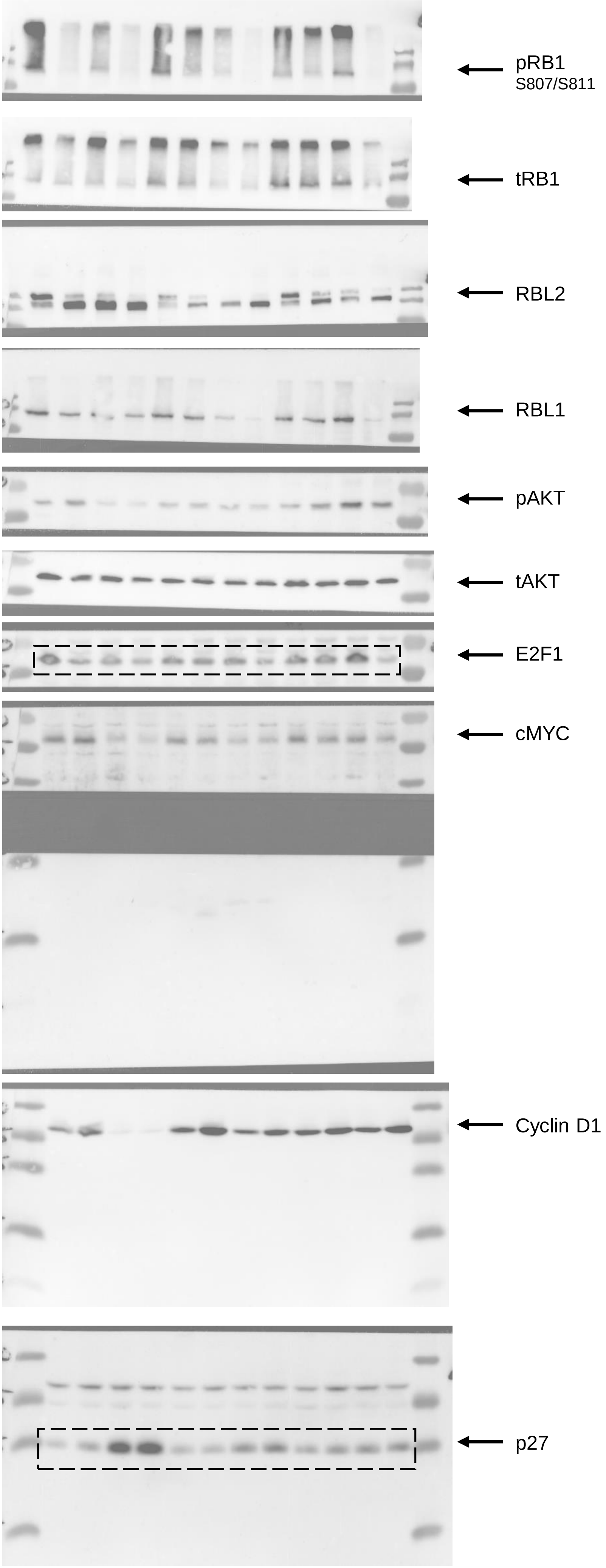

GAPDH was stained on the membrane first and covered as signal was strong

Suppl. Figure 5 - continued

C

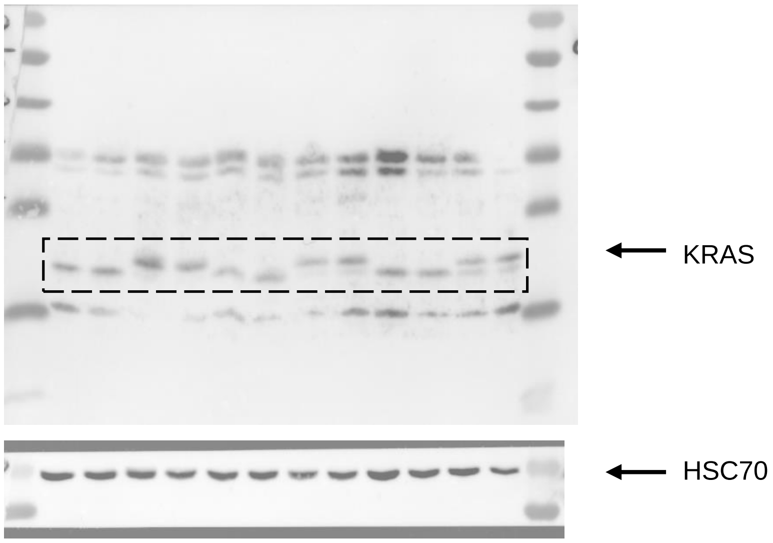

Suppl. Figure 5

D

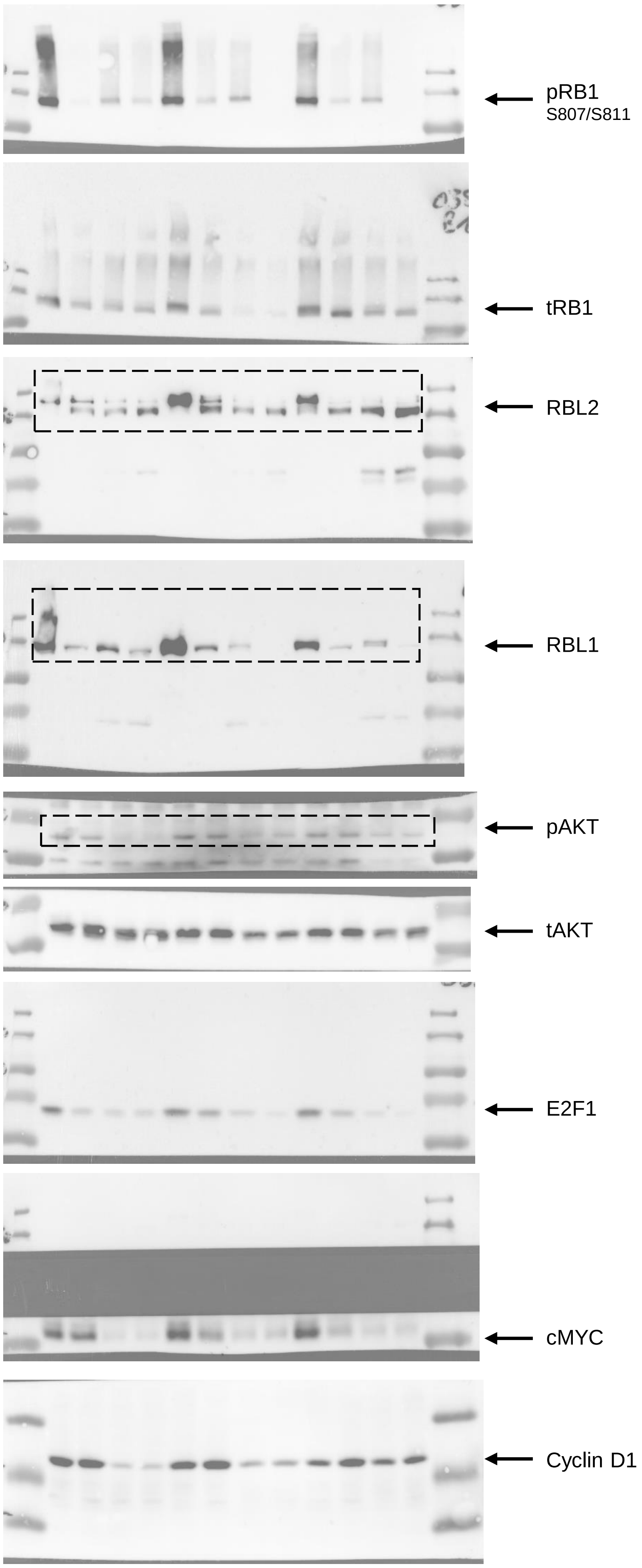

HSC70 was stained on the membrane first and covered as signal was strong

Suppl. Figure 5 - continued

D

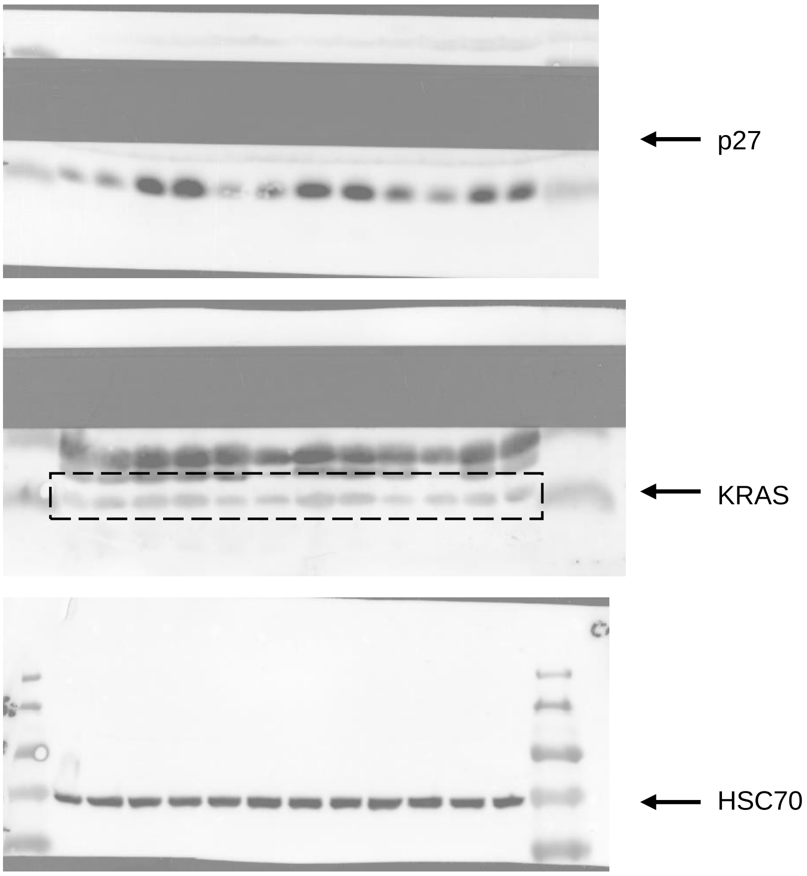

GAPDH was stained on the membrane first and covered as signal was strong

Suppl. Figure 6

B

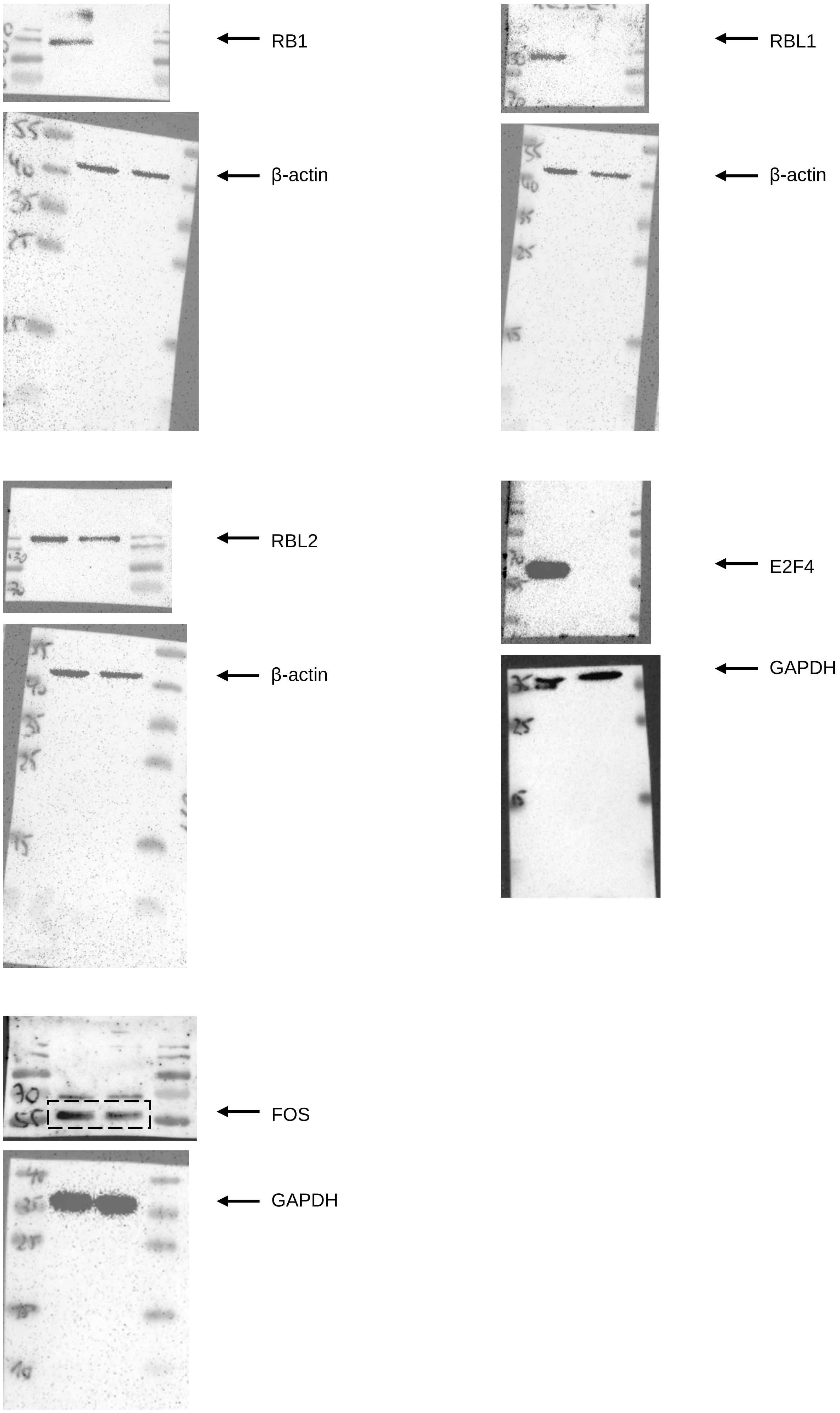

Suppl. Figure 6

D

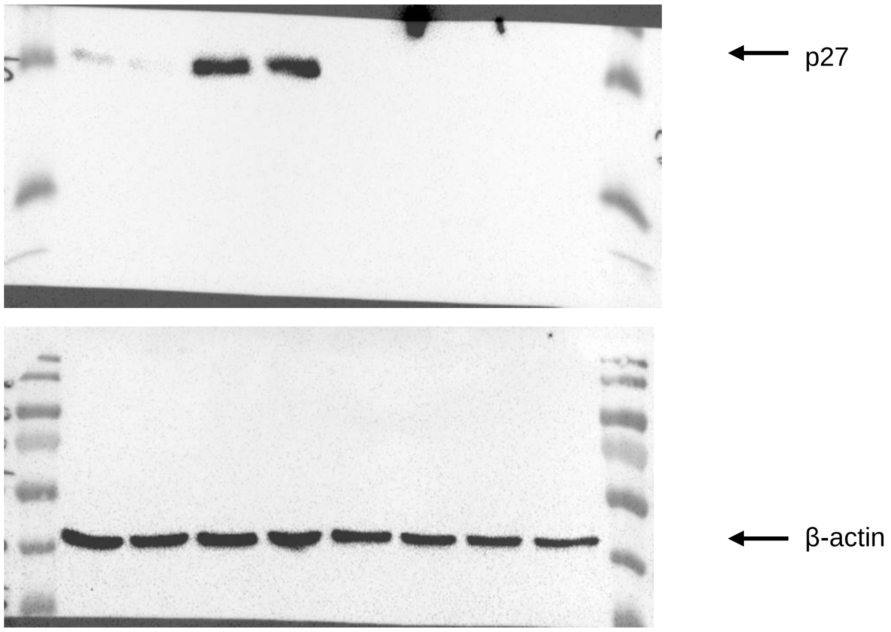

E

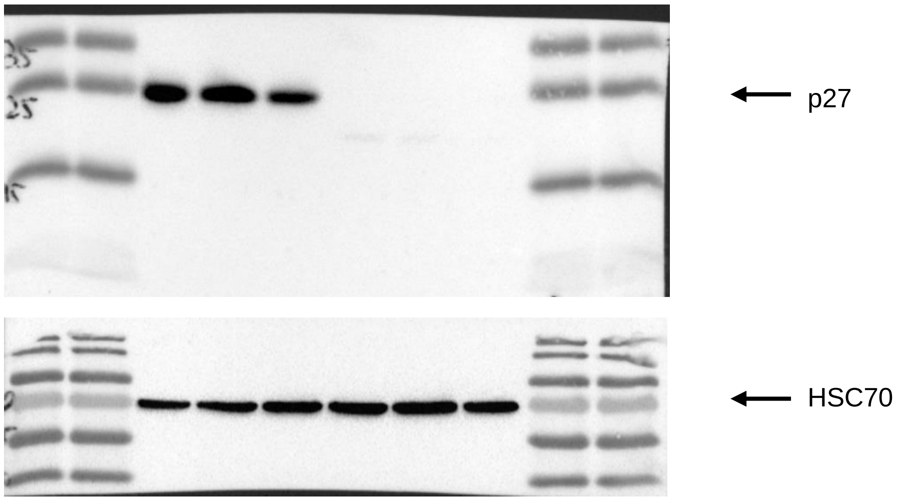

Suppl. Figure 7

X

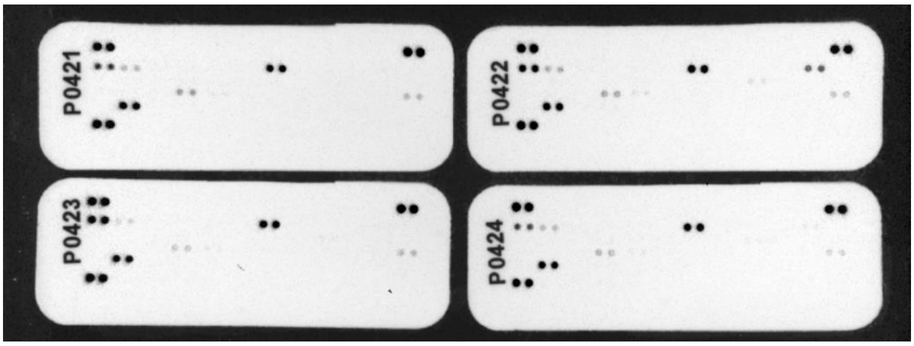

Membrane labels:
P0421: vehicle
P0422: Palbociclib
P0423: MRTX1133
P0424: Palbo+MRTX

Y

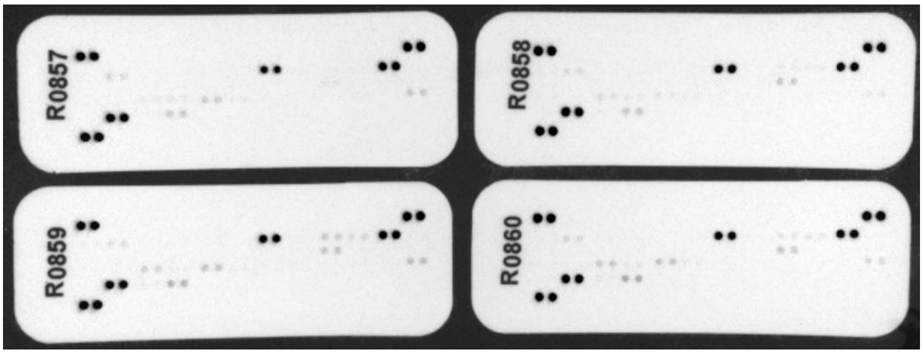

Membrane labels:
R0857: vehicle
R0858 : Palbociclib
R0859 : MRTX1133
R0860: Palbo+MRTX
